# Silyl-Osmium(IV)-Trihydride Complexes Stabilized by a Pincer Ether-Diphosphine: Formation and Reactions with Alkynes

**DOI:** 10.1021/acs.organomet.2c00201

**Published:** 2022-07-18

**Authors:** Juan C. Babón, Miguel A. Esteruelas, Enrique Oñate, Sonia Paz, Andrea Vélez

**Affiliations:** Departamento de Química Inorgánica—Instituto de Síntesis Química y Catálisis Homogénea (ISQCH)—Centro de Innovación en Química Avanzada (ORFEO-CINQA), Universidad de Zaragoza—CSIC, 50009 Zaragoza, Spain

## Abstract

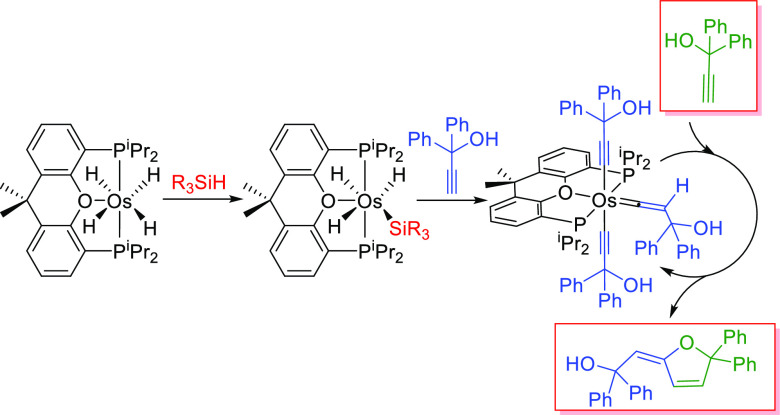

Complex OsH_4_{κ^3^-*P*,*O*,*P*-[xant(P^i^Pr_2_)_2_]} (**1**) activates the Si–H bond of triethylsilane, triphenylsilane, and 1,1,1,3,5,5,5-heptamethyltrisiloxane to give the silyl-osmium(IV)-trihydride derivatives OsH_3_(SiR_3_){κ^3^-*P*,*O*,*P*-[xant(P^i^Pr_2_)_2_]} [SiR_3_ = SiEt_3_ (**2**), SiPh_3_ (**3**), SiMe(OSiMe_3_)_2_ (**4**)] and H_2_. The activation takes place via an unsaturated tetrahydride intermediate, resulting from the dissociation of the oxygen atom of the pincer ligand 9,9-dimethyl-4,5-bis(diisopropylphosphino)xanthene (xant(P^i^Pr_2_)_2_). This intermediate, which has been trapped to form OsH_4_{κ^2^-*P*,*P*-[xant(P^i^Pr_2_)_2_]}(P^i^Pr_3_) (**5**), coordinates the Si–H bond of the silanes to subsequently undergo a homolytic cleavage. Kinetics of the reaction along with the observed primary isotope effect demonstrates that the Si–H rupture is the rate-determining step of the activation. Complex **2** reacts with 1,1-diphenyl-2-propyn-1-ol and 1-phenyl-1-propyne. The reaction with the former affords Os{C≡CC(OH)Ph_2_}_2_{=C=CHC(OH)Ph_2_}{κ^3^-*P*,*O*,*P*-[xant(P^i^Pr_2_)_2_]} (**6**), which catalyzes the conversion of the propargylic alcohol into (*E*)-2-(5,5-diphenylfuran-2(*5H*)-ylidene)-1,1-diphenylethan-1-ol, via (*Z*)-enynediol. In methanol, the hydroxyvinylidene ligand of **6** dehydrates to allenylidene, generating Os{C≡CC(OH)Ph_2_}_2_{=C=C=CPh_2_}{κ^3^-*P*,*O*,*P*-[xant(P^i^Pr_2_)_2_]} (**7**). The reaction of **2** with 1-phenyl-1-propyne leads to OsH{κ^1^-C,η^2^-[C_6_H_4_CH_2_CH=CH_2_]}{κ^3^-*P*,*O*,*P*-[xant(P^i^Pr_2_)_2_]} (**8**) and PhCH_2_CH=CH(SiEt_3_).

## Introduction

Polyhydride complexes, L_*n*_MH_*x*_ (*x* ≥ 3), are transition metal species bearing enough hydrogen atoms bound to the metal center to form both classical hydride and dihydrogen ligands. A noticeable chemical characteristic of the platinum group metal complexes of this class is their proven ability to promote σ-bond activation reactions.^1^ Several features of the H-donor ligands explain the ability of such complexes to break σ-bonds. Classical hydrides behave as Brønsted bases facilitating the heterolytic split, whereas dihydrogen ligands display the tendency to be released from the metal center, generating highly unsaturated species that promote the homolytic cleavage. The activation produces an increase in the coordination number of the metal center of a coordinatively congested species, and in some cases, the oxidation number of an ion already highly oxidized. Consequently, activations involving E–H bonds are favored since the initial coordination number and oxidation of the metal center are rapidly restored after the activation, by removal of molecular hydrogen. Among the activated E–H bonds, the C–H bonds are predominant.^1,2^ On the other hand, the Si–H bond activation has received scarce attention,^3^ although some silyl-metal-polyhydride derivatives are known mainly for osmium^4^ and iridium.^5^ Like the metal-mediated C–H bond cleavage, the Si–H bond activation takes place via σ-intermediates, where the Si–H bond coordinates to the metal center. Nevertheless, a greater variety in the strength degrees has been described for the interactions M–HSi than for the M–HC ones.^6^ The Si–H bond activation reactions are noticeable because of the relevance of the M–SiR_3_ derivatives as intermediate species in the synthesis of chlorosilanes,^7^ the SiH/OH coupling,^8^ and the hydrosilylation of unsaturated organic substrates,^9^ including alkynes.^10^

Alkynes are fundamental molecules in organic synthesis,^11^ which display great relevance in organometallics due to their use as precursors of different functional groups^12^ and as building blocks in the formation of new ligands and interesting metallacycles.^13^ Their reactions with transition metal hydride complexes allow for the generation of single, double, and triple M–C bonds, depending on the nature of the metal center, the ligands, and the substituents of the alkyne.^12^ Increasing the number of hydride ligands of the complex facilitates the use of alkyne building blocks since it permits to increase the number of such molecules accessing into the metal center, as a consequence of the increment of the number of possible reactions. The presence of a higher number of organic fragments attached to the metal favors a higher variety of C–C coupling reactions.^14^ Polyhydride complexes have the ability of activating the C(sp)–H bond of terminal alkynes, whereas they hydrogenate the C–C triple bond of the disubstituted ones. The activation usually leads to alkynyl species, which are able to act as efficient catalyst precursors in the dimerization of such substrates to afford both enynes and butatrienes;^15^ skeletons are of great interest because they are present in some biologically active natural products and in synthetic intermediates for the preparation of highly substituted aromatic rings and by their connection with materials science.^16^ Terminal propargylic alcohols, HC≡CCR^1^R^2^(OH), are alcohol-functionalized alkynes widely employed in organic synthesis as multifunctional reagents^17^ and in organometallics as allenylidene ligand precursors.^18^ Although they are noticeable molecules by both reasons, their reactions with polyhydride complexes have not been studied.

**Scheme 1:**
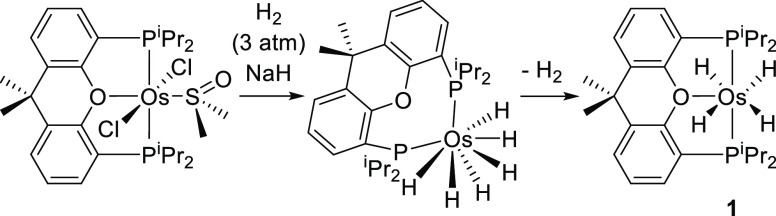
Synthesis of OsH_4_{κ^3^-*P*,*O*,*P*-[xant(P^i^Pr_2_)_2_]}

Pincer ligands are having a dramatic impact in the modern coordination chemistry due to their ability to stabilize less common coordination polyhedra, which favor unusual oxidation states of the metal.^19^ Polyhydride chemistry is not alien to this influence,^19a^ although such ligands have been comparatively much less employed than in other areas, probably because pincer ligands saturate three coordination positions, reducing the number of sites for the hydrides. In this context, hemilabile pincer ligands are of great interest. A particular class with this characteristic is the *P*,*O*,*P*-diphosphines.^20^ Among them, 9,9-dimethyl-4,5-bis(diisopropylphosphino)xanthene (xant(P^i^Pr_2_)_2_) occupies a prominent place due of its coordinating flexibility.^21^ Although the κ^3^-*P*,*O*,*P-mer* mode is its most usual coordination,^8d,21c,22^ complexes bearing diphosphine κ^3^-*P*,*O*,*P-fac*,^21c,23^ κ^2^-*P*,*P-cis*,^24^ and κ^2^-*P*,*P-trans*^21c,25^ are also known. Transformations involving (xant(P^i^Pr_2_)_2_)-M derivatives suggest that this ether-diphosphine changes its disposition at the metal coordination sphere to be adapted to the thermodynamic needs of the reactions. As a result, a wide variety of ruthenium,^22b,22e^ osmium,^22b,22e,22f,22j,23a,23b,24a,24c^ rhodium,^8d,21c,22a,22c,22d,22g,22h,22k,22l,22n,24b,24d^ and iridium^8d,22a,22b,22i,22m,24e^ complexes stabilized by this diphosphine have been reported in the past years, which undergo fascinating transformations and promote interesting reactions, including catalytic processes.^8d,22b−22d,22h,22k−22m,24a,24b,24d,24e,26^ Accordingly, in 2013, we reported that sodium hydride in tetrahydrofuran removes the chloride ligands of OsCl_2_{κ^3^-*P*,*O*,*P*-[xant(P^i^Pr_2_)_2_]}{κ^1^-*S*-[DMSO]} to give the hexahydride derivative OsH_6_{κ^3^-*P*,*O*,*P*-[xant(P^i^Pr_2_)_2_]}, under 3 atm of hydrogen, at 50 °C. This κ^2^-*P*,*P-trans*-diphosphine-osmium(VI)-polyhydride slowly loses a hydrogen molecule in methanol, under an argon atmosphere, at temperatures higher than 293 K. The coordination of the oxygen atom to the metal center stabilizes the tetrahydride derivative OsH_4_{κ^3^-*P*,*O*,*P*-[xant(P^i^Pr_2_)_2_]}. In contrast to its precursor, this tetrahydride bears a κ^3^*-P*,*O*,*P-mer*-diphosphine (Scheme 1).^24a^

The handy availability of the tetrahydride complex along with the hemilability of diphosphine, also proven in the hydride chemistry, as shown in Scheme 1, prompted us to investigate the tetrahydride-promoted Si–H bond activation of silanes. We searched for a silyl-osmium-polyhydride system, allowing us to study the reactivity of such class of complexes with alkynes, including alkynols. This paper describes such activation, including its mechanism, and the reactions of the resulting pohyhydride species with 1,1-diphenyl-2-propyn-1-ol and 1-phenyl-1-propyne.

## Results and Discussion

### Si–H Bond Activation of Tertiary Silanes

**Scheme 2:**
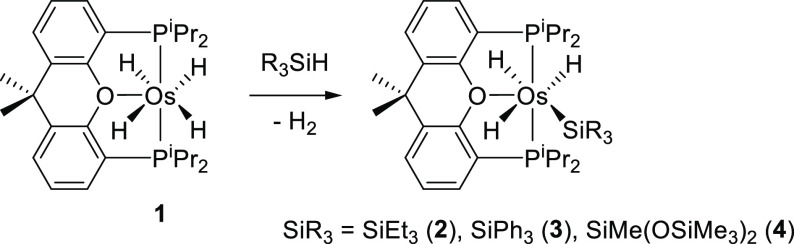
Reaction of OsH_4_{κ^3^-*P*,*O*,*P*-[xant(P^i^Pr_2_)_2_]} with Tertiary Silanes

In spite of its saturated character, tetrahydride-osmium(IV) complex OsH_4_{κ^3^-*P*,*O*,*P*-[xant(P^i^Pr_2_)_2_]} (**1**) activates the Si–H bond of triethylsilane, triphenylsilane, and 1,1,1,3,5,5,5-heptamethyltrisiloxane. Treatment of solutions of this polyhydride in toluene, with 2.0 equiv of the silane, under reflux, for 16 h leads to the corresponding derivatives OsH_3_(SiR_3_){κ^3^-*P*,*O*,*P*-[xant(P^i^Pr_2_)_2_]} [SiR_3_ = SiEt_3_ (**2**), SiPh_3_ (**3**), and SiMe(OSiMe_3_)_2_ (**4**)] and molecular hydrogen. These silyl-osmium(IV)-trihydride compounds were isolated as white solids in a high yield of 77–97%, in accordance with Scheme 2.

**Figure 1:**
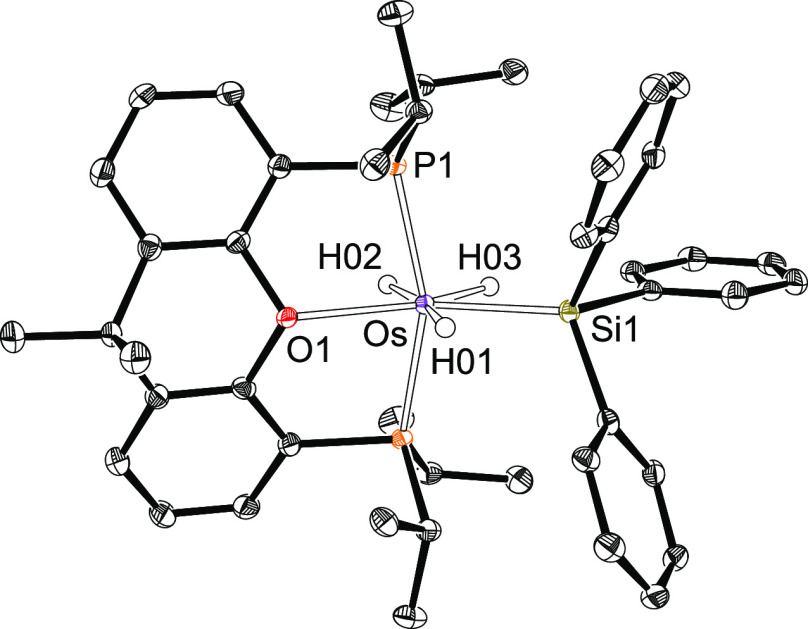
Molecular diagram of complex **3** (ellipsoids shown at 50% probability). All hydrogen atoms (except the hydrides) are omitted for clarity. Selected bond distances (Å) and angles (deg): Os–P(1) = 2.3061(6), Os–O(1) = 2.284(2), Os–Si(1) = 2.3862(9); P(1)–Os–P(1) = 158.63(4), O(1)–Os–Si(1) = 145.97(6).

The presence of a silyl ligand in complexes **2**–**4** is proved by the X-ray structure of the triphenylsilyl derivative **3**. Figure 1 shows the molecular diagram. The polyhedron around the metal center can be idealized as a pentagonal bipyramid. Ether-diphosphine, which is coordinated in the *mer*-fashion, disposes the P^i^Pr_2_ arms at apical positions, forming a P–Os–P angle of 158.63(4)°. The base is defined by the oxygen atom, the silyl group, and the hydride ligands. The oxygen atom is situated between H(01) and H(02), whereas the silyl group lies between H(01) and H(03). The classical-polyhydride nature of these compounds is supported by the separations between the hydrides H(02) and H(03) of 1.71(4) Å, obtained from the X-ray diffraction analysis, and 1.826 Å, calculated for the DFT-optimized structure. Both the X-ray structure and that DFT calculated reveal relatively short separations between the silicon atom and the hydrides H(01) and H(03) of 2.16(3) and 2.21(3) Å and 2.241 and 2.230 Å, respectively, which could suggest the existence of the denoted “*secondary interactions between silicon and hydrogen atoms* (SISHA)” (1.9–2.4 Å).^3,6b^ Nevertheless, atoms in molecules (AIM) calculations do not show any bond path running between the involved atoms (Figure S1). Thus, such values seem to be a consequence of the size of the atoms and their positions in the complex but not of the presence of any bonding interaction between them. In this context, we note that the boron-counterpart OsH_2_(η^2^-H-Bcat){κ^3^-*P*,*O*,*P*-[xant(P^i^Pr_2_)_2_]} (HBcat = catecholborane) is in contrast to the **2**–**4** a hydride-osmium(II)-(σ-borane) derivative.^22f^ Although there is a marked diagonal relationship between boron and silicon,^27^ it seems that the stronger acidity of boron with regard to silicon favors the hydrogen-heteroatom interaction in this case.

Hydrides H(02) and H(03) undergo a thermally activated site exchange process in toluene-*d*_8_, which occurs with low activation energy (Table 1). Thus, at 313 K, the high-field region of the ^1^H NMR spectra of the three polyhydrides shows two resonances in a 1:2 intensity ratio, at about −1 ppm and between −10.0 and −12.5 ppm. The first of them is due to H(01), and the second one corresponds to H(02) and H(03). Between 283 and 223 K, depending on the silyl ligand, decoalescence of the higher field signal takes place to afford two resonances. Accordingly, at temperatures lower than 223 K, the spectra display a resonance for each inequivalent hydride ligand, at the chemical shifts shown in Table 1. The 300 MHz *T*_1(min)_ values for the resonance assigned to the hydride ligands H(02) and H(03) of **2** and **3** were also determined at 248 and 230 K, respectively. The obtained values of 231 ± 3 (**2**) and 251 ± 3 (**5**) ms lead to separations of 1.76 (**2**) and 1.79 (**3**) Å between such hydrides,^28^ which compare very nicely with those obtained from the X-ray diffraction analysis and DFT calculations for **3**. In agreement with the structure shown in Figure 1, the ^31^P{^1^H} NMR spectra of **2**–**4** show a singlet between 54 and 47 ppm, as expected for the equivalent P^i^Pr_2_ arms of diphosphine. A characteristic feature of these compounds is also the presence of a triplet (^2^*J*_Si–P_ ≈ 5 Hz) at about 2 ppm for **2** and **3** and at −19.8 ppm for **4**, in the respective ^29^Si{^1^H} NMR spectrum.

**Table 1:** Coalescence Temperature (*T*_c_) and Activation Energy (Δ*G*^‡^) for the Site Exchange Process and NMR Chemical Shift for the Hydride Ligands of Complexes **2–4**

complex	T_c_ (K)	Δ*G*^‡^ (kcal·mol^–1^)	chemical shift (ppm)		
**2**	278	10.9 ± 0.2	–1.76	–4.01	–20.21
**3**	248	9.8 ± 0.2	–0.14	–3.73	–17.08
**4**	228	8.9 ± 0.2	–1.53	–4.42	–18.85

### Mechanism of the Si–H Bond Activation

**Scheme 3:**
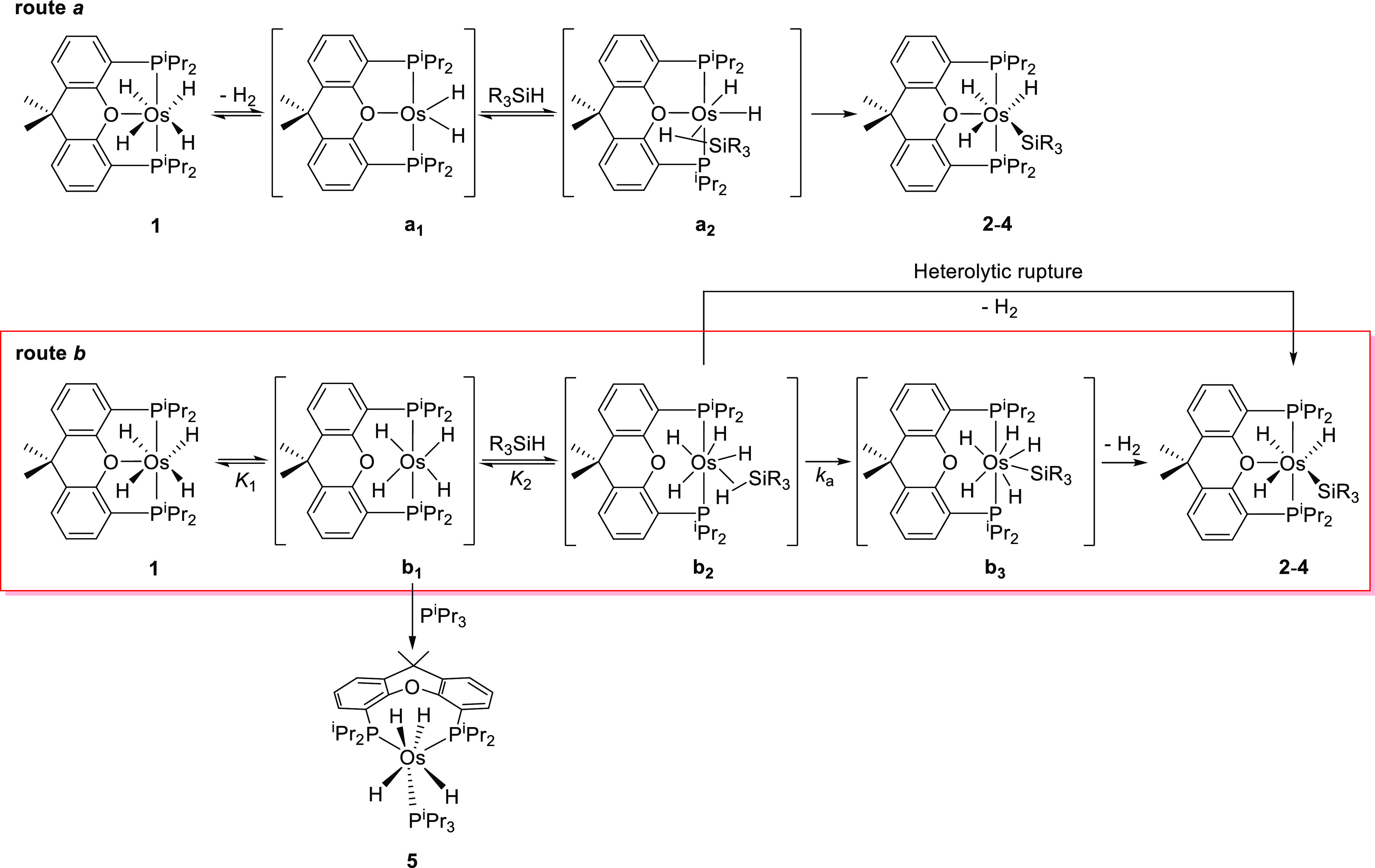
Proposed Reaction Pathway for the OsH_3_(SiR_3_){κ^3^-*P*,*O*,*P*-[xant(P^i^Pr_2_)_2_]} Formation

Complex **1** is a coordinately saturated species. Thus, the Si–H bond activation of the silanes requires the previous generation of a coordination vacancy at the metal center. In principle, such a process could occur in two different manners (Scheme 3): by reductive elimination of molecular hydrogen (route *a*) and through the dissociation of the hemilabile ether function of diphosphine (route *b*). In the first case, the activation would take place via osmium(II) intermediates; the unsaturated dihydride **a**_**1**_ should coordinate the Si–H bond of the silanes to afford the osmium(II) σ-intermediates **a**_**2**_, which could evolve into **2**–**4** by homolytic rupture of the coordinate σ-bond. In the second one, the activation involves intermediates of osmium(IV) and osmium(VI); similarly to **a**_**1**_, the unsaturated osmium(IV)-tetrahydride **b**_**1**_ should coordinate the Si–H bond of the silanes to give the osmium(IV) σ-intermediates **b**_**2**_. These intermediates could lead to **2–4** by means of two different pathways associated to the type of activation undergone by the coordinated σ-bond. A hydride-promoted heterolytic rupture should directly give the silyl-osmium(IV)-trihydride derivatives, whereas a homolytic activation followed by reductive elimination of molecular hydrogen would afford the silyl products via silyl-osmium(VI)-pentahydride intermediates **b**_**3**_. Complexes with two triisopropylphosphine ligands instead of ether-diphosphine resembling to **b**_**3**_ have been recently isolated and fully characterized, including the X-ray analysis structure of one of them in our laboratory.^29^

We reasoned that the use of R_3_SiD instead of R_3_SiH should allow us to discern between routes *a* and *b* and establish the nature of the Si–H rupture. Indeed, route *a* should afford one deuterium atom at the hydride position, whereas a hydride-promoted heterolytic rupture through route *b* should lead to a totally protiated product. On the other hand, the homolytic activation to form intermediate **b**_**3**_ would permit a deuterium amount intermediate between zero and one at the metal center since the position exchange processes, typical in this class of pentahydride complexes, should carry the deuterium atom to different positions, before the reductive elimination. Addition of 2.0 equiv of (Me_3_SiO)_2_MeSiD to a solution of **1** in toluene-*d*_8_, at 110 °C leads to a partially deuterated **2-*d***_**0.5**_ species containing 0.5 deuterium atoms distributed between the H(02) and H(03) positions (Figure S49). As mentioned above, such a deuterium amount at the metal center supports the route *b* through intermediate **b**_**3**_ as the preferred one for the Si–H bond activation promoted by **1**.

**Figure 2:**
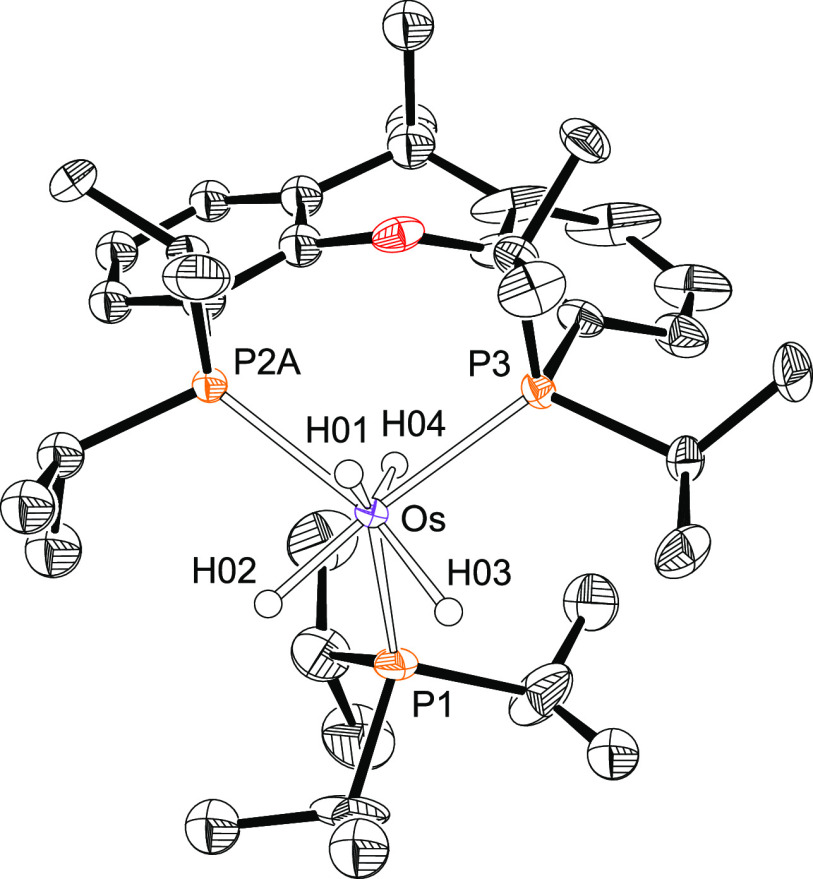
Molecular diagram of complex **5** (ellipsoids shown at 50% probability). All hydrogen atoms (except the hydrides) are omitted for clarity. Selected bond distances (Å) and angles (deg): Os–P(1) = 2.3128(9), Os–P(2A) = 2.3433(11), Os–P(3) = 2.3540(7); P(2A)–Os–P(3) = 105.43(4), P(1)–Os–P(3) = 115.40(3).

To gain additional evidence in favor of the route *b*, we decided to trap the intermediate **b**_**1**_ with a 2e-donor ligand such as triisopropylphosphine. In methanol, the stirring of **1** in the presence of 1.0 equiv of phosphine affords the expected tetrahydride OsH_4_{κ^2^-*P*,*P*-[xant(P^i^Pr_2_)_2_]}(P^i^Pr_3_) (**5** in Scheme 3), which was isolated as a white solid in 78% yield and characterized by X-ray diffraction analysis. Figure 2 gives a view of the structure. The disposition of donor atoms around the metal center can be described as a piano stool geometry of the ideal *C*_*s*_ symmetry, where the four-membered face is formed by the phosphorus atoms of the chelating diphosphine and the hydrides H(01) and H(04), whereas the three-membered face is defined by the hydrides H(02) and H(03) and the phosphorus atom of triisopropylphosphine. Such ligand disposition is unusual for osmium(IV)-tetrahydride complexes of the class OsH_4_(PR_3_)_3_, which generally displays a pentagonal bipyramid arrangement.^30^ In solution of toluene-*d*_8_, the structure is not rigid in accordance with the similar stability of the usual four geometries of seven-coordinate complexes and the low activation energy for their interconversion.^31^ Thus, the ^1^H NMR spectrum at room temperature shows a broad resonance for the four hydride ligands at −10.87 ppm, which splits into two broad signals, at −9.99 and −11.90 ppm, of 1:1 intensity ratio, at 235 K. The ^31^P{^1^H} spectrum is also temperature-dependent (Figure S27). At room temperature, the spectrum shows at 44.6 ppm an apparent triplet due to monodentate phosphine and at 8.4 ppm a broad signal corresponding to diphosphine. At temperatures lower than 253 K, the triplet is transformed into a complex signal, whereas the broad resonance splits into two signals at 14.7 and 2.3 ppm.

**Figure 3:**
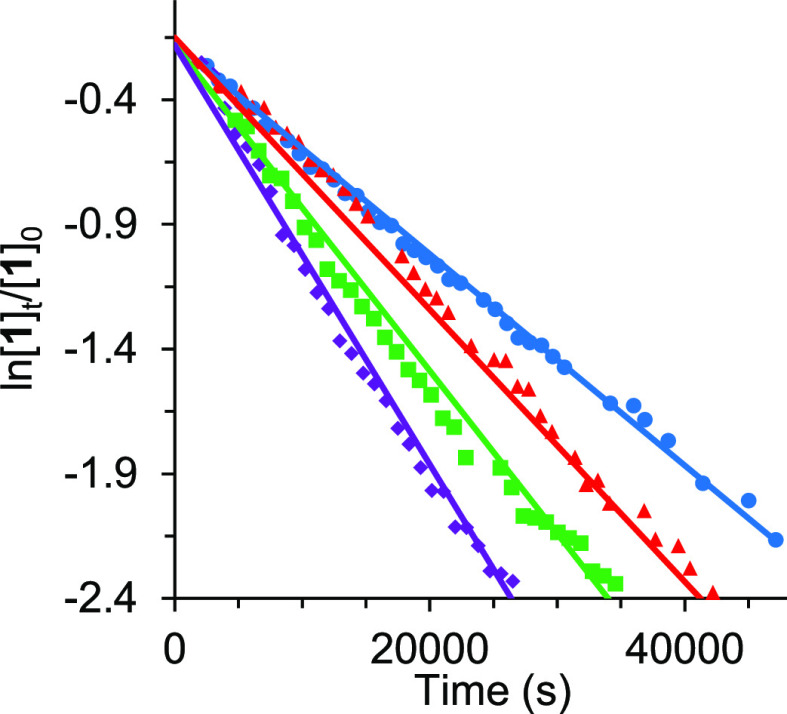
Plot of eq 1 for reaction of **1** (1.9 × 10^–2^ M) with different concentrations of triethylsilane in toluene-*d*_8_. [Et_3_SiH] = 0.38 M (blue ●); 0.48 M (red ▲); 0.57 M (green ■); 0.76 M (purple ⧫).

Once the pathway for the Si–H bond activation was established, we decided to confirm it and ascertain the rate-determining step by means of the kinetics investigation of the reaction of **1** with Et_3_SiH. The transformation of **1** into **2**, studied by ^31^P{^1^H} NMR spectroscopy, was performed under *pseudo*-first order conditions, at 353 K, for concentrations of silane ([Et_3_SiH]) between 0.38 and 0.76 M, and starting from a concentration of **1** of 1.9 × 10^–2^ M ([**1**]_0_). In this range of silane concentrations, the consumption of **1** with the corresponding increase of the amount of **2** is an exponential function of time, which can be linearized (Figure 3) according to the expression shown in eq 1, where [**1**] is the concentration of tetrahydride at the time *t*. Table 2 shows the rate constants *k*^obs^ for each silane concentration.
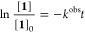
1

**Table 2:** Kinetic Data of the Reaction of 1 with Et_3_SiH in Toluene-*d*_8_

*T* (K)	[Et_3_SiH] (M)	*k*^obs^ (×10^5^ s^–1^)	k (×10^4^ M^–1^ s^–1^)
353	0.38	4.24 ± 0.10	1.11 ± 0.10
353	0.48	5.46 ± 0.11	1.15 ± 0.11
353	0.57	6.53 ± 0.19	1.15 ± 0.19
353	0.76	8.41 ± 0.24	1.10 ± 0.24

**Figure 4:**
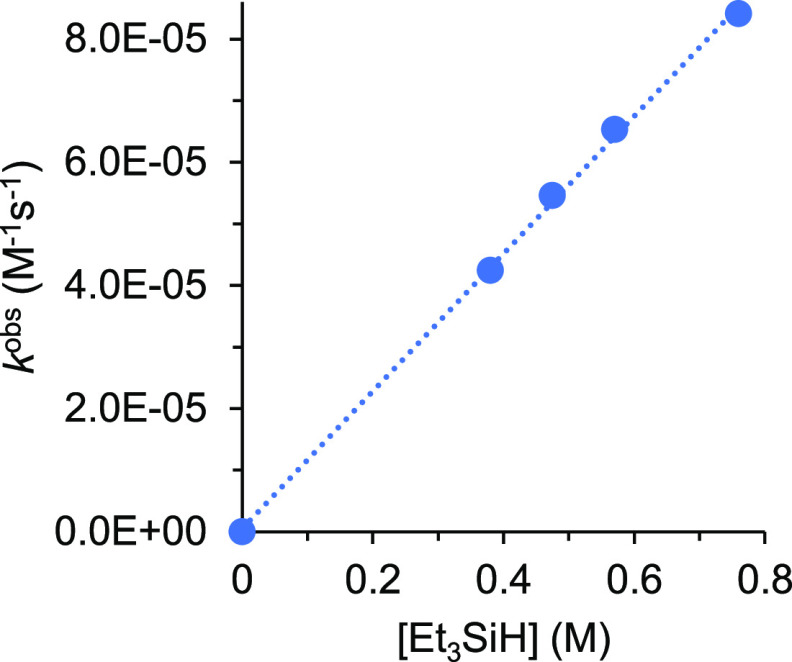
Plot of *k*^obs^ vs [Et_3_SiH] for the reaction of **1** (0.019 M) with Et_3_SiH in toluene-*d*_8_ at 353 K.

Constant *k*^obs^ is a function of [Et_3_SiH] according to eq 2. A plot of ln *k*^obs^ versus ln[Et_3_SiH] yields a straight line of slope 0.98 (Figure S54), revealing that the activation is also first-order in the silane concentration (*a* = 1 in eq 2), and therefore, the rate law is that given in eq 3. A plot of *k*^obs^ versus [Et_3_SiH] (Figure 4) provides a value of (1.1 ± 0.1) × 10^–4^ M^–1^ s^–1^ for *k*.
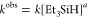
2
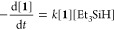
3

The rate of the reaction of **1** with Et_3_SiD is significantly slower than that with Et_3_SiH. The ratio *k*_H_/*k*_D_ gives a primary isotope effect of 4.2, which strongly supports the homolytic cleavage of the Si–H bond as the rate-determining step in the formation of the silyl-osmium(IV)-trihydride complexes.^32^ According to the rate-determining step approximation, the formation of **2** can be also described by eq 4.
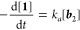
4

Since the reductive elimination of molecular hydrogen from intermediate ***b***_**3**_ is the fast step of the silyl product formation, the concentration of the intermediate ***b***_**2**_ can be calculated as
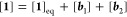
5

Because [**1**]_eq_ = [***b***_**2**_]/*K*_1_*K*_2_[Et_3_SiH] and [***b***_**1**_] = [***b***_**2**_]/*K*_2_[Et_3_SiH], we have
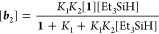
6

Intermediate ***b***_**1**_ is not spectroscopically detected. As a consequence, we can assume that *K*_1_ + *K*_1_*K*_2_[Et_3_SiH] ≪ 1, and therefore, [**b**_**2**_] can be described as
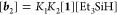
7

Combining eqs 4 and 7, we obtain eq 8, which is additional evidence in favor of route *b* and reveals that *k* = *k*_a_*K*_1_*K*_2_, that is, the experimental rate constant *k* is proportional to the rate constant *k*_a_ and the equilibrium constants *K*_1_ and *K*_2_.
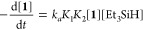
8

### Reactions with Alkynes

**Scheme 4:**
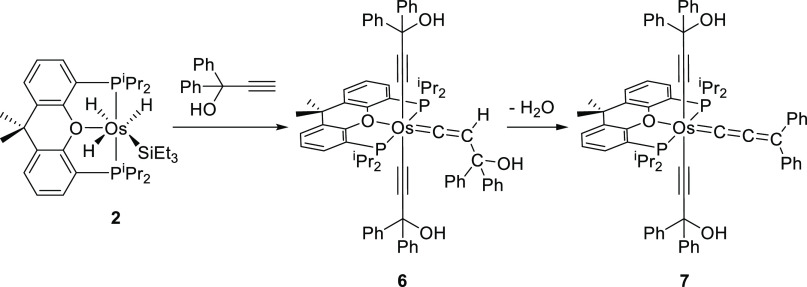
Reaction of OsH_3_(SiEt_3_){κ^3^-*P*,*O*,*P*-[xant(P^i^Pr_2_)_2_]} with 1,1-Diphenyl-2-propyn-1-ol

Treatment of solutions of **2** in toluene with 4.0 equiv of 1,1-diphenyl-2-propyn-1-ol, at 80 °C, for 2 h leads to the hydroxyvinylidene-osmium(II)-(bishydroxyalkynyl) derivative Os{C≡CC(OH)Ph_2_}_2_{=C=CHC(OH)Ph_2_}{κ^3^-*P*,*O*,*P*-[xant(P^i^Pr_2_)_2_]} (**6**), which was isolated as a brown solid in 52% yield. In methanol, the hydroxyvinylidene ligand dehydrates. Thus, the stirring of suspensions of **6** in alcohol, for 4 h, at room temperature affords the allenylidene complex Os{C≡CC(OH)Ph_2_}_2_{=C=C=CPh_2_}{κ^3^-*P*,*O*,*P*-[xant(P^i^Pr_2_)_2_]} (**7**), which was obtained as a green solid in 85% yield (Scheme 4).

**Figure 5:**
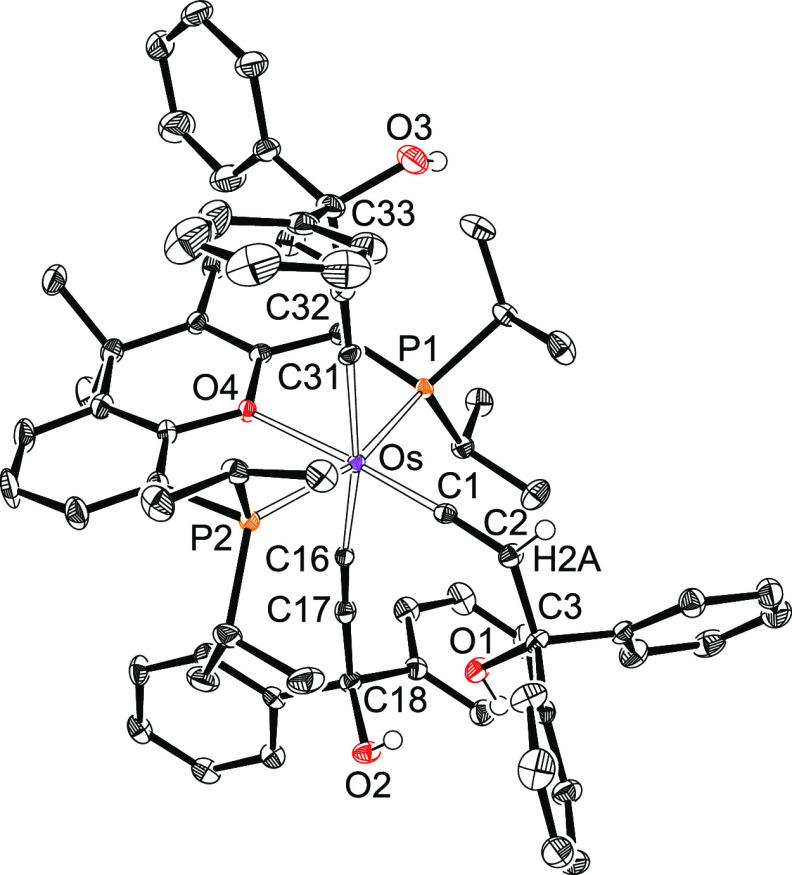
Molecular diagram of complex **6** (ellipsoids shown at 50% probability). All hydrogen atoms (except the C_β_–H and OH) are omitted for clarity. Selected bond distances (Å) and angles (deg): Os–P(1) = 2.3489(7), Os–P(2) = 2.3373(7), Os–O(4) = 2.262(2), Os–C(16) = 2.068(3), Os–C(31) = 2.068(3), Os–C(1) = 1.820(3), C(1)–C(2) = 1.316(4); P(1)–Os–P(2) = 162.36(3), C(1)–Os–O(4) = 176.13(10), C(16)–Os–C(31) = 168.57(12).

Complex **6** was characterized by X-ray diffraction analysis. Figure 5 shows a view of the structure. With the ether-diphosphine *mer*-disposed [P(1)–Os–P(2) = 162.36(3)°], the coordination polyhedron defined by the donor atoms around the metal center is the expected octahedron for a six-coordinate *d*^*6*^-species. The hydroxyalkynyl ligands are mutually *trans*-situated, forming an angle C(16)–Os–C(31) of 168.57(12)°, whereas the π-acceptor hydroxyvinylidene group lies *trans* to the π-donor oxygen atom of the pincer [C(1)–Os–O(4) = 176.13(10)°]. The metal-alkynyl bond lengths Os–C(16) and Os–C(31) of 2.068(3) Å are consistent with an Os–C(sp) single bond^33^ and suggest a low degree of osmium-to-ligand back-bonding.^34^ As in other osmium-vinylidene compounds,^35^ the vinylidene ligand binds to the osmium atom in a nearly linear fashion establishing an angle Os–C(1)–C(2) of 175.2(3)°. Distances Os–C(1) and C(1)–C(2) of 1.820(3) and 1.316(4) Å, respectively, also compare well with those previously reported for complexes of this class and strongly support the presence of double bonds between the involved atoms. In benzene-*d*_*6*_, at room temperature, the vinylidene ligand rotates around the metal-vinylidene axis, as is usual for this class of compounds. Thus, the ^13^C{^1^H} NMR spectrum reveals the presence of only one type of hydroxyalkynyl ligand; the atoms C_α_ and C_β_ of the triple bond give rise to a triplet (^2^*J*_C–P_ = 11.8 Hz) at 102.0 ppm and a singlet at 75.5 ppm, respectively, while the C_α_ and C_β_ atoms of the vinylidene ligand generate two triplets, at 291.6 (^2^*J*_C–P_ = 9.1 Hz) and 111.5 (^3^*J*_C–P_ = 3.8 Hz) ppm, respectively. In the ^1^H NMR spectrum, the most noticeable resonance is that due to the C_β_H-hydrogen atom of the vinylidene moiety, which appears at 2.45 ppm as a triplet (^4^*J*_H–P_ = 2.7 Hz). The ^31^P{^1^H} NMR spectrum displays a singlet at 12.7 ppm, as expected for equivalent P^i^Pr_2_ arms.

**Figure 6:**
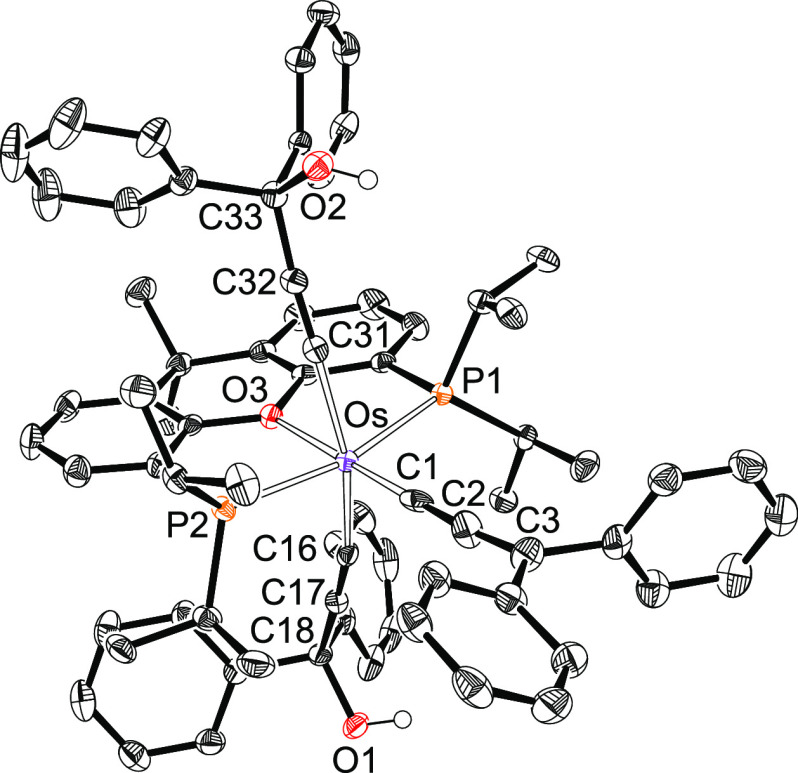
Molecular diagram of complex **7** (ellipsoids shown at 50% probability). All hydrogen atoms (except OH) are omitted for clarity. Selected bond distances (Å) and angles (deg): Os–P(1) = 2.3254(8), Os–P(2) = 2.3337(8), Os–O(3) = 2.246(2), Os–C(16) = 2.067(3), Os–C(31) = 2.067(3), Os–C(1) = 1.868(4), C(1)–C(2) = 1.265(5), C(2)–C(3) = 1.311(5); P(1)–Os–P(2) = 163.46(3), C(1)–Os–O(3) = 177.63(11), C(16)–Os–C(31) = 157.89(12), Os–C(1)–C(2) = 178.7(3), C(1)–C(2)–C(3) = 174.0(4).

Complex **7** was also characterized by X-ray diffraction analysis. Figure 6 shows a molecular diagram of the species. The geometry around the metal center resembles that of **6**, with the allenylidene group in the position of the hydroxyvinylidene ligand. C_3_-cumulene binds to the osmium atom in a nearly linear fashion [Os–C(1)–C(2) = 178.7(3)° and C(1)–C(2)–C(3) = 174.0(4)°]. Distances throughout the metal-cumulene chain of 1.868(4) Å [Os–C(1)], 1.265(5) Å [C(1)–C(2)], and 1.311(5) Å [C(2)–C(3)] compare well with those previously reported for structurally characterized complexes of this class. In agreement with them, the C(1)–C(2) and C(2)–C(3) values suggest a notable contribution of the canonical form [M]^−^–C≡C–C^+^Ph_2_ to the structure of cumulene.^22b,23b,36^ The C_3_-chain gives rise to three triplets in the ^13^C{^1^H} NMR spectrum, which in benzene-*d*_*6*_ appear at 251.3 (C_α_, ^2^*J*_C–P_ = 10.1 Hz), 247.3 (C_β_, ^3^*J*_C–P_ = 3.7 Hz), and 154.2 (C_γ_, ^4^*J*_C–P_ = 2.1 Hz) ppm. Noticeable resonances of this spectrum are also a triplet (^2^*J*_C–P_ = 11.7 Hz) at 103.8 ppm and a singlet at 75.1 ppm, respectively, corresponding to the C_α_ and C_β_ atoms of the triple bond of the hydroxyalkynyl ligands. In agreement with **6**, the ^31^P{^1^H} spectrum displays a singlet at 13.3 ppm.

**Scheme 5:**
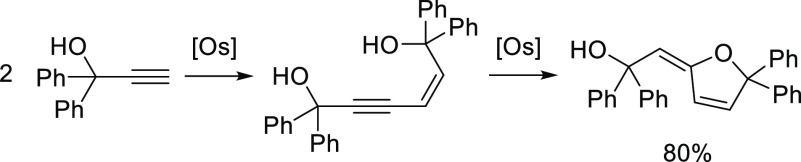
Head-to-Head (*Z*)-Dimerization of 1,1-Diphenyl-2-propyn-1-ol and Formation of the Furanol Derivative

Complex **6** is a triol-functionalized counterpart of complexes Os(C≡CR)_2_(=C=CHR){xant(P^i^Pr_2_)_2_} (R = Ph, ^*t*^Bu). Such vinylidene-osmium(II)-bis(alkynyl) derivatives efficiently catalyze the stereoselective head-to-head (*Z*)-dimerization of terminal alkynes to afford the corresponding enynes (*Z*)-RC≡CCH=CHR.^24a^ In accordance with this, complex **2** promotes the head-to-head (*Z*)-dimerization of 1,1-diphenyl-2-propyn-1-ol via the active species **6**, which is generated in situ. The reactions were performed in a closed system, at 110 and 140 °C, in toluene, using 5 mol % of the osmium precursor. At 110 °C, the dimerization selectively affords (*Z*)-1,1,6,6-tetraphenylhex-2-en-4-yne-1,6-diol in 70% yield after 2 h. At 140 °C, the hydroxy group at 1-position of the enyne adds to the C–C triple bond to produce the furanol derivative (*E*)-2-(5,5-diphenylfuran-2(*5H*)-ylidene)-1,1-diphenylethan-1-ol in 80% yield after 24 h (Scheme 5).

Scheme 5 summarizes a rare example of the tandem-catalyzed reaction,^37^ which has no precedent in the osmium chemistry. The process involves the dimerization of the functionalized alkyne and the subsequent cycloisomerization of the resulting enynediol to afford an interesting furanylideneethanol. The formation of the five-membered heterocycle is noteworthy since such a moiety is present as a structural subunit in numerous natural products with a variety of applications in several fields. Metal catalysts for the dimerization of terminal propargylic alcohols are scarce. In contrast to **6**, the majority of them lead to products resulting from a head-to-head (*E*)-coupling^38^ and in some cases, from a head-to-tail dimerization.^39^ The formation of alkylidenebenzocyclobutenyl alcohols has also been observed.^40^ Catalysts for the cycloisomerization of enynols are even more rare,^41^ and as far as we know, have not been applied to diols.

**Scheme 6:**
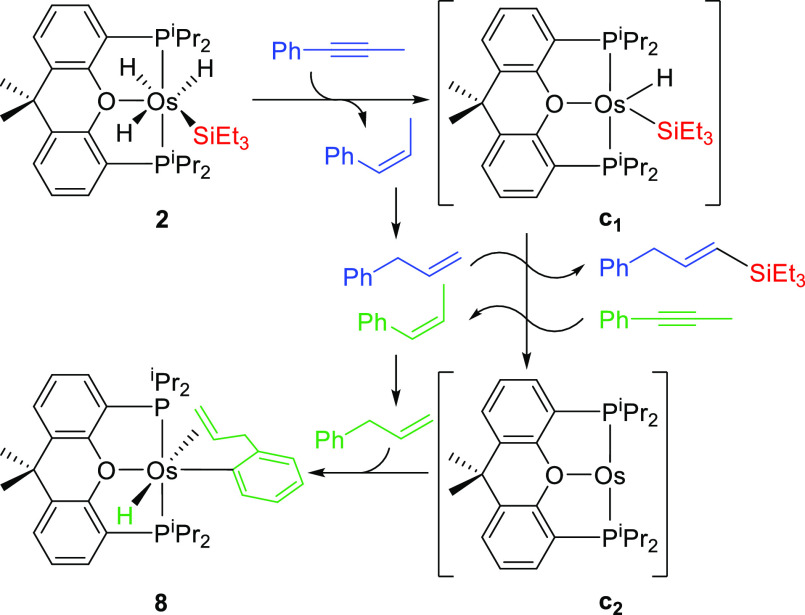
Reaction of OsH_3_(SiEt_3_){κ^3^-*P*,*O*,*P*-[xant(P^i^Pr_2_)_2_]} with 1-Phenyl-1-propyne

Complex **2** also reacts with internal alkynes such as 1-phenyl-1-propyne. Treatment of its solutions in toluene with 2.0 equiv of the hydrocarbon, under reflux, for 16 h leads to OsH{κ^1^-C,η^2^-[C_6_H_4_CH_2_CH=CH_2_]}{κ^3^-*P*,*O*,*P*-[xant(P^i^Pr_2_)_2_]} (**8**) and (*E*)-triethyl(3-phenylprop-1-en-1-yl)silane. The reaction can be rationalized according to Scheme 6. Complex **2** could initially reduce 1.0 equiv of the internal alkyne to afford the unsaturated silyl-osmium(II)-hydride intermediate **c**_**1**_ and 1-pheny-1-propene. Under the reaction conditions, the generated olefin should isomerize from internal to terminal. Thus, the silyl-osmium(II)-hydride species **c**_**1**_ could promote the dehydrogenative silylation of the resulting 3-phenyl-1-propene in the presence of a second equiv of internal alkyne that should act as the hydrogen acceptor. As a result, the silylated olefin at the terminal position, an osmium(0) intermediate **c**_**2**_, and 1-phenyl-1-propene again would be formed. The isomerization of 1-phenyl-1-propene to 3-phenyl-1-propene, followed by the C–C double bond-assisted activation of an *ortho*-CH bond of the phenyl substituent of the latter, promoted by intermediate **c**_**2**_, should finally afford the organometallic product.

**Figure 7:**
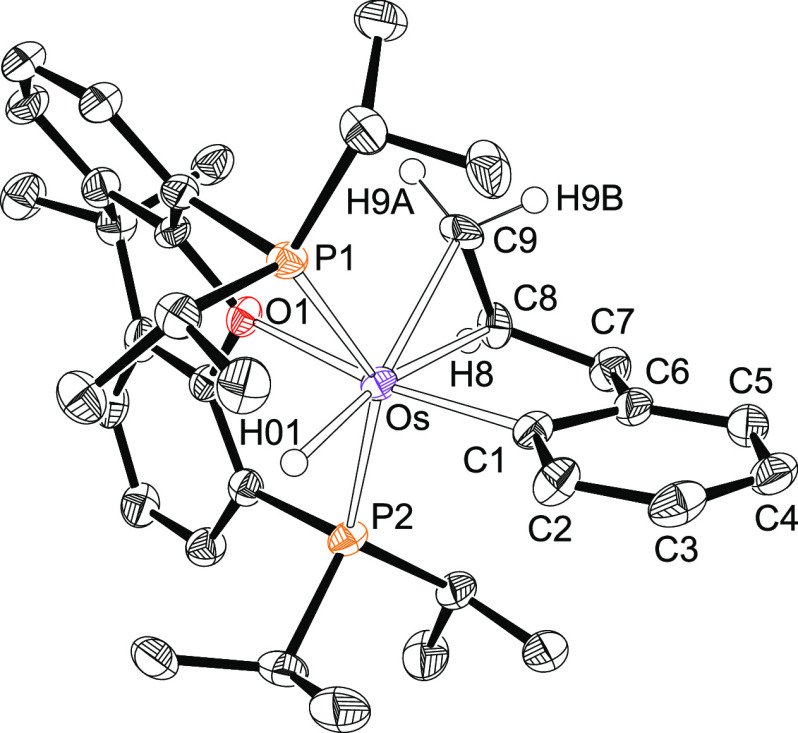
Molecular diagram of complex **8** (ellipsoids shown at 50% probability). All hydrogen atoms are omitted (except the hydride and the olefinic hydrogen atoms) for clarity. Selected bond distances (Å) and angles (deg): Os–P(1) = 2.2926(15), Os–P(2) = 2.2981(16), Os–O(1) = 2.254(4), Os–C(1) = 2.055(6), Os–C(8) = 2.173(6), Os–C(9) = 2.178(6); P(1)–Os–P(2) = 141.44(6), O(1)–Os–C(1) = 168.0(2).

Complex **8** was obtained as a yellow solid in 65% yield and characterized by X-ray diffraction analysis. Figure 7 shows a view of the molecule. The coordination around the osmium atom can be described as a very distorted octahedron. The distortion is consequence of the steric hindrance experienced by the P^i^Pr_2_ arms of the *mer*-disposed ether-diphosphine and the olefinic C(8)–C(9) bond and explains why only one from the two olefins generated in the reaction selectively attaches to the osmium atom, the olefin with less steric hindrance in its C–C double bond. The parallel disposition of the olefinic C(8)–C(9) bond to the P(1)–Os–P(2) axis decreases the phosphine bite angle until 141.44(6)°, a value strongly deviated from the ideal 180°. In agreement with the concerted character of the oxidative addition of the C–H bond, the hydride ligand H(01) and the metalated carbon atom C(1) of the phenyl group are mutually *cis*-disposed. The oxygen atom of the diphosphine lies *trans* to the phenyl group [O(1)–Os–C(1) = 168.0(2)°], whereas the C(8)–C(9) bond situates *trans* to H(01). The C(8)–C(9) bond coordinates to the osmium atom with Os–C(8) and Os–C(9) distances of 2.173(6) and 2.178(6) Å, which are almost identical. The coordination causes a significant elongation of the double bond, as expected for the Chatt–Dewar–Ducanson bonding model. Thus, the C(8)–C(9) bond length of 1.424(9) Å is notably longer than those usually observed for free C–C double bonds of around 1.34 Å.^42^ In accordance with the strong addition of this bond to the metal center, the resonances corresponding to C(8) and C(9) are observed at significant high fields, 41.0 and 36.3 ppm, respectively, in the ^13^C{^1^H} NMR spectrum in benzene-*d*_*6*_. In a consistent manner, the signals due to the associated hydrogen atoms appear at 3.67 [C(8)H], and 2.49 and 1.84 [C(9)H_2_] ppm in the ^1^H NMR spectrum, which displays the hydride resonance at −7.15 ppm as a doublet of doublets with H–P coupling constants of 33.0 and 37.4 Hz. As a result of the asymmetry imposed by the coordination of the olefin, the P^i^Pr_2_ arms of the pincer are inequivalent. Accordingly, the ^31^P{^1^H} NMR spectrum shows an AB spin system centered at 36.2 ppm and defined by Δν = 726 Hz and *J*_A–B_ = 164 Hz.

## Concluding Remarks

This study has revealed that the tetrahydride OsH_4_{κ^3^-*P*,*O*,*P*-[xant(P^i^Pr_2_)_2_]} activates the Si–H bond of tertiary silanes to form silyl-osmium(IV)-trihydride derivatives, in spite of its saturated character. A detailed study of the mechanism of the activation pointed out that the activation is possible because of the hemilabile nature of ether-diphosphine, which dissociates its oxygen atom to permit the Si–H coordination of the silane. The subsequent oxidative addition of the coordinated bond, followed by the reductive elimination of molecular hydrogen, affords the silylated polyhydrides. Kinetics of the addition and the observed primary isotope effect demonstrate that the rupture of the Si–H bond is the rate-determining step of the metal silylation.

These silyl-osmium(IV)-trihydride complexes promote a catalytic tandem process, which converts 1,1-diphenyl-2-propyn-1-ol into an interesting furanylideneethanol via the (*Z*)-enynediol intermediate. The catalysis takes place through the hydroxyvinylidene-osmium(II)-bis(hydroxyalkynyl) derivative Os{C≡CC(OH)Ph_2_}_2_{=C=CHC(OH)Ph_2_}{κ^3^-*P*,*O*,*P*-[xant(P^i^Pr_2_)_2_]}, which has been isolated and fully characterized. The dehydration of the hydroxyvinylidene group of this species yields an allenylidene derivative, which exemplified a novel class of organometallic compounds in the osmium chemistry.

In summary, the Si–H bond activation of silanes promoted by an osmium-tetrahydride, stabilized by a pincer ether-diphosphine, affords silyl-osmium(IV)-trihydride derivatives, which display interesting reactivity towards alkynes, including the catalytic transformation of 1,1-diphenyl-2-propyn-1-ol into (*E*)-2-(5,5-diphenylfuran-2(*5H*)-ylidene)-1,1-diphenylethan-1-ol.

## Experimental Section

### General Information

All reactions were performed with rigorous exclusion of air and moisture at an argon/vacuum manifold using standard Schlenk-tube or glovebox techniques. Alkynes and silanes were purchased from commercial sources and distilled in a Kugelrohr distillation oven prior to use. Complex **1** was prepared according to the published method.^24a^ Instrumental methods, X-ray and theoretical calculation details, and NMR and IR spectra (Figures S2–S48) and kinetic and deuteration experiments (Figures S49–S54) are given in the Supporting Information. Coupling constants *J* and *N* (*N* = *J*_P–H_ + *J*_P′–H_ for ^1^H and *N* = *J*_P–C_ + *J*_P′–C_ for ^13^C{^1^H}) are given in hertz.

### Preparation of OsH_3_(SiEt_3_){κ^3^-*P*,*O*,*P*-[xant(P^i^Pr_2_)_2_]} (**2**)

A solution of **1** (200 mg, 0.31 mmol) in toluene (5 mL) was treated with Et_3_SiH (100 μL, 0.62 mmol). The resulting mixture was heated at reflux for 16 h. After this time, the mixture was concentrated to dryness to afford a yellowish residue. Addition of methanol (5 mL) caused the precipitation of a white solid which was washed with additional methanol (3 × 5 mL) and dried in vacuo. Yield: 182 mg (77%). Anal. Calcd for C_33_H_58_OOsP_2_Si: C, 52.77; H, 7.78. Found: C, 52.48; H, 7.76. HRMS (electrospray, *m*/*z*): calcd for C_27_H_43_OOsP_2_ [M – SiEt_3_]^+^, 637.2416; found, 637.2398. IR (cm^–1^) ν(Os–H): 1841 (m). ^1^H NMR (400.13 MHz, toluene-*d*_8_, 298 K): δ 7.14 (m, 2H, CH-arom, POP), 6.85 (m, 2H, CH-arom, POP), 6.82 (m, 2H, CH-arom, POP), 2.47 (m, 2H, PC*H*(CH_3_)_2_), 2.34 (m, 2H, PC*H*(CH_3_)_2_), 1.34 (t, ^3^*J*_H–H_ = 10.4, 9H, Si(CH_2_C*H*_3_)_3_), 1.31 (s, 3H, C(C*H*_3_)_2_), 1.27 (dvt, ^3^*J*_H–H_ = 7.3, *N* = 16.9, 6H, PCH(C*H*_3_)_2_), 1.08 (dvt, ^3^*J*_H–H_ = 5.6, *N* = 11.8, 6H, PCH(C*H*_3_)_2_), 1.05–0.97 (m, 18H, Si(C*H*_2_CH_3_)_3_ + PCH(C*H*_3_)_2_), 0.93 (s, 3H, C(C*H*_3_)_2_), −1.72 (tt, ^2^*J*_H–H_ = 3.3, ^2^*J*_H–P_ = 9.6, 1H, OsH), −12.26 (vbr, 2H, OsH). ^1^H NMR (400.13 MHz, toluene-*d*_8_, 223 K, high-field region): δ −1.76 (m, 1H, OsH), −4.01 (m, 1H, OsH), −20.21 (m, 1H, OsH). ^13^C{^1^H}-APT NMR (100.62 MHz, toluene-*d*_8_, 298 K): δ 161.7 (vt, *N* = 13.0, C-arom, POP), 133.7 (vt, *N* = 4.9, C-arom, POP), 129.1 (s, CH-arom, POP), 129.0 (s, CH-arom, POP), 127.2 (vt, *N* = 27.0, C-arom, POP), 124.8 (s, CH-arom, POP), 34.7 (s, *C*(CH_3_)_2_), 32.1 (s, C(*C*H_3_)_2_), 27.8 (vt, *N* = 19.9, P*C*H(CH_3_)_2_), 23.8 (vt, *N* = 32.7, P*C*H(CH_3_)_2_), 22.1 (s, C(*C*H_3_)_2_), 21.0 (s, PCH(*C*H_3_)_2_), 19.2 (vt, *N* = 9.8, PCH(*C*H_3_)_2_), 19.0 (vt, *N* = 3.8, PCH(*C*H_3_)_2_), 15.8 (vt, *N* = 4.3, PCH(*C*H_3_)_2_), 14.9 (s, Si(*C*H_2_CH_3_)_3_), 9.7 (s, Si(CH_2_*C*H_3_)_3_). ^31^P{^1^H} NMR (161.98 MHz, toluene-*d*_8_, 298 K): δ 50.0 (s). ^29^Si{^1^H} NMR (59.63 MHz, C_6_D_6_, 298 K): δ 2.0 (t, ^2^*J*_Si–P_ = 4.6). *T*_1(min)_ (ms, OsH, 300.13 MHz, toluene-*d*_8_, 248 K) 231 ± 3 (−20.21 ppm).

### Preparation of OsH_3_(SiPh_3_){κ^3^-*P*,*O*,*P*-[xant(P^i^Pr_2_)_2_]} (**3**)

A solution of **1** (200 mg, 0.31 mmol) in toluene (5 mL) was treated with HSiPh_3_ (164 mg, 0.62 mmol). The resulting mixture was heated at reflux for 16 h. After this time, the mixture was concentrated to dryness to afford a yellowish residue. Addition of methanol (5 mL) caused the precipitation of a white solid which was washed with additional methanol (3 × 5 mL) and dried in vacuo. Yield: 275 mg (97%). Colorless single crystals suitable for X-ray diffraction analysis were obtained from a saturated solution of **3** in benzene at room temperature. Anal. Calcd for C_45_H_58_OOsP_2_Si: C, 60.38; H, 6.53. Found: C, 60.77; H, 6.51. HRMS (electrospray, *m*/*z*): calcd for C_27_H_43_OOsP_2_ [M – SiPh_3_]^+^, 637.2398; found, 637.2371. IR (cm^–1^) ν(Os–H): 1789 (m). ^1^H NMR (400.13 MHz, toluene-*d*_8_, 298 K): δ 8.15 (d, ^3^*J*_H–H_ = 7.4, 6H, *o*-Ph), 7.23 (dd, ^3^*J*_H–H_ = 7.4, ^3^*J*_H–H_ = 7.4, 6H, *m*-Ph), 7.12 (t, ^3^*J*_H–H_ = 7.4, 3H, *p*-Ph), 7.03 (m, 2H, CH-arom, POP), 6.89 (d, ^3^*J*_H–H_ = 7.4, 2H, CH-arom, POP), 6.82 (t, ^3^*J*_H–H_ = 7.5, 2H, CH-arom, POP), 2.21 (m, 2H, PC*H*(CH_3_)_2_), 1.38 (s, 3H, C(CH_3_)_2_), 1.13 (dvt, ^3^*J*_H–H_ = 7.1, *N* = 15.2, 6H, PCH(C*H*_3_)_2_), 1.04 (s, 3H, C(CH_3_)_2_), 0.98 (dvt, ^3^*J*_H–H_ = 7.4, *N* = 16.9, 6H, PCH(C*H*_3_)_2_), 0.77–0.66 (m, 12H, PCH(C*H*_3_)_2_), 0.48 (m, 2H, PC*H*(CH_3_)_2_), −0.12 (t, ^2^*J*_H–P_ = 10.3, 1H, OsH), −10.50 (br, 2H, OsH). ^1^H NMR (400.13 MHz, toluene-*d*_8_, 203 K, high-field region): δ −0.14 (t, ^3^*J*_H–H_ = 9.1, 1H, OsH), −3.73 (br, 1H, OsH), −17.08 (br, 1H, OsH). ^13^C{^1^H}-APT NMR (100.62 MHz, toluene-*d*_8_, 298 K): δ 162.0 (m, C-arom, POP), 149.0 (s, C-ipso, Ph), 138.3 (s, CH, Ph), 134.1 (s, C-arom, POP), 129.2 (s, CH-arom, POP), 128.4 (s, CH-arom, POP), 127.1 (s, CH-arom, POP), 127.0 (m, C-arom, POP), 126.4 (s, CH, Ph), 125.0 (s, CH, Ph), 34.8 (s, *C*(CH_3_)_2_), 31.7 (s, C(*C*H_3_)_2_), 23.0 (vt, *N* = 31.9, P*C*H(CH_3_)_2_), 22.8 (vt, *N* = 24.4, P*C*H(CH_3_)_2_), 22.2 (s, C(*C*H_3_)_2_), 21.2 (s, PCH(*C*H_3_)_2_), 19.6 (s, PCH(*C*H_3_)_2_), 18.5 (m, PCH(*C*H_3_)_2_), 14.7 (s, PCH(*C*H_3_)_2_). ^31^P{^1^H} NMR (161.98 MHz, toluene-*d*_8_, 298 K) δ 47.4 (s). ^29^Si{^1^H} NMR (59.63 MHz, C_6_D_6_, 298 K): δ 1.5 (t, ^2^*J*_Si–P_ = 6.0). *T*_1(min)_ (ms, OsH, 300.13 MHz, toluene-*d*_8_, 230 K) 251 ± 3 (−17.08 ppm).

### Preparation of OsH_3_{SiMe(OSiMe_3_)_2_}{κ^3^-*P*,*O*,*P*-[xant(P^i^Pr_2_)_2_]} (**4**)

A solution of **1** (200 mg, 0.31 mmol) in toluene (5 mL) was treated with HSiMe(OSiMe_3_)_2_ (171 μL, 0.63 mmol). The resulting mixture was heated at reflux for 16 h. After this time, the mixture was concentrated to dryness to afford a yellowish residue. Addition of methanol (5 mL) caused the precipitation of a white solid which was washed with additional methanol (3 × 5 mL) and dried in vacuo. Yield: 245 mg (91%). Anal. Calcd for C_34_H_64_O_3_OsP_2_Si_3_: C, 47.63; H, 7.52. Found: C, 47.89; H, 7.38. HRMS (electrospray, *m*/*z*): calcd for C_34_H_63_O_3_OsP_2_Si_3_ [M – H]^+^, 857.3167; found, 857.3180. IR (cm^–1^) ν(Os–H): 1818 (m). ^1^H NMR (400.13 MHz, toluene-*d*_8_, 298 K): δ 7.15 (m, 2H, CH-arom, POP), 6.88 (m, 2H, CH-arom, POP), 6.85 (m, 2H, CH-arom, POP), 2.82 (m, 2H, PC*H*(CH_3_)_2_), 2.37 (m, 2H, PC*H*(CH_3_)_2_), 1.38 (dvt, ^3^*J*_H–H_ = 7.1, *N* = 17.3, 6H, PCH(C*H*_3_)_2_), 1.34 (s, 3H, C(CH_3_)_2_), 1.14 (dvt, ^3^*J*_H–H_ = 5.8, *N* = 12.3, 6H, PCH(C*H*_3_)_2_), 1.08 (dvt, ^3^*J*_H–H_ = 7.2, *N* = 16.2, 6H, PCH(C*H*_3_)_2_), 1.05 (dvt, ^3^*J*_H–H_ = 6.8, *N* = 15.4, 6H, PCH(C*H*_3_)_2_), 0.94 (s, 3H, C(CH_3_)_2_), 0.74 (s, 3H, SiCH_3_), 0.38 (s, 18H, OSi(CH_3_)_3_), −1.34 (tt, ^2^*J*_H–H_ = 3.2, ^2^*J*_H–P_ = 11.1, 1H, OsH), −11.58 (br, 2H, OsH). ^1^H NMR (400.13 MHz, toluene-*d*_8_, 203 K, high-field region): δ −1.53 (t, ^2^*J*_H–P_ = 10.4, 1H, OsH), −4.42 (br, 1H, OsH), −18.85 (br, 1H, OsH). ^13^C{^1^H}-APT NMR (100.62 MHz, toluene-*d*_8_, 298 K): δ 162.2 (vt, *N* = 13.1, C-arom, POP), 134.2 (vt, *N* = 5.3, C-arom, POP), 129.6 (s, CH-arom, POP), 128.8 (s, CH-arom, POP), 127.4 (vt, *N* = 26.2, C-arom, POP), 125.5 (s, CH-arom, POP), 35.2 (s, *C*(CH_3_)_2_), 33.1 (s, C(*C*H_3_)_2_), 27.0 (vt, *N* = 23.4, P*C*H(CH_3_)_2_), 23.5 (vt, *N* = 32.1, P*C*H(CH_3_)_2_), 22.9 (s, C(*C*H_3_)_2_), 21.4 (s, SiCH_3_), 19.7 (m, PCH(*C*H_3_)_2_), 16.2 (m, PCH(*C*H_3_)_2_), 3.8 (s, OSi(CH_3_)_3_). ^31^P{^1^H} NMR (161.98 MHz, toluene-*d*_8_, 298 K): δ 53.8 (s). ^29^Si{^1^H} NMR (59.63 MHz, toluene-*d*_8_, 298 K): δ −8.5 (s, OSi(CH_3_)_3_), −19.8 (t, ^2^*J*_Si–P_ = 5.4, SiCH_3_). *T*_1(min)_ (ms, OsH, 300.13 MHz, toluene-*d*_8_, 223 K) 288 ± 3 (−18.85 ppm).

### Reaction of OsH_4_{κ^3^-*P*,*O*,*P*-[xant(P^i^Pr_2_)_2_]} (**1**) with (Me_3_SiO)_2_MeSiD

(Me_3_SiO)_2_MeSiD (9 μL, 0.032 mmol) was added to a solution of **1** (10 mg, 0.016 mmol) in 0.5 mL of toluene. The mixture was heated at 140 °C for 18 h. After that, the solvent was removed under vacuo. The ^1^H NMR (300.13 MHz, toluene-*d*_8_, 203 K) data were identical to that reported for **4,** except for the intensity of signals at δ −4.42 (OsH) and −18.85 (OsH), meaning a 50% incorporation of deuterium at hydride positions.

### Preparation of OsH_4_{κ^2^-*P*,*P*-[xant(P^i^Pr_2_)_2_]}(P^i^Pr_3_) (**5**)

A suspension of **1** (200 mg, 0.31 mmol) in methanol (5 mL) was treated with P^i^Pr_3_ (60 μL, 0.31 mmol). The resulting mixture was stirred at room temperature for 2 days. After this time, a white solid was separated, washed with methanol (3 × 5 mL), and dried under vacuum. Yield: 196 mg (78%). Colorless single crystals suitable for X-ray diffraction analysis were obtained by slow diffusion of pentane into a saturated solution of **5** in benzene at room temperature. Anal. Calcd for C_36_H_65_OOsP_3_: C, 54.25; H, 8.22. Found: C, 54.35; H, 8.39. HRMS (electrospray, *m*/*z*): calcd for C_36_H_62_OOsP_3_ [M – H_2_ – H]^+^: 795.3625; found, 795.3614. IR (cm^–1^) ν(Os–H): 2056 (m), ν(Os–H) 2007 (m), ν(Os–H) 1955 (m). ^1^H NMR (300.13 MHz, C_6_D_6_, 298 K): δ 7.18 (m, 2H, CH-arom, POP), 7.11 (m, 2H, CH-arom, POP), 6.99 (m, 2H, CH-arom, POP), 2.55 (m, 4H, PC*H*(CH_3_)_2_, POP), 1.82 (m, 3H, PC*H*(CH_3_)_2_, P^i^Pr_3_), 1.51 (m, 12H, PCH(C*H*_3_)_2_, POP), 1.42 (s, 6H, C(CH_3_)_2_), 1.24 (m, 12H, PCH(C*H*_3_)_2_, POP), 0.93 (dd, ^3^*J*_H–H_ = 7.0, ^3^*J*_H–P_ = 12.5, 18H, PCH(C*H*_3_)_2_), −10.87 (br, 4H, OsH). ^1^H NMR (400.13 MHz, toluene-*d*_8_, 235 K, high-field region): δ −9.99 (br, 2H, OsH), −11.90 (br, 2H, OsH). ^13^C{^1^H}-APT NMR (75.47 MHz, C_6_D_6_, 298 K): δ 156.9 (d, ^2^*J*_C–P_ = 6.7, C-arom, POP), 136.0 (d, ^3^*J*_C–P_ = 2.6, C-arom, POP), 134.2 (d, ^1^*J*_C–P_ = 27.0, C-arom, POP), 128.1 (s, CH-arom, POP), 123.8 (s, CH-arom, POP), 121.8 (d, ^3^*J*_C–P_ = 4.1, CH-arom, POP), 36.4 (s, *C*(CH_3_)_2_), 31.1 (d, ^1^*J*_C–P_ = 25.1, P*C*H(CH_3_)_2_, P^i^Pr_3_), 20.6 (br, PCH(*C*H_3_)_2_, POP), 20.4 (s, PCH(*C*H_3_)_2_, P^i^Pr_3_), 19.3 (br, PCH(*C*H_3_)_2_, POP), (P*C*H(CH_3_)_2_, POP carbon atoms are not observed at this temperature). ^31^P{^1^H} NMR (121.49 MHz, C_6_D_6_, 298 K): δ 44.6 (t, ^2^*J*_P–P_ = 66.2, P^i^Pr_3_), 8.4 (br, POP). ^31^P{^1^H} NMR (161.98 MHz, toluene-*d*_8_, 235 K): δ 44.1 (d, ^2^*J*_P–P_ = 129.8, P^i^Pr_3_), 14.7 (d, ^2^*J*_P–P_ = 129.6, POP), 2.3 (s, POP). *T*_1(min)_ (ms, OsH, 300 MHz, toluene-*d*_8_, 253 K) 162 ± 3 (−11.90 ppm).

### Preparation of Os{C≡CC(OH)Ph_2_}_2_{=C=CHC(OH)Ph_2_}{κ^3^-*P*,*O*,*P*-[xant(P^i^Pr_2_)_2_]} (**6**)

A solution of **2** (200 mg, 0.27 mmol) in toluene (3 mL) was treated with 1,1-diphenyl-2-propyn-1-ol (222 mg, 1.07 mmol). The mixture was heated at 80 °C for 2 h in a Schlenk tube provided with a Teflon closure. The resulting solution was filtered and concentrated to dryness. Addition of pentane (5 mL) afforded a brown precipitate which was washed with pentane (3 × 3 mL) and dried in vacuum. Yield: 174 mg (52%). Brown single crystals suitable for X-ray diffraction analysis were obtained from a saturated methanol solution of **6** at −18 °C. Anal. Calcd for C_72_H_74_O_4_OsP_2_: C, 68.88; H, 5.94. Found: C, 69.05; H, 5.97. HRMS (electrospray, *m*/*z*): calcd for C_72_H_75_O_4_OsP_2_ [M + H]^+^, 1257.4758; found, 1257.4733. IR (cm^–1^) ν(O–H): 3395 (w), ν(C≡C): 2091 (w), ν(C=C): 1655 (m). ^1^H NMR (400.13 MHz, C_6_D_6_, 298 K): δ 7.72 (d, ^3^*J*_H–H_ = 8.3, 4H, CH-arom), 7.46–7.40 (m, 8H, CH-arom), 7.20 (m, 6H, CH-arom), 7.09 (m, 2H, CH-arom), 6.96–6.91 (m, 12H, CH-arom), 6.84 (m, 4H, CH-arom), 4.61 (s, 1H, (=C=CH–C(O*H*)Ph_2_), 2.96 (m, 4H, PC*H*(CH_3_)_2_), 2.62 (s, 2H, −C≡C–C(OH)Ph_2_), 2.45 (t, ^4^*J*_H–P_ = 2.7, 1H, Os=C=CH), 1.35 (s, 6H, C(C*H*_3_)_2_), 1.26 (dvt, ^3^*J*_H–H_ = 7.4, *N* = 16.1, 12H, PCH(*CH*_3_)_2_), 1.18 (dvt, ^3^*J*_H–H_ = 7.1, *N* = 14.8, 12H, PCH(*CH*_3_)_2_). ^13^C{^1^H}-APT NMR (100.64 MHz, C_6_D_6_, 298 K): δ 291.6 (t, ^2^*J*_C–P_ = 9.1, Os=C), 156.2 (vt, *N* = 11.3, C-arom, POP), 152.1 (s, Os=C=CH–*C*(OH)Ph_2_), 148.0 (s, C-ipso, Ph), 133.2 (s, CH-arom, POP), 132.3 (vt, *N* = 5.1, C-arom, POP), 129.4 (s, CH-arom, POP), 129.0 (s, CH-arom), 128.5 (vt, *N* = 32.0, C-arom, POP), 127.9 (s, C-arom), 127.8 (s, CH-arom), 127.0 (s, CH-arom), 126.5 (s, CH-arom), 126.4 (s, CH-arom), 126.2 (s, CH-arom), 124.9 (vt, *N* = 5.5, CH-arom, POP), 120.9 (s, C-arom), 111.5 (t, ^3^*J*_C–P_ = 3.8, =C=*C*H–C(OH)Ph_2_), 102.0 (t, ^2^*J*_C–P_ = 11.8, Os–*C*≡C), 75.5 (s, Os–C≡*C*), 71.6 (s, =C=CH–*C*(OH)Ph_2_), 34.5 (s, *C*(CH_3_)_2_), 32.0 (s, C(*C*H_3_)_2_), 27.4 (vt, *N* = 26.8, P*C*H(CH_3_)_2_), 21.8 (s, PCH(*C*H_3_)_2_), 19.8 (s, PCH(*C*H_3_)_2_). ^31^P{^1^H} NMR (161.98 MHz, C_6_D_6_, 298 K): δ 12.7 (s).

### Preparation of Os{C≡CC(OH)Ph_2_}_2_{=C=C=CPh_2_}{κ^3^-*P*,*O*,*P*-[xant(P^i^Pr_2_)_2_]} (**7**)

A suspension of **6** (150 mg, 0.12 mmol) in methanol (5 mL) was stirred at room temperature for 4 h. During this time, a green solid precipitated in the media. This solid was washed with additional methanol (3 × 3 mL) and dried in vacuo. Yield: 125 mg (85%). Green single crystals suitable for X-ray diffraction analysis were obtained from a saturated methanol solution of **7** at −18 °C. Anal. Calcd for C_72_H_72_O_3_OsP_2_: C, 69.88; H, 5.86. Found: C, 69.48; H, 6.23. HRMS (electrospray, *m*/*z*): calcd for C_72_H_73_O_3_OsP_2_ [M + H]^+^, 1239.4652; found, 1239.4627. IR (cm^–1^) ν(O–H): 3442 (w), ν(C≡C): 2083 (w), ν(Os=*C*=*C*): 1888 (m). ^1^H NMR (400.13 MHz, C_6_D_6_, 298 K): δ 7.83 (d, ^3^*J*_H–H_ = 7.3, 4H, CH-arom), 7.38 (d, ^3^*J*_H–H_ = 6.7, 8H, CH-arom), 7.24 (m, 2H, CH-arom), 7.17 (m, 2H, CH-arom), 7.05 (t, ^3^*J*_H–H_ = 7.7, 4H, CH-arom), 6.93–6.82 (m, 16H, CH-arom), 3.01 (m, 4H, PC*H*(CH_3_)_2_), 2.32 (s, 2H, OH), 1.45 (s, 6H, C(C*H*_3_)_2_), 1.39 (dvt, ^3^*J*_H–H_ = 7.6, *N* = 15.8, 12H, PCH(*CH*_3_)_2_), 1.22 (dvt, ^3^*J*_H–H_ = 7.0, *N* = 14.2, 12H, PCH(*CH*_3_)_2_). ^13^C{^1^H}-APT NMR (100.64 MHz, C_6_D_6_, 298 K): δ 251.3 (t, ^3^*J*_C–P_ = 10.1, Os=C=*C*), 247.3 (t, ^2^*J*_C–P_ = 3.7, Os=C), 155.8 (vt, *N* = 11.5, C-arom, POP), 154.2 (t, ^4^*J*_C–P_ = 2.1, Os=C=C=*C*), 148.8 (s, C-ipso, Ph), 133.8 (s, CH-arom, POP), 131.8 (vt, *N* = 4.8, C-arom, POP), 129.5 (vt, *N* = 30.8, C-arom, POP), 129.4 (s, CH-arom, POP), 129.0 (s, CH-arom), 128.4 (s, C-arom), 127.7 (s, CH-arom), 127.0 (s, CH-arom), 126.9 (s, CH-arom), 126.8 (s, CH-arom), 126.3 (s, CH-arom), 125.4 (t, ^4^*J*_C–P_ = 1.2, C(OH)Ph_2_), 124.6 (vt, *N* = 5.1, CH-arom, POP), 103.8 (t, ^2^*J*_C–P_ = 11.7, Os–*C*≡C), 75.1 (s, Os–C≡*C*), 34.4 (s, *C*(CH_3_)_2_), 33.9 (s, C(*C*H_3_)_2_), 26.9 (vt, *N* = 26.4, P*C*H(CH_3_)_2_), 22.1 (s, PCH(*C*H_3_)_2_), 19.6 (s, PCH(*C*H_3_)_2_). ^31^P{^1^H} NMR (161.98 MHz, C_6_D_6_, 298 K): δ 13.3 (s).

### Preparation of OsH{κ^1^-C,η^2^-[C_6_H_4_CH_2_CH=CH_2_]}{κ^3^-*P*,*O*,*P*-[xant(P^i^Pr_2_)_2_]} (**8**)

A solution of **2** (100 mg, 0.13 mmol) in toluene (5 mL) was treated with 1-phenyl-1-propyne (33 μL, 0.27 mmol). This mixture was heated at reflux for 16 h. After this time, the solution was concentrated to dryness under vacuum. The crude was dissolved in 1 mL of toluene and purified by flash column chromatography, charged with neutral alumina, and eluted with toluene. The resultant solution was concentrated under vacuum to furnish a yellow solid. Yield: 65 mg (65%). Yellow single crystals suitable for X-ray diffraction analysis were obtained from a saturated methanol solution of **8** at −18 °C. Anal. Calcd for C_36_H_50_OOsP_2_: C, 57.58; H, 6.71. Found: C, 57.67; H, 6.66. HRMS (electrospray, *m*/*z*): calcd for C_36_H_50_OOsP_2_ [M]^+^, 752.2948; found, 752.2970. IR (cm^–1^) ν(Os–H): 2040 (w), ν(C=C): 1569 (w). ^1^H NMR (500.12 MHz, C_6_D_6_, 298 K): δ 7.97 (d, ^3^*J*_H–H_ = 7.3, 1H, CH-arom), 7.22 (ddd, ^3^*J*_H–H_ = 1.9, ^3^*J*_H–P_ = 5.7, ^3^*J*_H–H_ = 7.2, 1H, CH-arom), 7.20–7.13 (m, 3H, CH-arom), 6.92–6.82 (m, 4H, CH-arom), 6.78 (ddd, ^3^*J*_H–H_ = 1.5, ^3^*J*_H–H_ = 7.3, ^3^*J*_H–H_ = 7.3, 1H, CH-arom), 3.84–3.64 (m, 3H, CH_2_=C*H*–C*H*_2_), 2.84 (m, 1H, PC*H*(CH_3_)_2_), 2.78 (m, 1H, PC*H*(CH_3_)_2_), 2.54–2.44 (m, 2H, PC*H*(CH_3_)_2_ + C*H*_2_=CH), 2.31 (m, 1H, PC*H*(CH_3_)_2_), 1.84 (t, ^3^*J*_H–H_ = 6.4, 1H, C*H*_2_=CH), 1.49 (dd, ^3^*J*_H–H_ = 6.8, ^3^*J*_H–P_ = 15.5, 3H, PCH(C*H*_3_)_2_), 1.43 (dd, ^3^*J*_H–H_ = 6.7, ^3^*J*_H–P_ = 16.6, 3H, PCH(C*H*_3_)_2_), 1.30 (s, 3H, C(C*H*_3_)_2_), 1.18 (dd, ^3^*J*_H–H_ = 5.7, ^3^*J*_H–P_ = 13.6, 3H, PCH(C*H*_3_)_2_), 1.13 (dd, ^3^*J*_H–H_ = 7.2, ^3^*J*_H–P_ = 13.2, 3H, PCH(C*H*_3_)_2_), 1.09 (dd, ^3^*J*_H–H_ = 6.8, ^3^*J*_H–P_ = 14.6, 3H, PCH(C*H*_3_)_2_), 1.05 (dd, ^3^*J*_H–H_ = 7.0, ^3^*J*_H–P_ = 14.1, 3H, PCH(C*H*_3_)_2_), 0.86 (s, 3H, C(C*H*_3_)_2_), 0.73 (dd, ^3^*J*_H–H_ = 6.8, ^3^*J*_H–P_ = 14.8, 3H, PCH(C*H*_3_)_2_), −7.15 (dd, ^2^*J*_H–P_ = 33.0, ^2^*J*_H–P_ = 37.4, 1H, OsH). ^13^C{^1^H}-APT NMR (125.77 MHz, C_6_D_6_, 298 K): δ 159.7 (d, ^2^*J*_C–P_ = 9.2, C-arom, POP), 158.7 (dd, ^4^*J*_C–P_ = 1.2, ^2^*J*_C–P_ = 9.5, C-arom, POP), 153.0 (t, ^2^*J*_C–P_ = 1.6, Os–C), 145.5 (s, CH, Ph), 141.6 (dd, ^3^*J*_C–P_ = 4.1, ^3^*J*_C–P_ = 5.9, CH, Ph), 133.7 (d, ^3^*J*_C–P_ = 4.7, C-arom, POP), 133.0 (d, ^3^*J*_C–P_ = 5.1, C-arom, POP), 130.4 (d, ^3^*J*_C–P_ = 29.5, C-arom, POP), 130.0 (s, CH-arom, POP), 129.9 (s, CH-arom, POP), 129.3 (d, ^3^*J*_C–P_ = 30.7, C-arom, POP), 125.5 (m, CH-arom, POP), 125.5 (m, CH-arom, POP), 125.3 (s, CH-arom, POP), 125.2 (s, CH-arom, POP), 125.1 (s, CH-arom, POP), 125.0 (m, CH-arom, POP), 123.1 (s, CH, Ph), 122.3 (s, CH, Ph), 118.8 (s, CH, Ph), 42.3 (t, ^4^*J*_C–P_ = 1.9, CH_2_=CH–*C*H_2_), 41.0 (t, ^3^*J*_C–P_ = 6.2, CH_2_=*C*H–*C*H_2_), 36.3 (dd, ^2^*J*_C–P_ = 5.2, ^2^*J*_C–P_ = 8.5, *C*H_2_=CH), 35.1 (d, ^2^*J*_C–P_ = 2.3, P*C*H(CH_3_)_2_), 35.0 (s, *C*(CH_3_)_2_), 34.9 (m, P*C*H(CH_3_)_2_), 33.6 (s, C(*C*H_3_)_2_), 32.8 (d, ^2^*J*_C–P_ = 2.6, P*C*H(CH_3_)_2_), 32.7 (d, ^2^*J*_C–P_ = 2.6, P*C*H(CH_3_)_2_), 22.9 (s, C(*C*H_3_)_2_), 22.5 (s, PCH(*C*H_3_)_2_), 21.2 (d, ^2^*J*_C–P_ = 6.2, PCH(*C*H_3_)_2_), 20.7 (s, PCH(*C*H_3_)_2_), 20.6 (s, PCH(*C*H_3_)_2_), 20.5 (s, PCH(*C*H_3_)_2_), 20.3 (d, ^2^*J*_C–P_ = 4.3, PCH(*C*H_3_)_2_), 20.1 (d, ^2^*J*_C–P_ = 5.1, PCH(*C*H_3_)_2_), 19.6 (d, ^2^*J*_C–P_ = 3.6, PCH(*C*H_3_)_2_). ^31^P{^1^H} NMR (202.46 MHz, C_6_D_6_, 298 K): δ 36.2 (AB spin system, Δν = 726.1 Hz, *J*_A–B_ = 164.3 Hz).

### Catalytic Dimerization of 1,1-Diphenyl-2-propyn-1-ol to (*Z*)-1,1,6,6-Tetraphenylhex-2-en-4-yne-1,6-diol

An NMR tube was charged with a solution of 1,1-diphenyl-2-propyn-1-ol (55.4 mg, 0.27 mmol) and **2** (10 mg, 0.013 mmol) in toluene-*d*_8_ (0.5 mL). Then, it was placed into a thermostatic bath at 110 °C, and the reaction was monitored by ^1^H NMR spectroscopy, using 1,4-dioxane (2.8 μL, 0.033 mmol) as the internal standard. After 2 h, the ^1^H NMR spectra of the solution showed conversion to (*Z*)-1,1,6,6-tetraphenylhex-2-en-4-yne-1,6-diol in 70% yield. HRMS (electrospray, *m*/*z*): calcd for C_30_H_24_NaO_2_ [M + Na]^+^, 439.1669; found, 439.1659. IR (cm^–1^) ν(O–H): 3529 (w), ν(O–H): 3402 (w). ^1^H NMR (300.13 MHz, CDCl_3_, 298 K): δ 7.80–7.17 (m, 20H, CH-arom), 6.61 (d, ^3^*J*_H–H_ = 11.7, 1H, C*H*=CH), 5.87 (d, ^3^*J*_H–H_ = 11.7, 1H, CH=C*H*), 3.55 (s, OH), 2.69 (s, OH). ^13^C{^1^H}-APT NMR (75.47 MHz, CDCl_3_, 298 K): δ 149.6 (s, CH=CH), 146.2 (s, C-arom), 144.5 (s, C-arom), 130.2 (s, CH-arom), 128.4 (s, CH-arom), 127.8 (s, CH-arom), 127.4 (s, CH-arom), 126.9 (s, CH-arom), 126.1 (s, CH-arom), 109.3 (s, C*H*=CH), 100.9 (s, *C*≡C), 83.4 (s, C≡*C*), 79.7 (s, C(OH)Ph_2_), 74.8 (s, C(OH)Ph_2_).

### Catalytic Dimerization of 1,1-Diphenyl-2-propyn-1-ol to (*E*)-2-(5,5-Diphenylfuran-2(*5H*)-ylidene)-1,1-diphenylethan-1-ol

An NMR tube was charged with a solution of 1,1-diphenyl-2-propyn-1-ol (55.4 mg, 0.27 mmol) and **2** (10 mg, 0.013 mmol) in toluene-*d*_8_ (0.5 mL). Then, it was placed into a thermostatic bath at 140 °C, and the reaction was monitored by ^1^H NMR spectroscopy, using 1,4-dioxane (2.8 μL, 0.033 mmol) as the internal standard. After 24 h, the ^1^H NMR spectra of the solution showed conversion to €-2-(5,5-diphenylfuran-2(*5H*)-ylidene)-1,1-diphenylethan-1-ol in 80% yield. HRMS (electrospray, *m*/*z*): calcd for C_30_H_24_NaO_2_ [M + Na]^+^, 439.1669; found, 439.1675. IR (cm^–1^) ν(O–H): 3534 (w). ^1^H NMR (300.13 MHz, CDCl_3_, 298 K): δ 7.82–6.97 (m, 20H, CH-arom), 6.63 (d, ^3^*J*_H–H_ = 5.8, 1H, C*H*=CH), 6.22 (d, ^3^*J*_H–H_ = 5.8, 1H, CH=C*H*), 5.38 (s, CH=C), 4.35 (s, OH). ^13^C{^1^H}-APT NMR (75.47 MHz, CDCl_3_, 298 K): δ 157.9 (s, CH=*C*), 148.1 (s, C-arom), 142.1 (s, C-arom), 138.3 (s, C*H*=CH), 137.7 (s, C-arom), 128.5 (s, CH-arom), 128.1 (s, CH-arom), 127.9 (s, CH-arom), 126.8 (s, CH-arom), 126.6 (s, CH-arom), 126.4 (s, CH-arom), 124.5 (s, CH=C*H*), 106.6 (s, *C*H=C), 98.4 (s, C(OH)Ph_2_), 78.1 (s, C(OH)Ph_2_).

### Kinetic Experiments

All kinetic experiments were performed in toluene-*d*_8_ solutions contained in NMR tubes. The NMR tubes were charged with complex **1** (10 mg, 0.016 mmol, 1.9 × 10^–2^ M) and triethylsilane (49.5–99 μL, 0.31–0.62 mmol, 0.38–0.76 M) and the final volume was brought to 850 μL using toluene-*d*_8_; a capillary tube filled with a solution of the internal standard (PPh_3_) in toluene-*d*_8_ was also placed in the tube. The reaction was monitored by ^31^P{^1^H} NMR spectroscopy every 15 min (a delay of 8 s was used).

## Supporting Information Available

Experimental details, crystallographic data, theoretical studies, NMR and IR spectra, and deuteration and kinetic experiments (PDF)
Cartesian coordinates of the optimized structures (XYZ)

## Supplementary Material

om2c00201_si_001.pdf

om2c00201_si_002.xyz
