# Supplementary material for: Silyl-Osmium(IV)-Trihydride Complexes Stabilized by a Pincer Ether-Diphosphine: Formation and Reactions with Alkynes
Source: Organometallics. 2022 Jul 18;41(15):2022–34. doi: 10.1021/acs.organomet.2c00201 (PMC9969874; doi:10.1021/acs.organomet.2c00201)
Supplement: Supplementary file 1 — om2c00201_si_001.pdf [file om2c00201_si_001.pdf]

SUPPORTING INFORMATION

Silyl-Osmium(IV)-Trihydride Complexes Stabilized by a Pincer Ether-Diphosphine: Formation and Reactions with Alkynes

Juan C. Babón, Miguel A. Esteruelas, Enrique Oñate, Sonia Paz, and Andrea Vélez*

Departamento de Química Inorgánica – Instituto de Síntesis Química y Catálisis Homogénea (ISQCH) – Centro de Innovación en Química Avanzada (ORFEO-CINQA), Universidad de Zaragoza – CSIC, 50009 Zaragoza, Spain

* e-mail: maester@unizar.es

Contents:

– Experimental Details S2

– Structural Analysis of Complexes **3**, **5**, **6**, **7**, and **8** S3

– Computational Details S5

– Energies of Optimized Structures S6

– DFT-Optimized Structure and Contour Line Diagram of **3** S7

– NMR and IR Spectra S8

– Deuteration Experiments S32

– Kinetic Experiments S33

– References S36

– **Experimental Details**

General Information.

All reactions were carried out with exclusion of air using Schlenk-tube techniques or in a glovebox. Solvents were obtained oxygen- and water-free from an MBraun solvent purification apparatus, or dried and distilled under argon prior to use. ^{1}H, $^{13}C\{^{1}H\}$, and $^{31}P\{^{1}H\}$ NMR spectra were recorded on Bruker 300 ARX, Bruker Avance 300 MHz, Bruker Avance 400 MHz or Bruker Avance 500 MHz instruments. Chemical shifts (expressed in ppm) are referenced to residual solvent peaks (^{1}H, $^{13}C\{^{1}H\}$) and external 85% H_3PO_4 ($^{31}P\{^{1}H\}$) or $SiMe_4$ (^{29}Si). Coupling constants J and N ($N = J_{P\text{–}H} + J_{P'\text{–}H}$ for ^{1}H and $N = J_{P\text{–}C} + J_{P'\text{–}C}$ for $^{13}C\{^{1}H\}$) are given in hertz. High-resolution electrospray mass spectra were acquired using a MicroTOF-Q hybrid quadrupole time-of-flight spectrometer (Bruker Daltonics, Bremen, Germany). Attenuated total reflection infrared spectra (ATR-IR) of solid samples were run on a Perkin-Elmer Spectrum 100 FT-IR spectrometer. Elemental analyses were carried out in a Perkin-Elmer 2400-B Series II CHNS-Analyzer. $OsH_4\{\kappa^3\text{-}P,O,P\text{-}[xant(P^iPr_2)_2]\}$ (**1**) was prepared as reported previously.[1]

– Structural Analysis of Complexes 3, 5, 6, 7, and 8

X-ray data were collected on a APEX CCD (**3**, **7**, **8**), and D8 Venture (**5**, **6**) Bruker diffractometers (Mo radiation, λ = 0.71073 Å). The crystals were cooled with a nitrogen flow from Oxford Cryosystems systems. Data were corrected for absorption by using a multiscan method applied with the SADABS program.[2] The structures were solved by Patterson or direct methods and refined by full-matrix least squares on F^2 with SHELXL2016,[3] including isotropic and subsequently anisotropic displacement parameters. The hydrogen atoms were observed in the last Fourier Maps or calculated, and refined freely or using a restricted riding model. The hydrides were observed in the maps of Fourier, but were refined with a restricted model (d(Os–H = 1.59(1) Å). The POP ligand of **5** was observed disordered and refined with two moieties (A and B) with complementary occupancy factors.

Crystal data for **3** (CCDC 2167977): $C_{45}H_{58}OOsP_2Si$, C_7H_8, M_W 987.27, colourless, irregular block (0.183 x 0.122 x 0.084 mm^3), orthorhombic, space group Pnma, *a*: 27.2288(13) Å, *b*: 14.0713(7) Å, *c*: 12.1290(6) Å, $V = 4647.2(4)$ Å^3, $Z = 4$, $Z' = 0.5$, D_{calc}: 1.411 g cm^{-3}, F(000): 2024, T = 100(2) K, μ 2.875 mm^{-1}. 101622 measured reflections (2θ: 3-57º, ω scans 0.3º), 6153 unique (R_{int} = 0.0622); min./max. transm. Factors 0.710/0.862. Final agreement factors were R^1 = 0.0250 (5251 observed reflections, I > 2σ(I)) and wR^2 = 0.0560; data/restraints/parameters 6153/3/ 296; GoF = 1.070. Largest peak and hole 1.412 (close to osmium atoms) and -1.039 e/ Å^3.

Crystal data for **5** (CCDC 2167976): $C_{36}H_{65}OOsP_3$, M_W 796.99, colourless, irregular block (0.170 x 0.160 x 0.150 mm^3), orthorhombic, space group Pbca, *a*: 17.1118(6) Å, *b*: 14.5413(6) Å, *c*: 29.7901(12) Å, $V = 7412.6(5)$ Å^3, $Z = 8$, $Z' = 1$, D_{calc}: 1.428 g cm^{-3}, F(000): 3280, T = 100(2) K, μ 3.596 mm^{-1}. 112828 measured reflections (2θ: 3-57º, ω scans 0.3º), 11293 unique (R_{int} = 0.0243); min./max. transm. Factors 0.733/0.862. Final

agreement factors were R^1 = 0.0361 (10187 observed reflections, I > 2σ(I)) and wR^2 = 0.0903; data/restraints/parameters 11293/32/357; GoF = 1.001. Largest peak and hole 1.962 (close to osmium atoms) and -3.355 e/ Å^3.

Crystal data for **6** (CCDC 2167978): $C_{72}H_{74}O_4OsP_2$, M_W 1255.45, yellow, needle (0.200 x 0.0.03 x 0.0.03 mm^3), monoclinic, space group $P2_1/c$, *a*: 25.9258(15) Å, *b*: 11.1782(6) Å, *c*: 21.9650(13) Å, *β*: 111.994(2) °, *V* = 5902.3(6) Å^3, *Z* = 4, *Z'* = 1, D_{calc}: 1.413 g cm^{-3}, F(000): 2576, T = 100(2) K, μ 2.265 mm^{-1}. 211972 measured reflections (2θ: 3-57°, ω scans 0.3°), 18029 unique (R_{int} = 0.0765); min./max. transm. Factors 0.773/0.862. Final agreement factors were R^1 = 0.0428 (15810 observed reflections, I > 2σ(I)) and wR^2 = 0.0948; data/restraints/parameters 18029/0/ 728; GoF = 1.154. Largest peak and hole 1.744 (close to osmium atoms) and -4.099 e/ Å^3.

Crystal data for **7** (CCDC 2167974): $C_{72}H_{72}O_3OsP_2$, M_W 1237.43, purple, irregular block (0.251 x 0.182 x 0.093 mm^3), monoclinic, space group $P2_1/n$, *a*: 10.9835(7) Å, *b*: 26.5063(16) Å, *c*: 19.8696(12) Å, *β*: 94.7790(10) °, *V* = 5764.6(6) Å^3, *Z* = 4, *Z'* = 1, D_{calc}: 1.426 g cm^{-3}, F(000): 2536, T = 100(2) K, μ 2.317 mm^{-1}. 66570 measured reflections (2θ: 3-57°, ω scans 0.3°), 13955 unique (R_{int} = 0.0579); min./max. transm. Factors 0.656/0.862. Final agreement factors were R^1 = 0.0339 (10733 observed reflections, I > 2σ(I)) and wR^2 = 0.0747; data/restraints/parameters 13955/0/ 715; GoF = 0.998. Largest peak and hole 1.082 (close to osmium atoms) and -1.499 e/ Å^3.

Crystal data for **8** (CCDC 2167975): $C_{36}H_{50}OOsP_2$, M_W 750.90, colorless, irregular block (0.126 x 0.118 x 0.099 mm^3), monoclinic, space group C2/c, *a*: 31.841(2) Å, *b*: 10.1680(7) Å, *c*: 23.0062(15) Å, *β*: 115.8200(10) °, *V* = 6704.8(8) Å^3, *Z* = 8, *Z'* = 1, D_{calc}: 1.488 g cm^{-3}, F(000): 3040, T = 100(2) K, μ 3.925 mm^{-1}. 34273 measured reflections (2θ: 3-57°, ω scans 0.3°), 8018 unique (R_{int} = 0.0593); min./max. transm. Factors 0.656/0.862. Final agreement factors were R^1 = 0.0452 (6503 observed reflections, I >

$2\sigma(I)$) and $wR^2 = 0.1021$; data/restraints/parameters 8018/1/383; GoF = 1.064. Largest peak and hole 1.470 (close to osmium atoms) and -1.957 e/ Å^3.

– Computational Details

All calculations were performed at the DFT level using the B3LYP functional[4] supplemented with the Grimme's dispersion correction D3[5] as implemented in Gaussian09.[6] Os atoms were described by means of an effective core potential SDD for the inner electron[7] and its associated double-ζ basis set for the outer ones, complemented with a set of f-polarization functions for iridium.[8] The 6-31G** basis set was used for all the other atoms.[9] All minima were verified to have no negative frequencies. The AIM calculations were performed with the AIMAII program.[10]

– Energies of Optimized Structures

Complex **3t**:

Zero-point correction= 0.915374 (Hartree/Particle)

Thermal correction to Energy= 0.967320

Thermal correction to Enthalpy= 0.968264

Thermal correction to Gibbs Free Energy= 0.833256

Sum of electronic and zero-point Energies= -2887.332627

Sum of electronic and thermal Energies= -2887.280681

Sum of electronic and thermal Enthalpies= -2887.279736

Sum of electronic and thermal Free Energies= -2887.414744

Complex **5t**:

Zero-point correction= 0.938181 (Hartree/Particle)

Thermal correction to Energy= 0.988154

Thermal correction to Enthalpy= 0.989098

Thermal correction to Gibbs Free Energy= 0.862868

Sum of electronic and zero-point Energies= -2600.400789

Sum of electronic and thermal Energies= -2600.350815

Sum of electronic and thermal Enthalpies= -2600.349871

Sum of electronic and thermal Free Energies= -2600.476101

– DFT-Optimized Structure and Contour Line Diagram of 3

a)

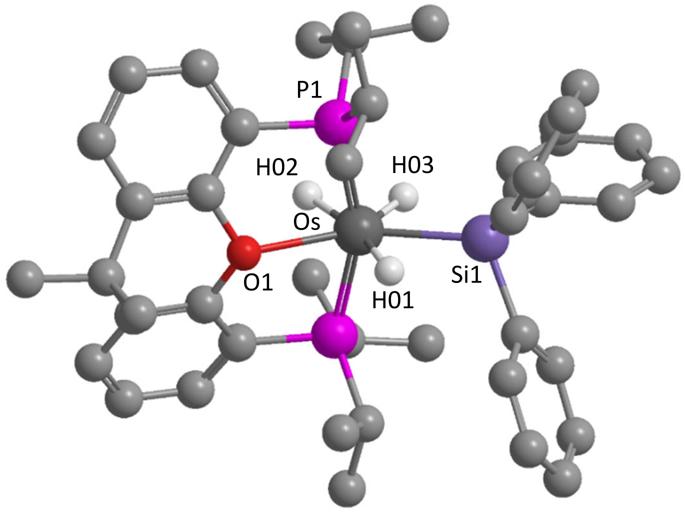

b)

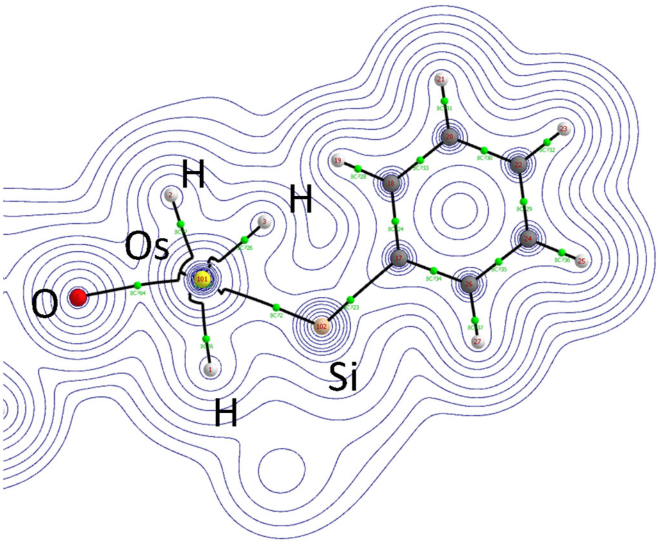

Figure S1. (a) DFT-optimized structure of **3**. All hydrogen atoms (except the hydrides) are omitted for clarity. Selected bond distances (Å) and angles (deg): Os–H(01) = 1.697, Os–H(02) = 1.680, Os–H(03) = 1.584, Os–P(1) = 2.322, Os–O(1) = 2.360, Os–Si(1) = 2.409, P(1)–Os–P(1) = 158.347, O(1)–Os–Si(1) = 150.234. (b) Contour line diagrams $\nabla^2 \rho(r)$ for **3** in the O–Os–Si plane. The solid lines connecting the atomic nuclei are the bond paths, while the small green and red spheres indicate the corresponding BCPs and RCPs, respectively. [Legend: H (white), Si (orange), C (gray), O (red), and Os (yellow)].

– NMR and IR Spectra

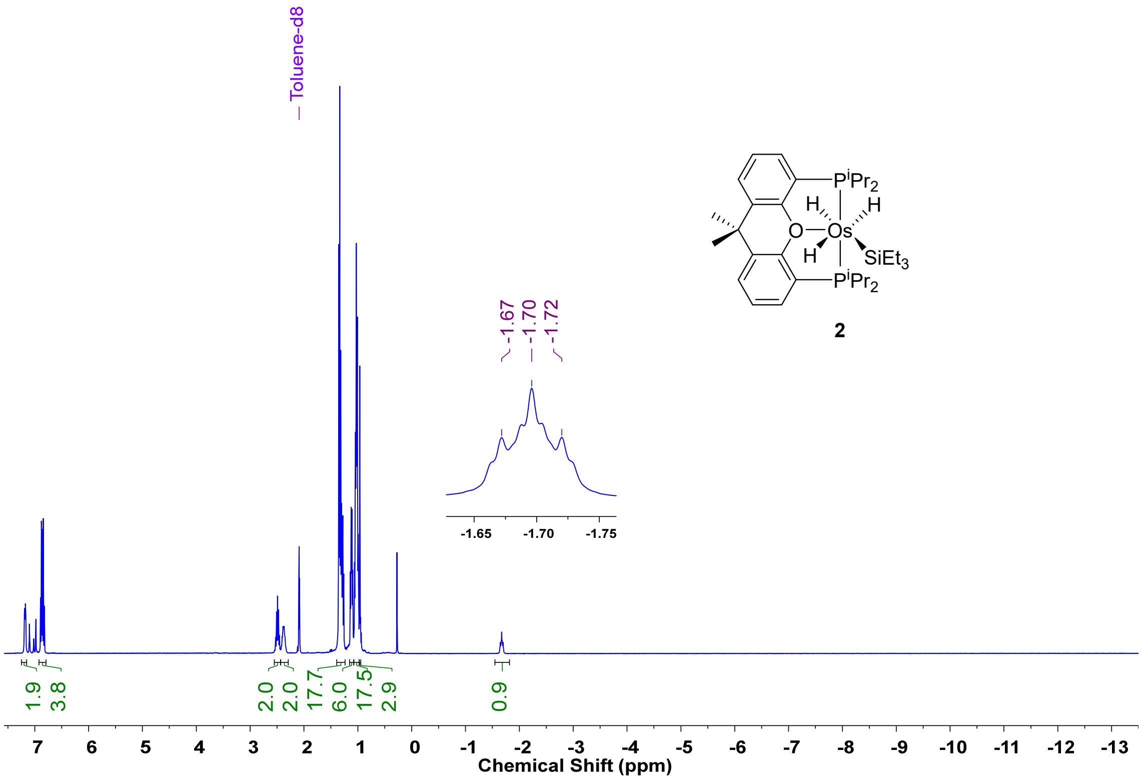

Figure S2. ^{1}H NMR spectrum (400.13 MHz, toluene-d_8, 298 K) of **2**.

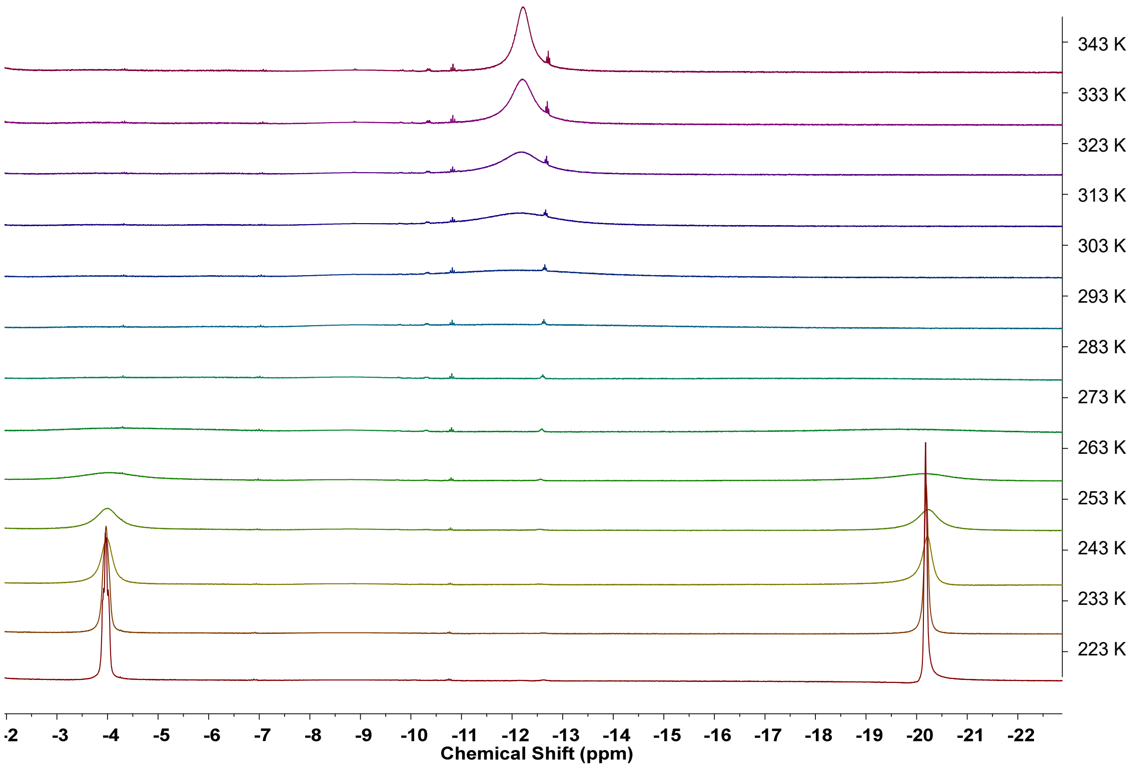

Figure S3. High-field region of ^{1}H NMR spectra (400.13 MHz, toluene-d_8, 343-223 K) of **2**.

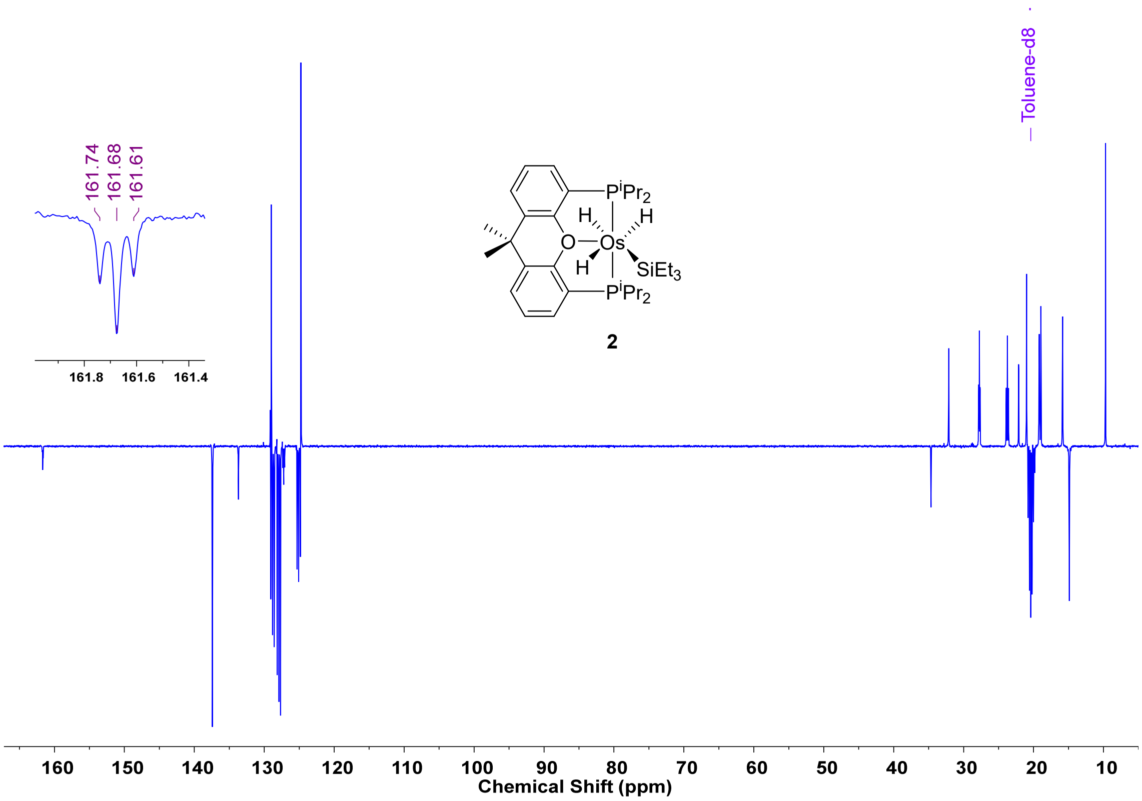

Figure S4. $^{13}C\{^1H\}$-APT NMR spectrum (100.62 MHz, toluene-d_8, 298 K) of **2**.

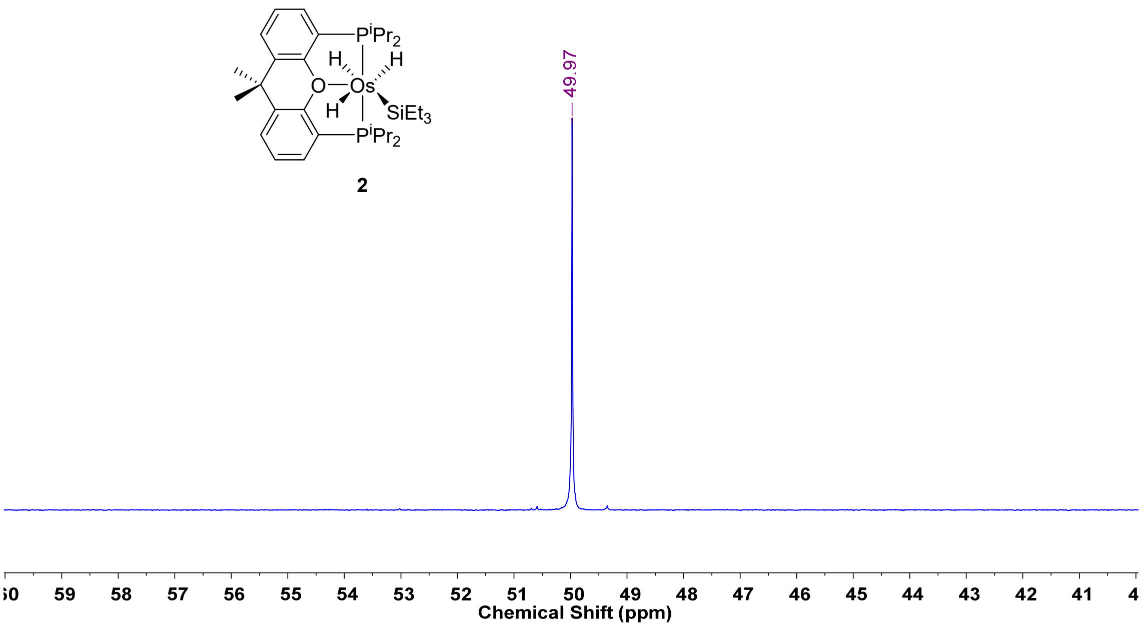

Figure S5. $^{31}P\{^1H\}$ NMR spectrum (161.98 MHz, toluene-d_8, 298 K) of **2**.

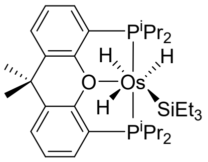

2

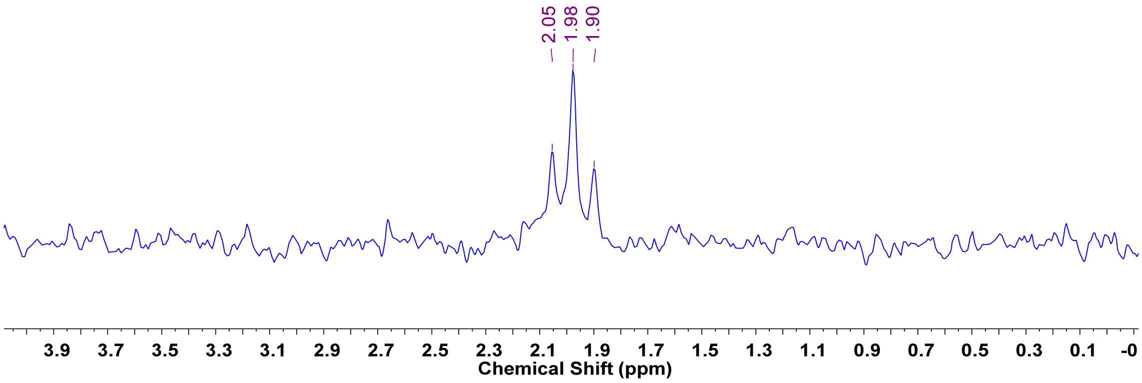

Figure S6. $^{29}Si\{^1H\}$ NMR spectrum (59.63 MHz, C_6D_6, 298 K) of **2**.

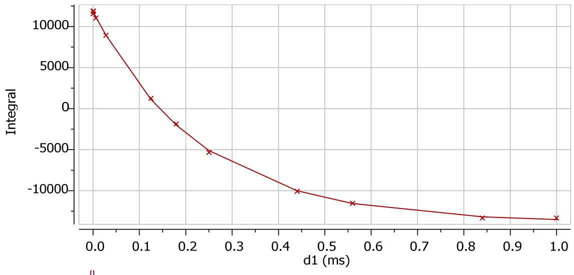

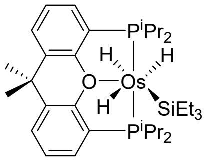

2

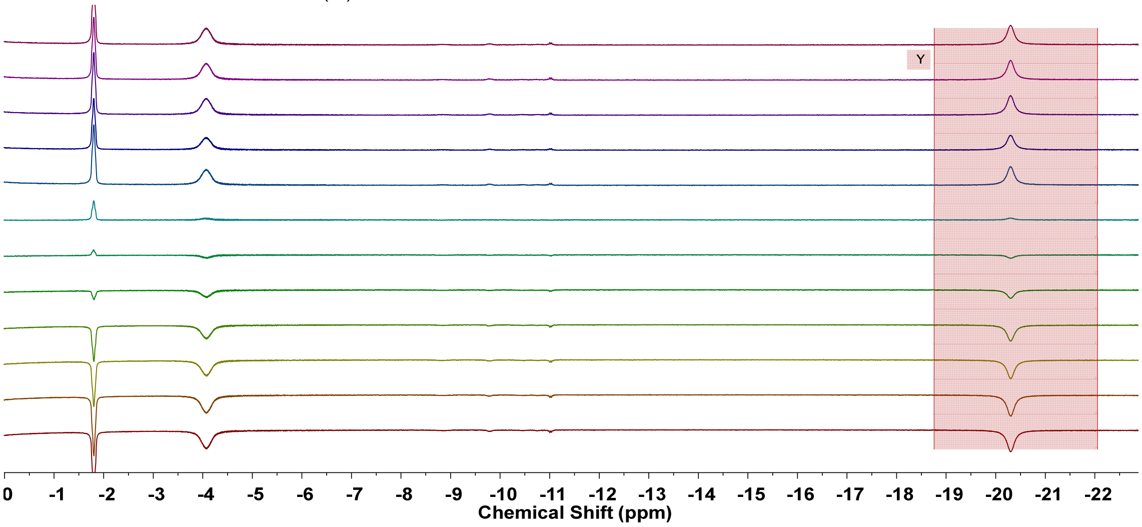

Figure S7. ^1H NMR $T_{1(min)}$ relaxation time measurement (300.13 MHz, toluene-d_8, 248 K, OsH) of **2**.

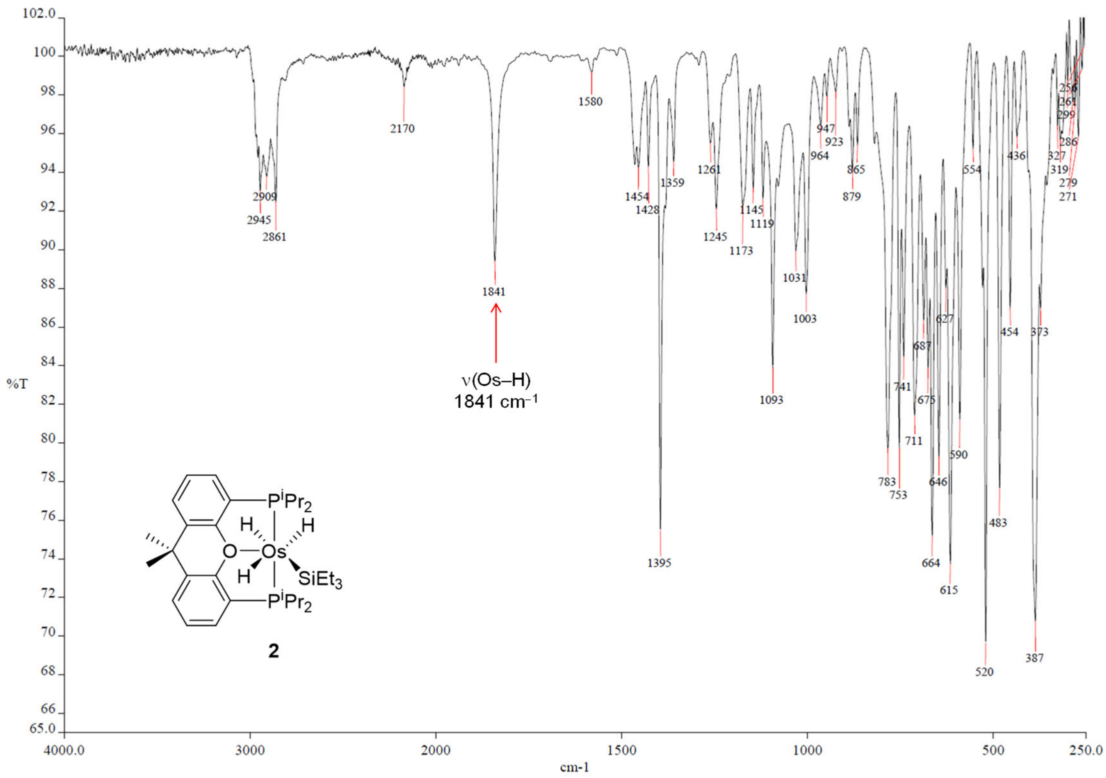

Figure S8. ATR-IR spectrum of **2**.

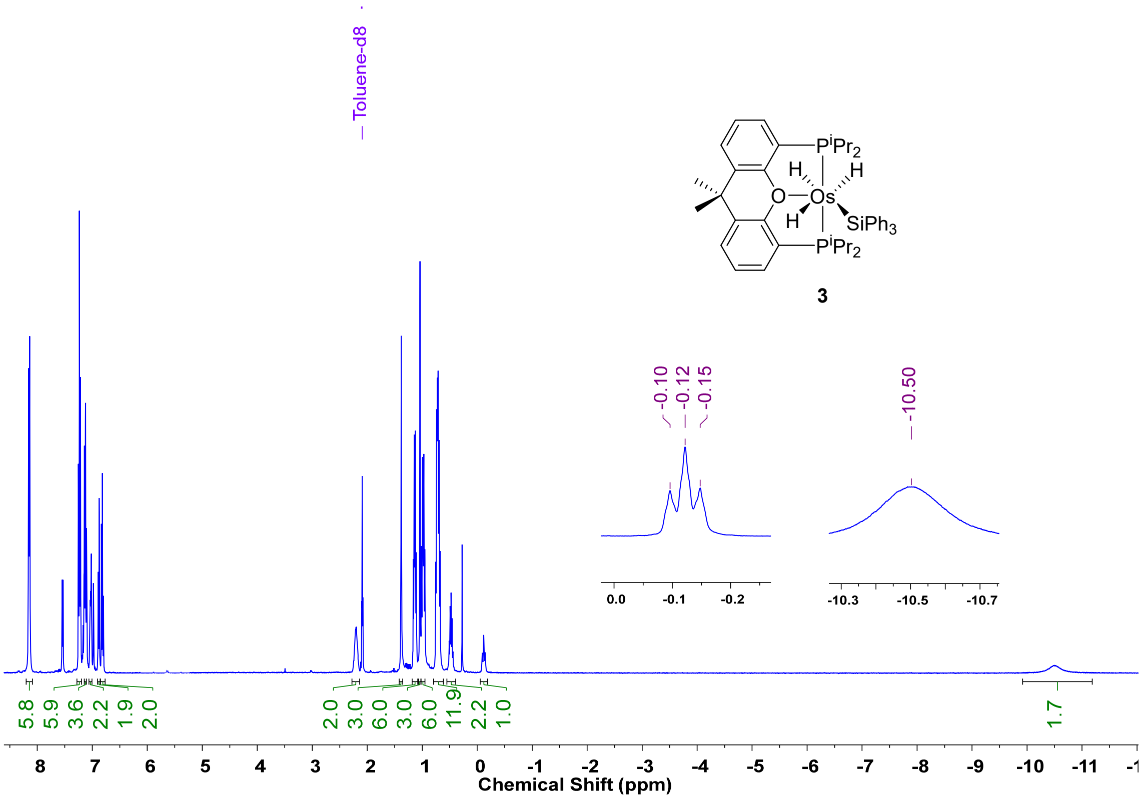

Figure S9. ^{1}H NMR spectrum (400.13 MHz, toluene-d_8, 298 K) of **3**.

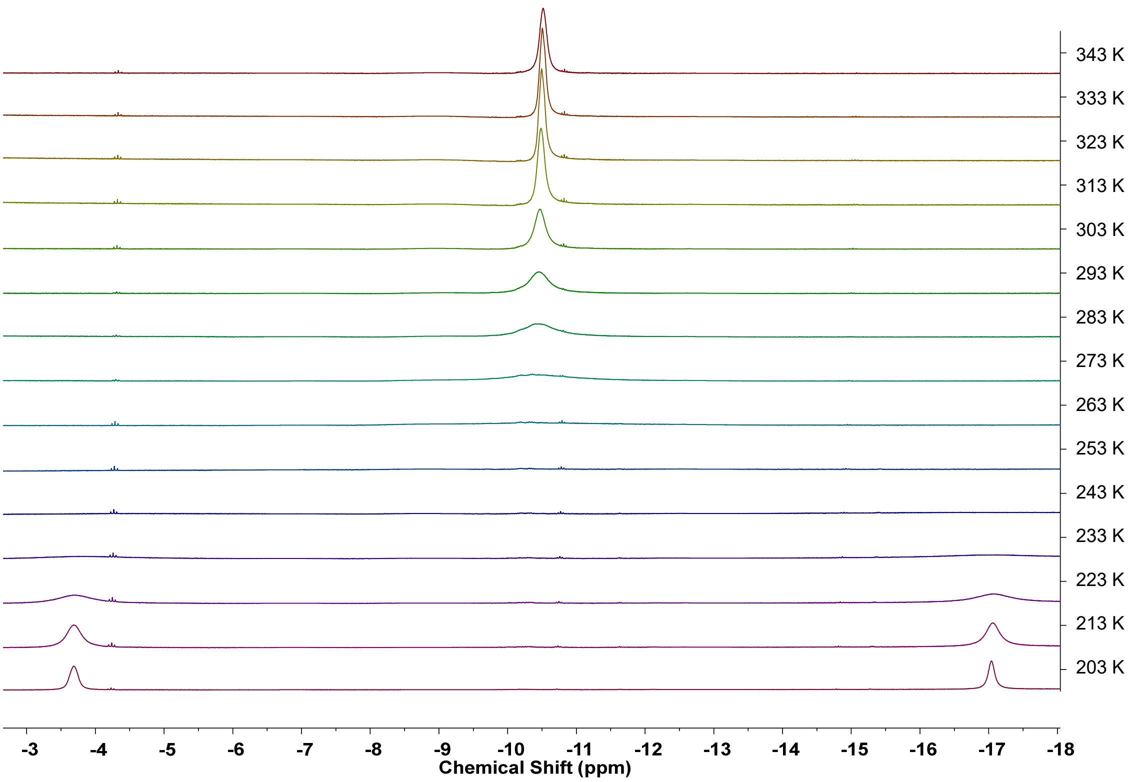

Figure S10. High-field region of ^{1}H NMR spectra (400.13 MHz, toluene-d_8, 343-203 K) of **3**.

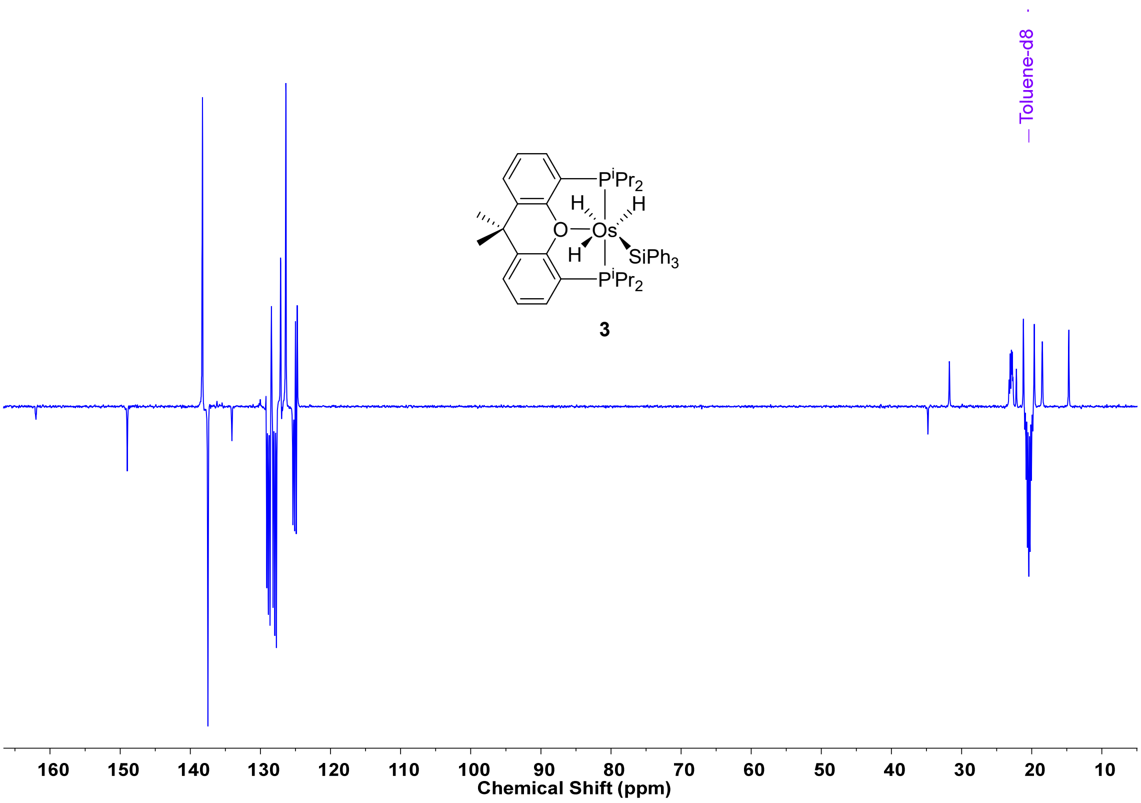

Figure S11. ^{13}C{^{1}H}-APT NMR spectrum (100.62 MHz, toluene-d_8, 298 K) of **3**.

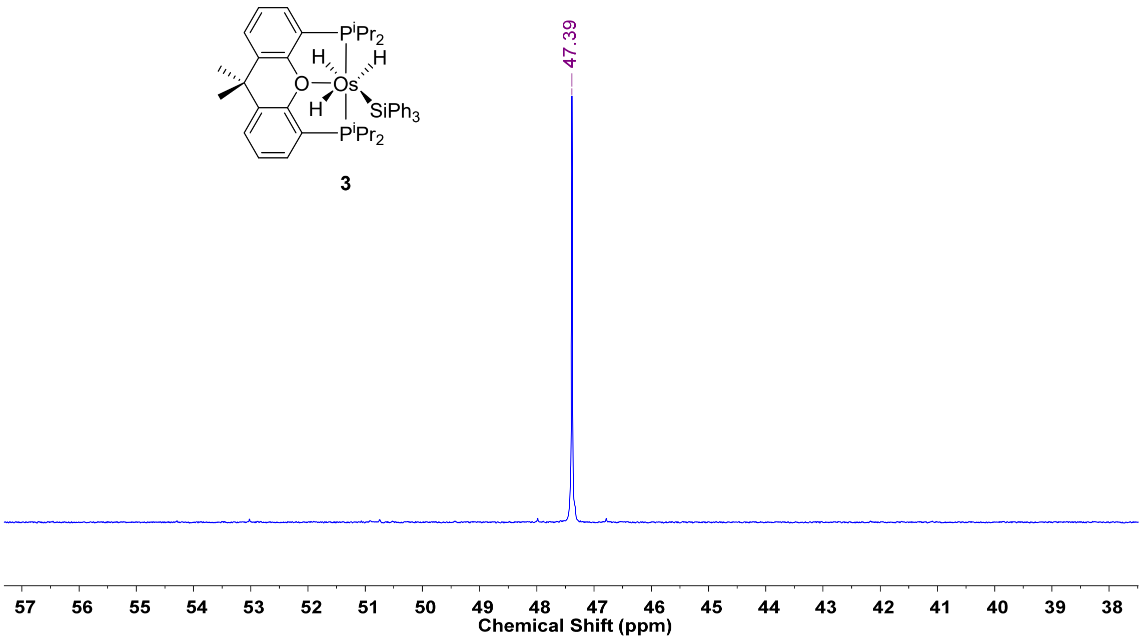

Figure S12. $^{31}P\{^{1}H\}$ NMR spectrum (161.98 MHz, toluene-*d*$_8$, 298 K) of **3**.

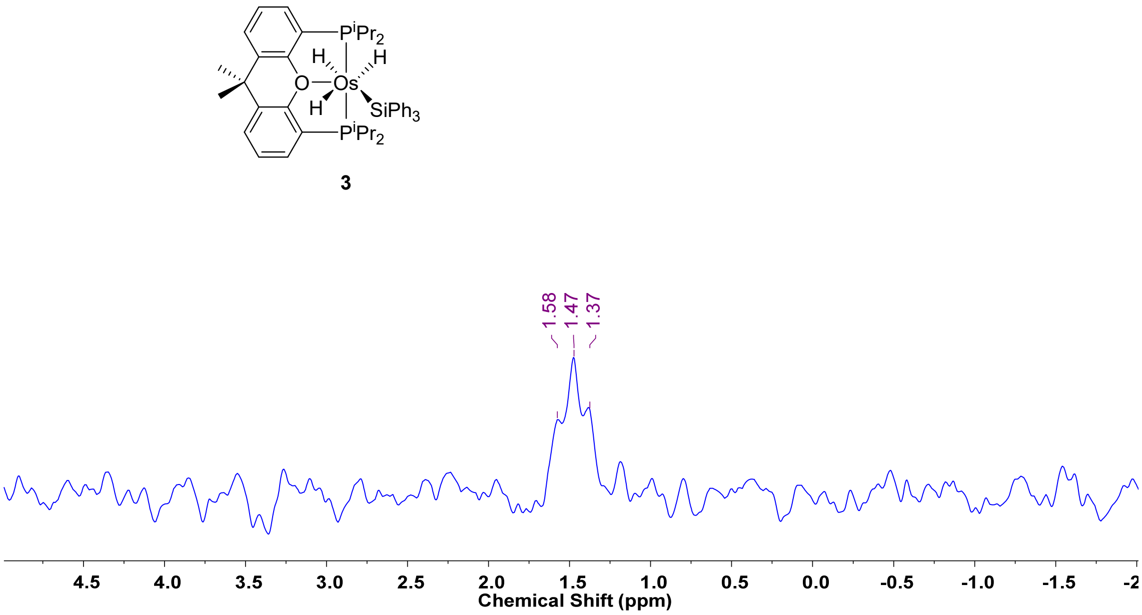

Figure S13. $^{29}Si\{^{1}H\}$ NMR spectrum (59.63 MHz, C_6D_6, 298 K) of **3**.

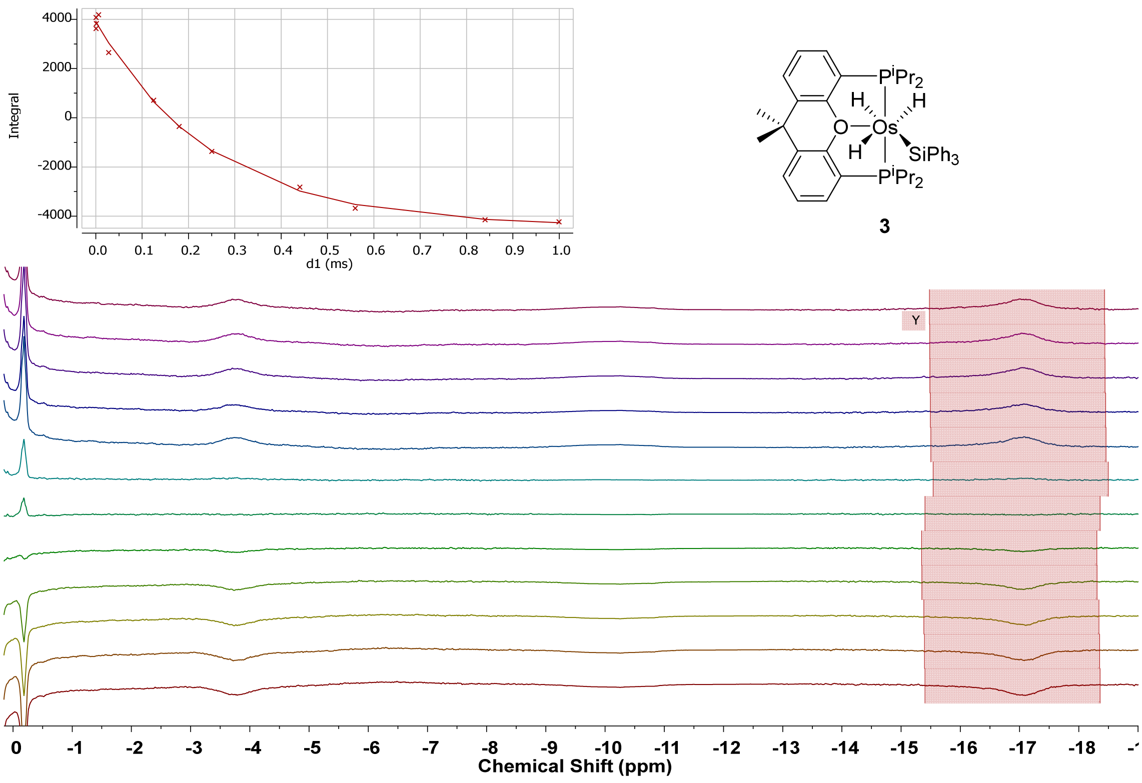

Figure S14. ^{1}H NMR $T_{1(min)}$ relaxation time measurement (300.13 MHz, toluene-d_8, 230 K, OsH) of **3**.

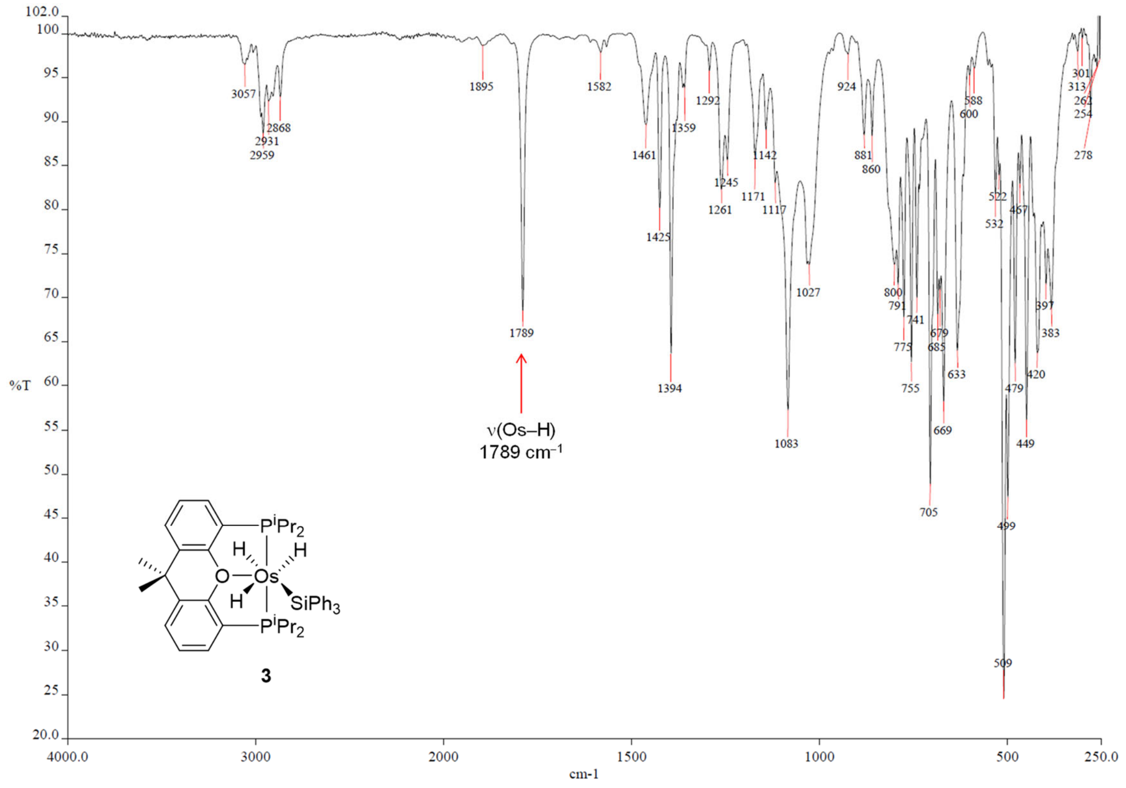

Figure S15. ATR-IR spectrum of **3**.

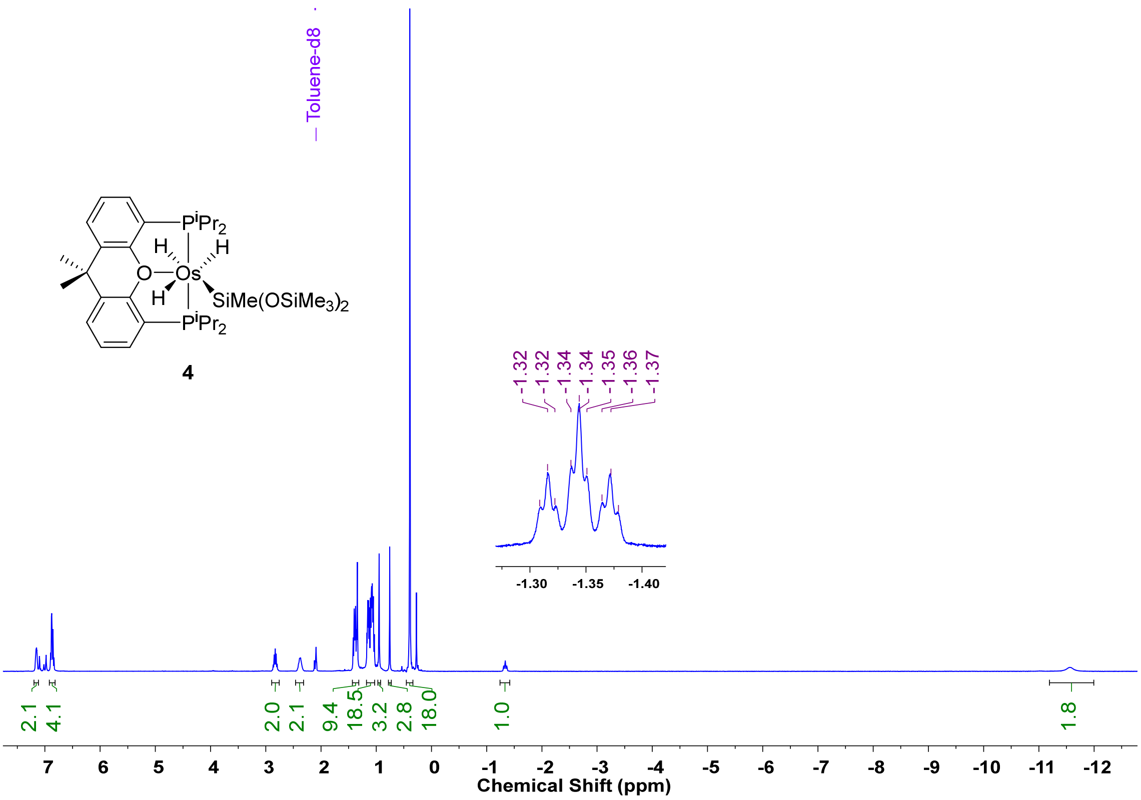

Figure S16. ^{1}H NMR spectrum (400.13 MHz, toluene-*d*8, 298 K) of **4**.

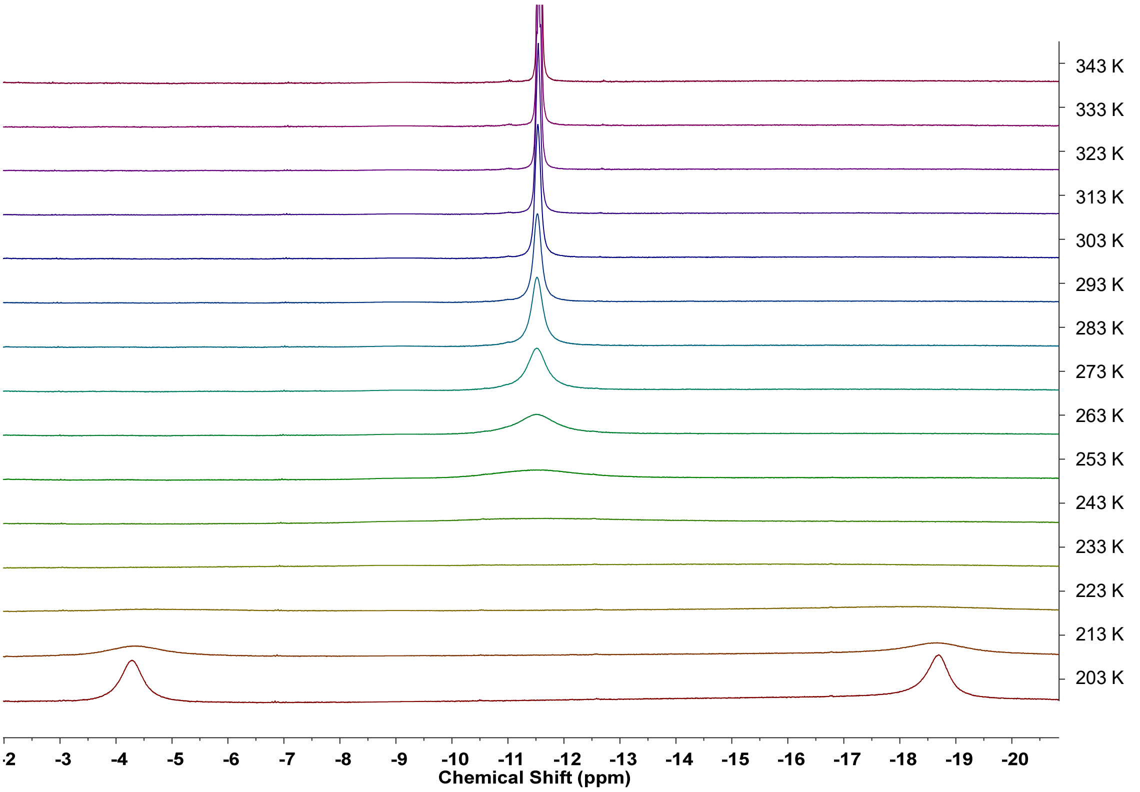

Figure S17. High-field region of ^{1}H NMR spectra (400.13 MHz, toluene-*d*8, 343-203 K) of **4**.

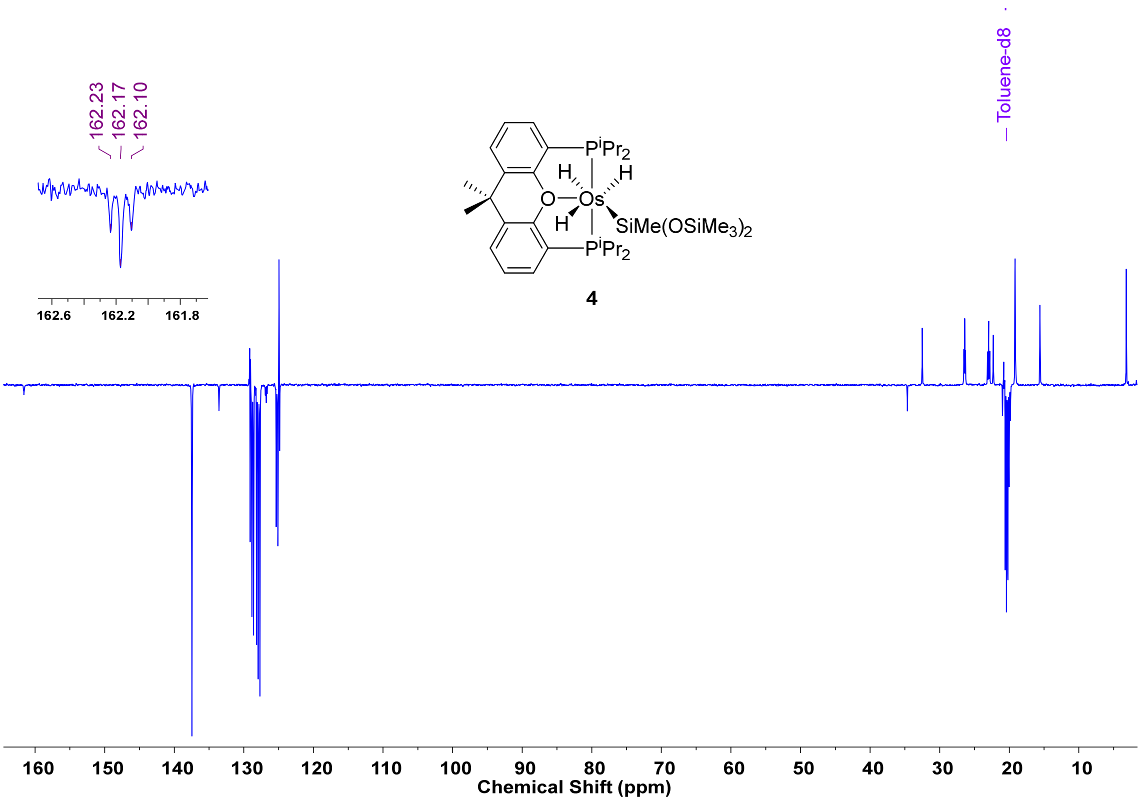

Figure S18. $^{13}C\{^{1}H\}$-APT NMR spectrum (100.62 MHz, toluene-d_8, 298 K) of **4**.

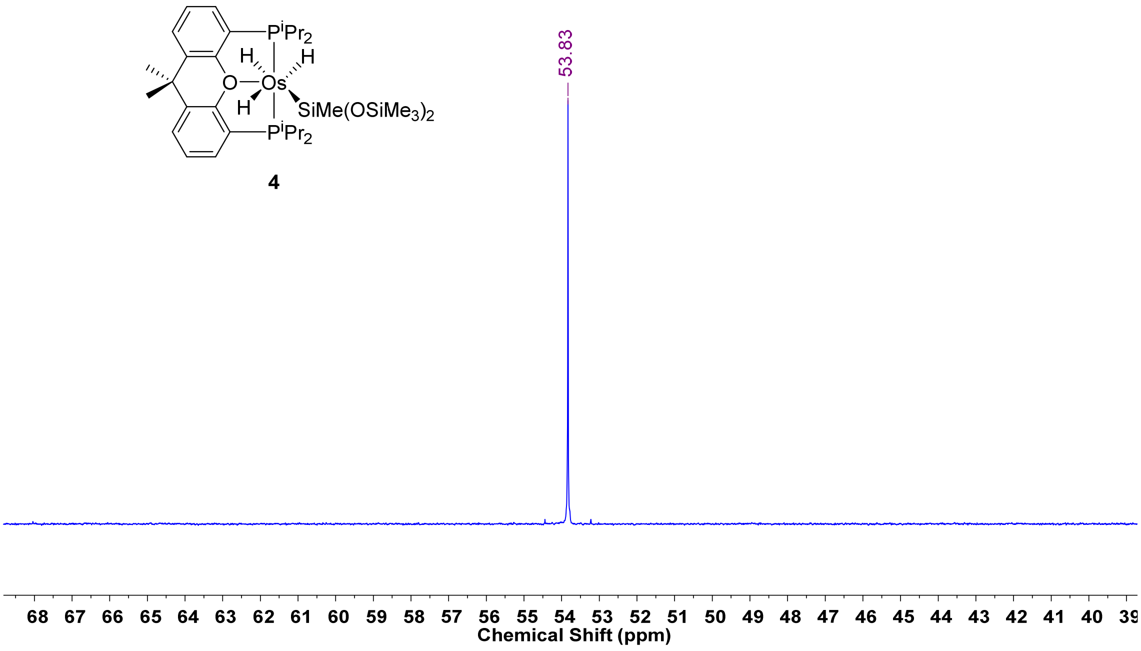

Figure S19. $^{31}P\{^{1}H\}$ NMR spectrum (161.98 MHz, toluene-d_8, 298 K) of **4**.

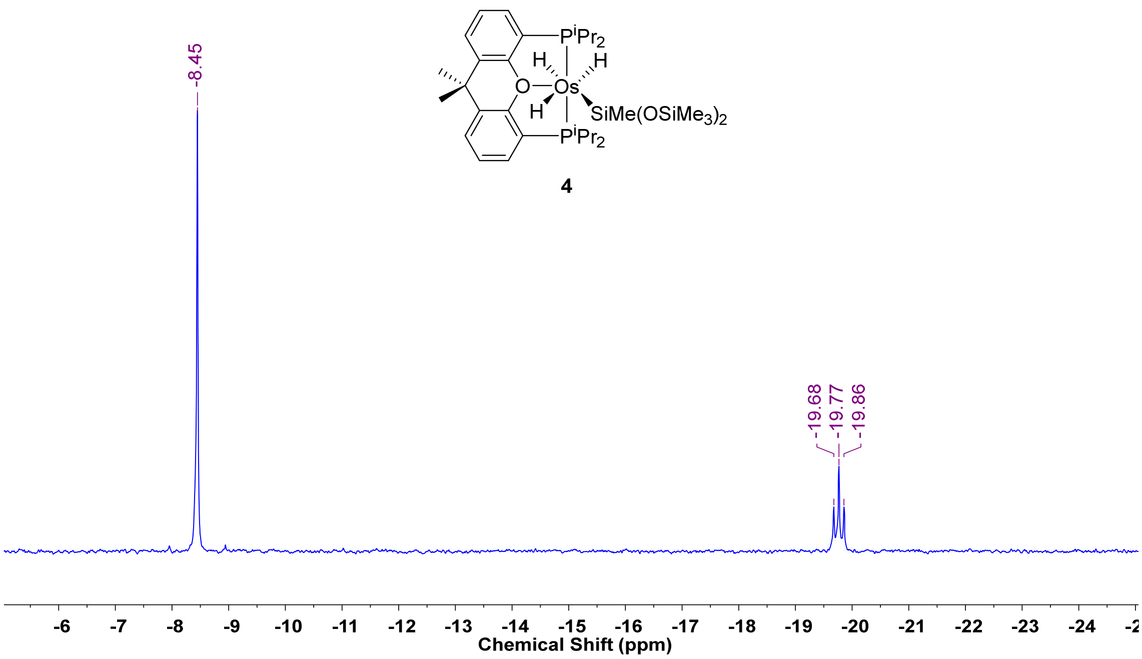

Figure S20. $^{29}Si\{^{1}H\}$ NMR spectrum (59.63 MHz, toluene-d_8, 298 K) of **4**.

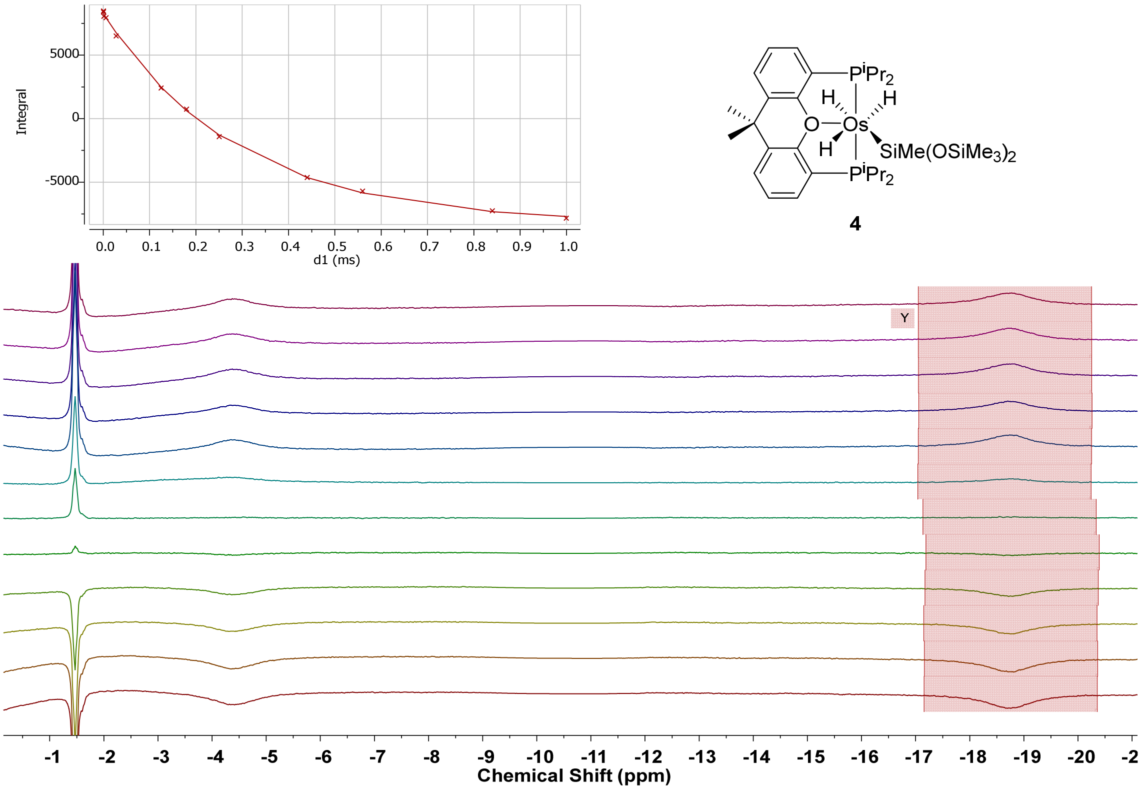

Figure S21. ^{1}H NMR $T_{1(min)}$ relaxation time measurement (300.13 MHz, toluene-d_8, 223 K, OsH) of **4**.

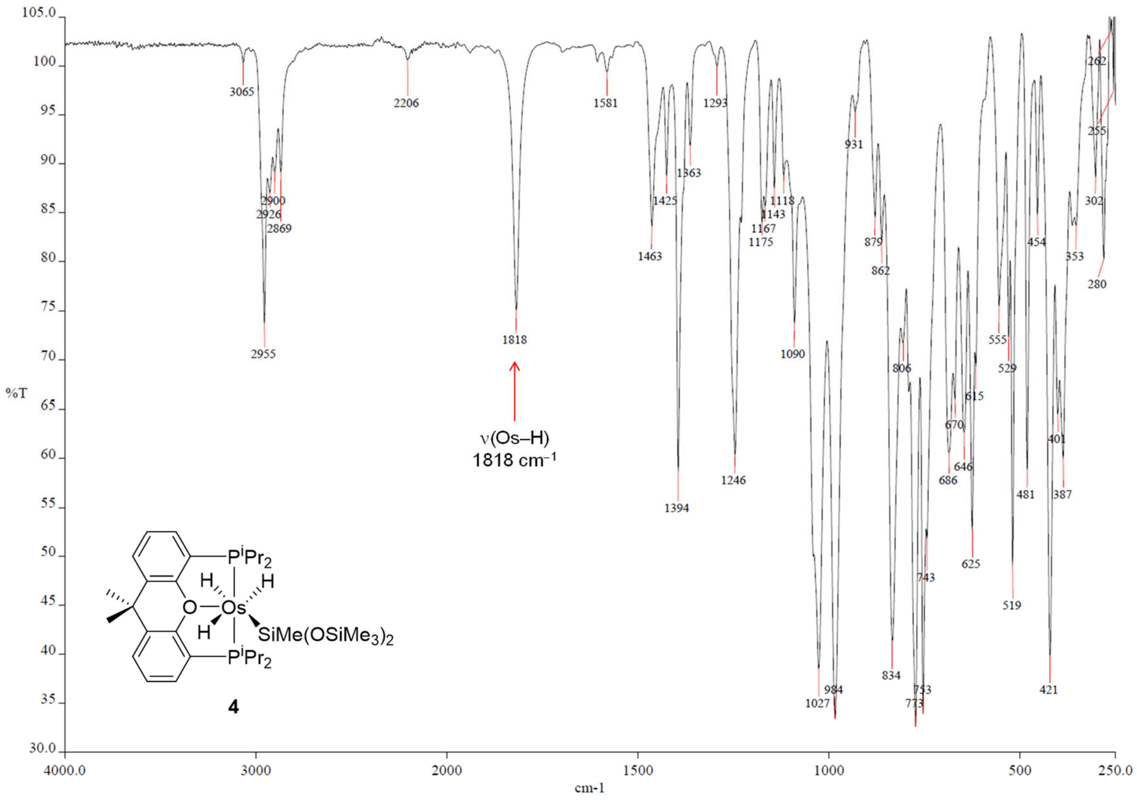

Figure S22. ATR-IR spectrum of **4**.

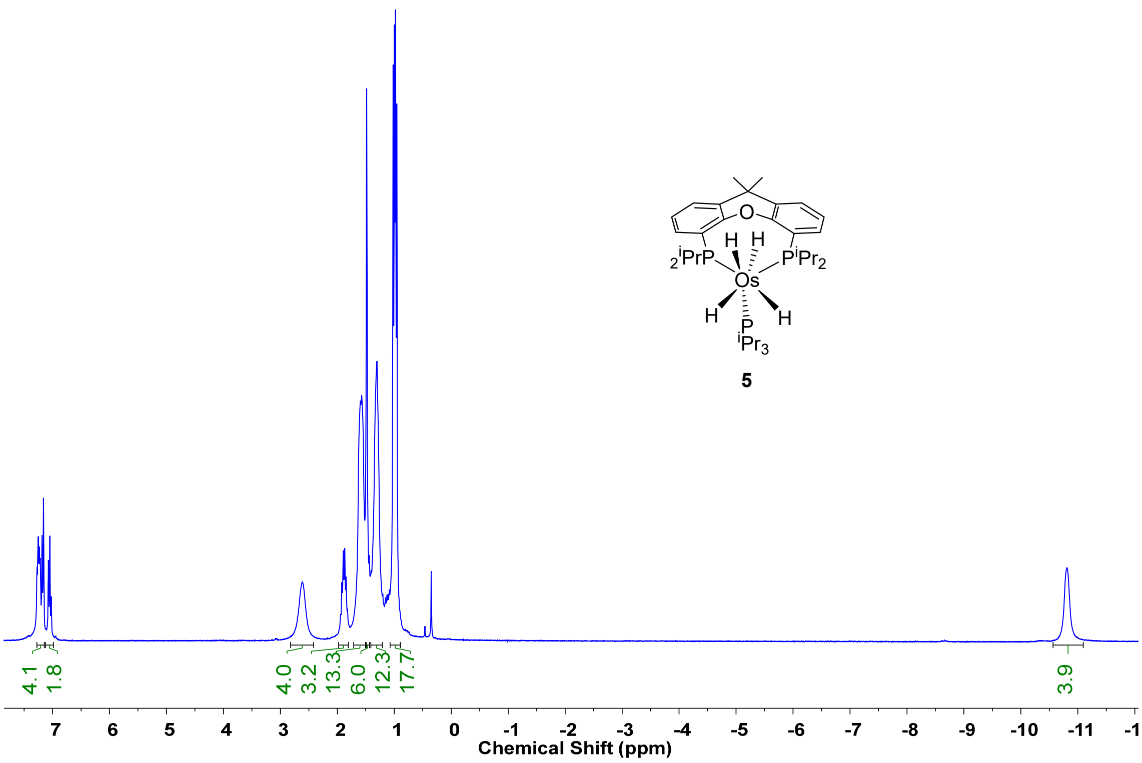

Figure S23. ^{1}H NMR spectrum (300.13 MHz, C_6D_6, 298 K) of **5**.

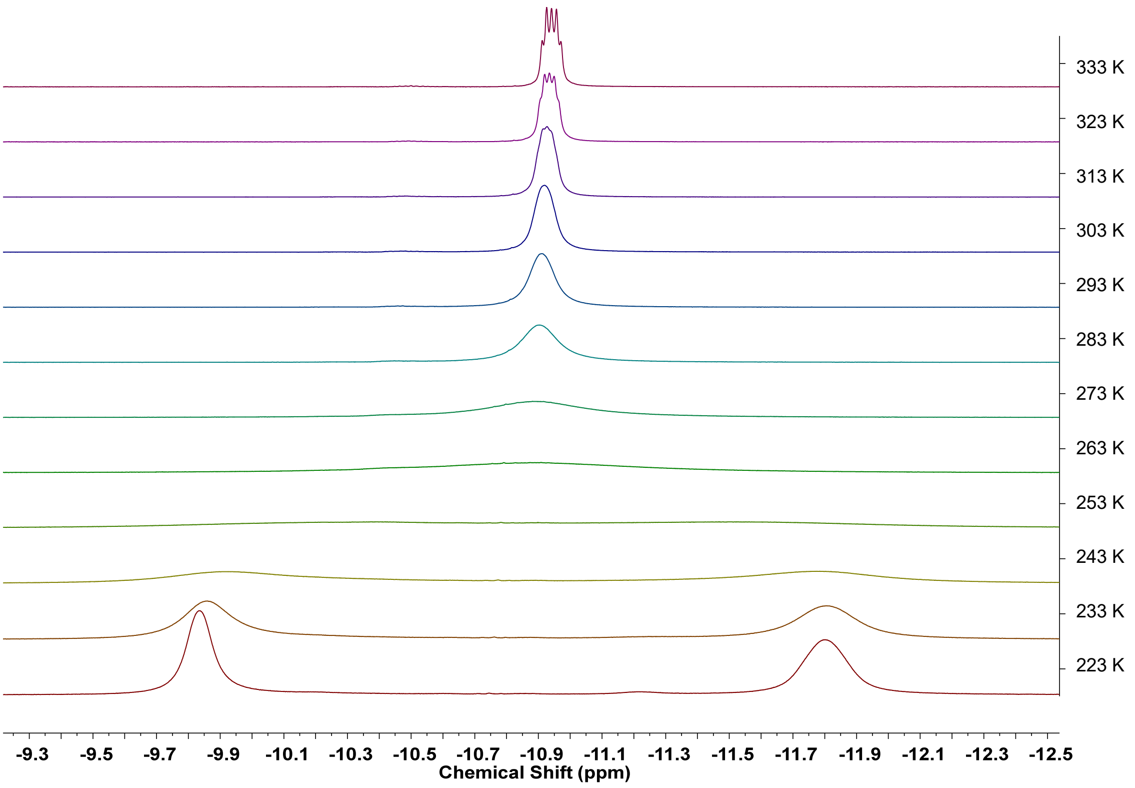

Figure S24. High-field region of ^{1}H NMR spectra (400.13 MHz, toluene-d_8, 333-223 K) of **5**.

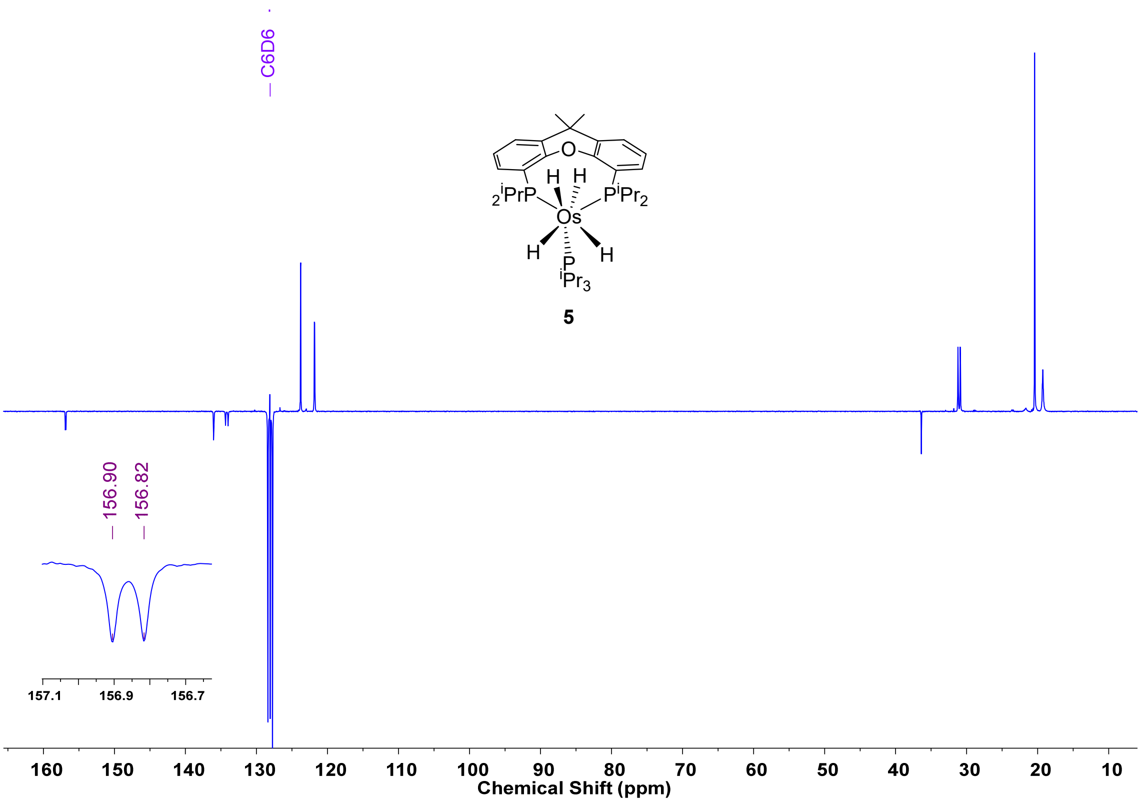

Figure S25. $^{13}C\{^{1}H\}$-APT NMR spectrum (75.47 MHz, C_6D_6, 298 K) of **5**.

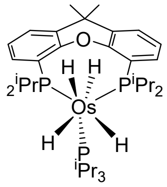

5

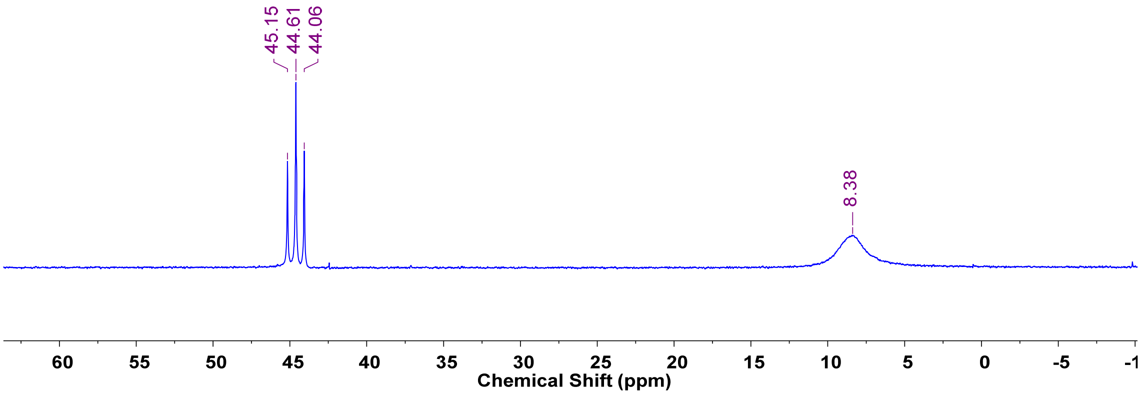

Figure S26. $^{31}P\{^{1}H\}$ NMR spectrum (121.49 MHz, C_6D_6, 298 K) of **5**.

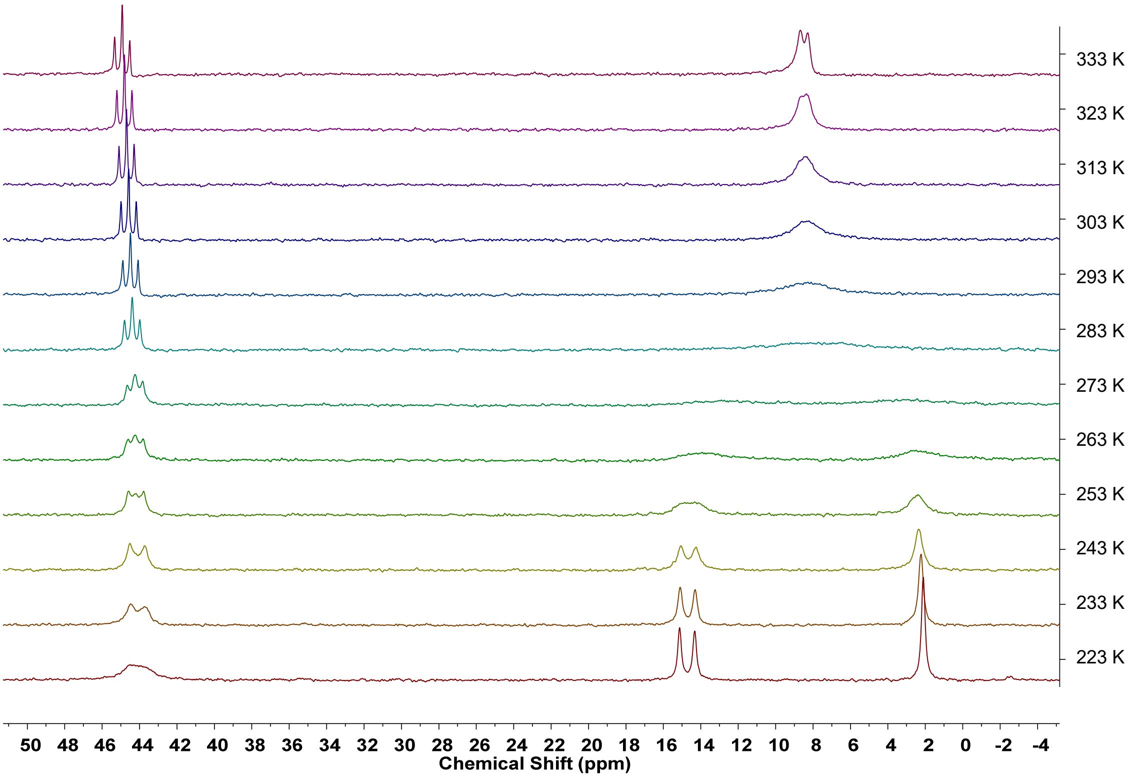

Figure S27. $^{31}P\{^{1}H\}$ NMR spectra (161.98 MHz, toluene-d_8, 333-223 K) of **5**.

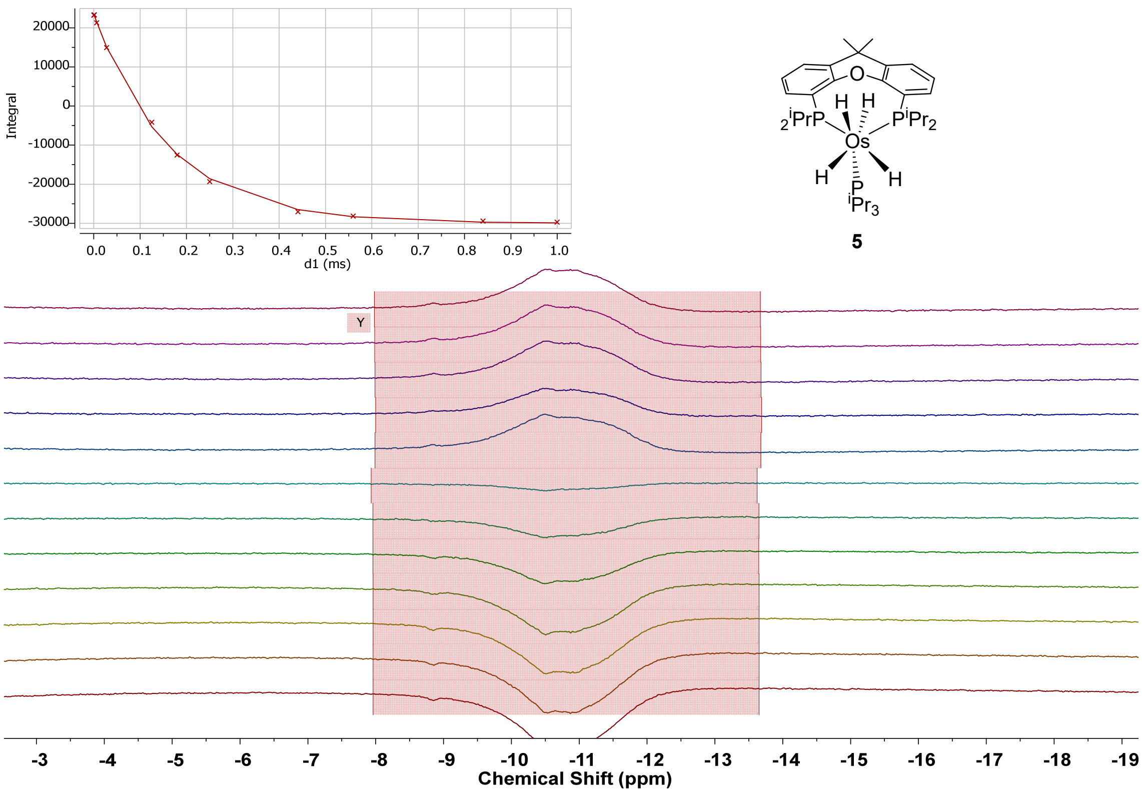

Figure S28. ^{1}H NMR $T_{1(min)}$ relaxation time measurement (300.13 MHz, toluene-d_8, 253 K, OsH) of **5**.

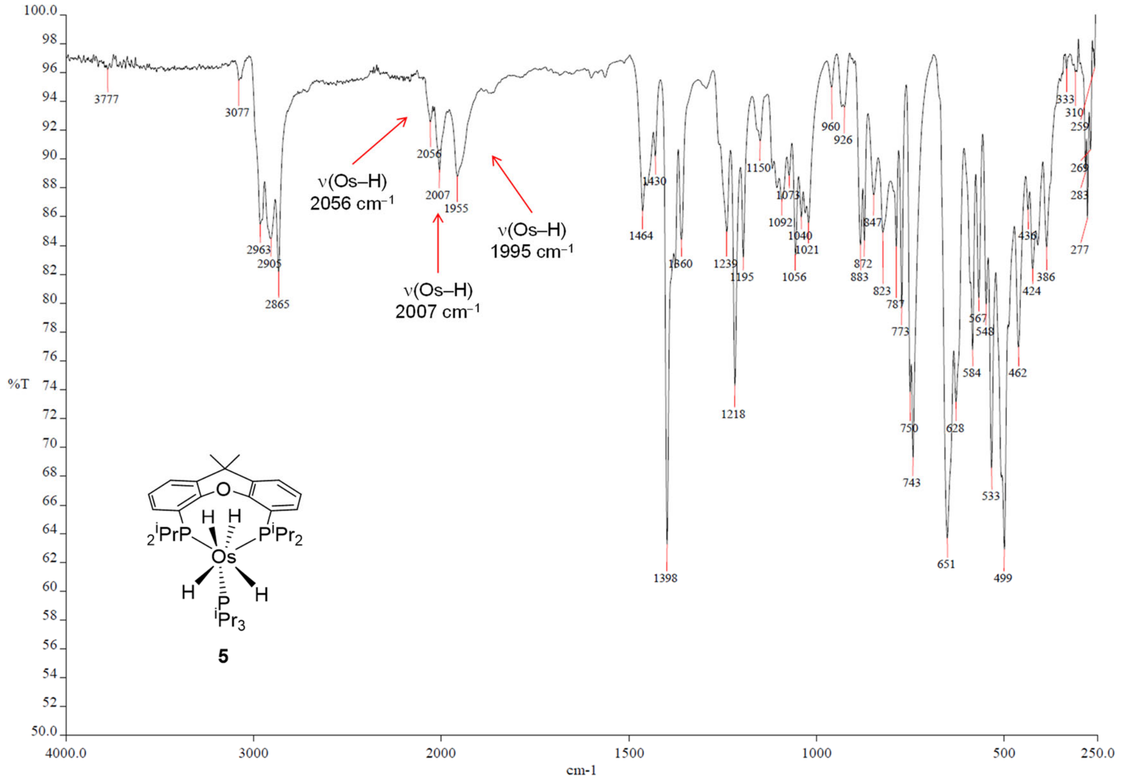

Figure S29. ATR-IR spectrum of **5**.

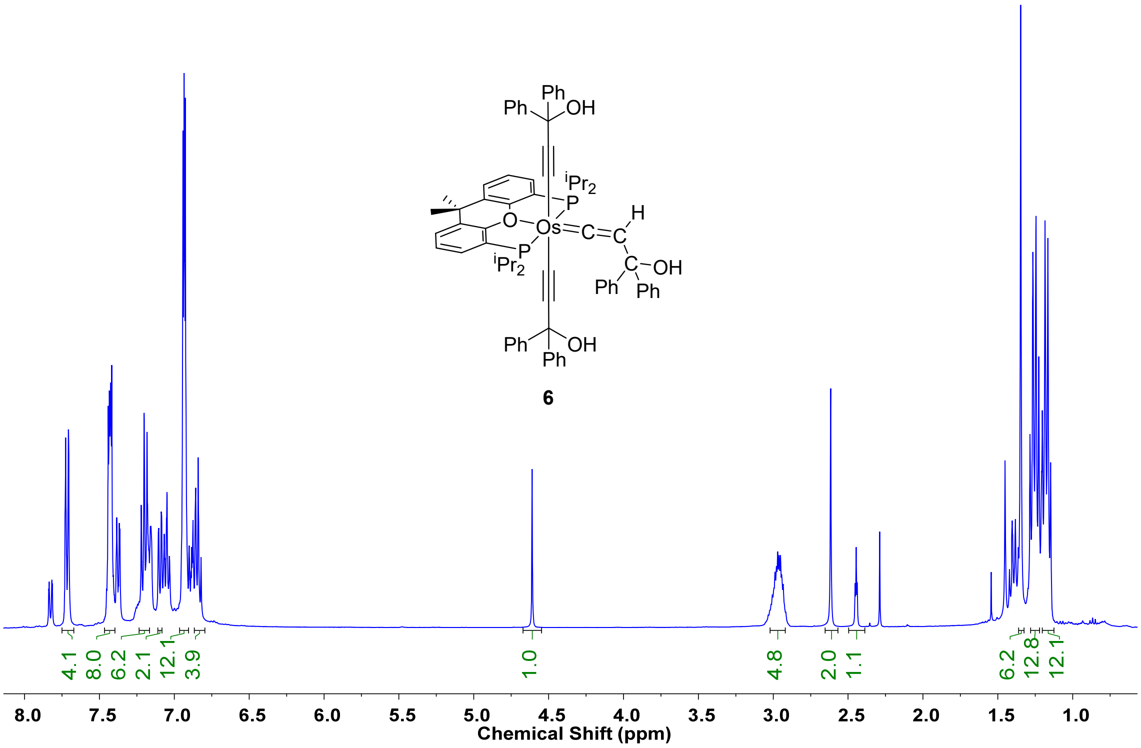

Figure S30. ^{1}H NMR spectrum (400.13 MHz, C_6D_6, 298 K) of **6**.

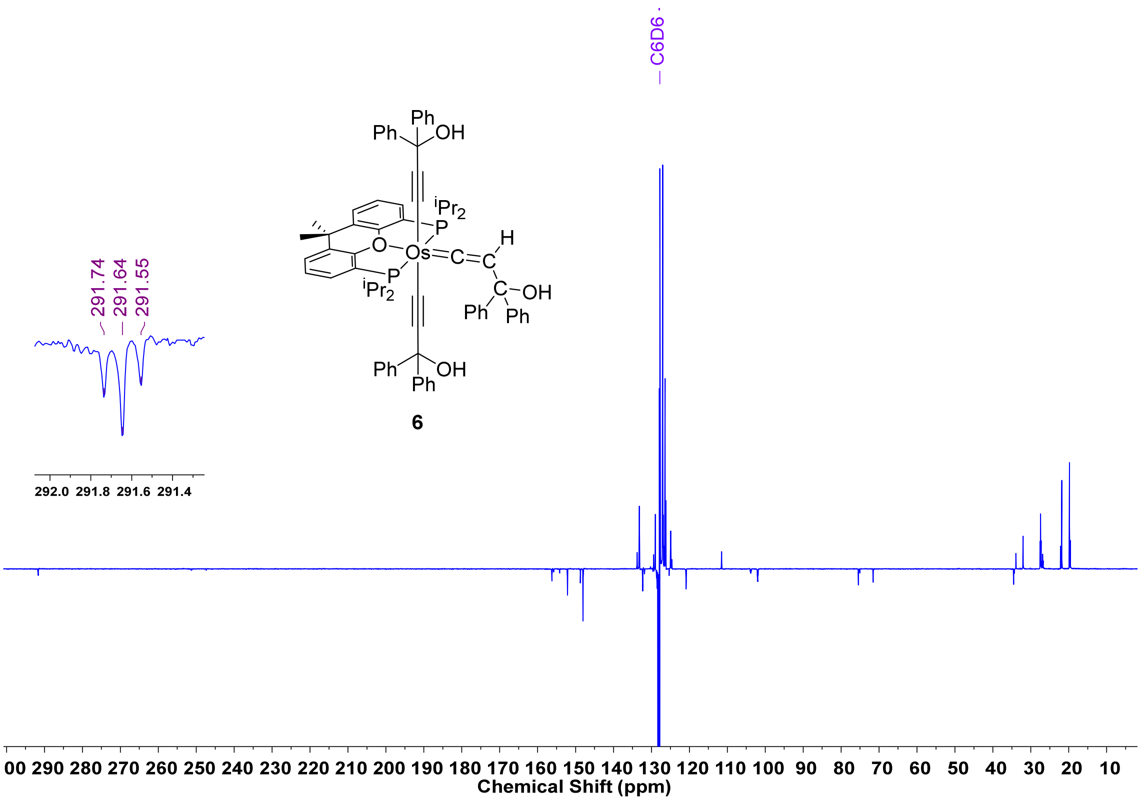

Figure S31. $^{13}C\{^{1}H\}$-APT NMR spectrum (100.63 MHz, C_6D_6, 298 K) of **6**.

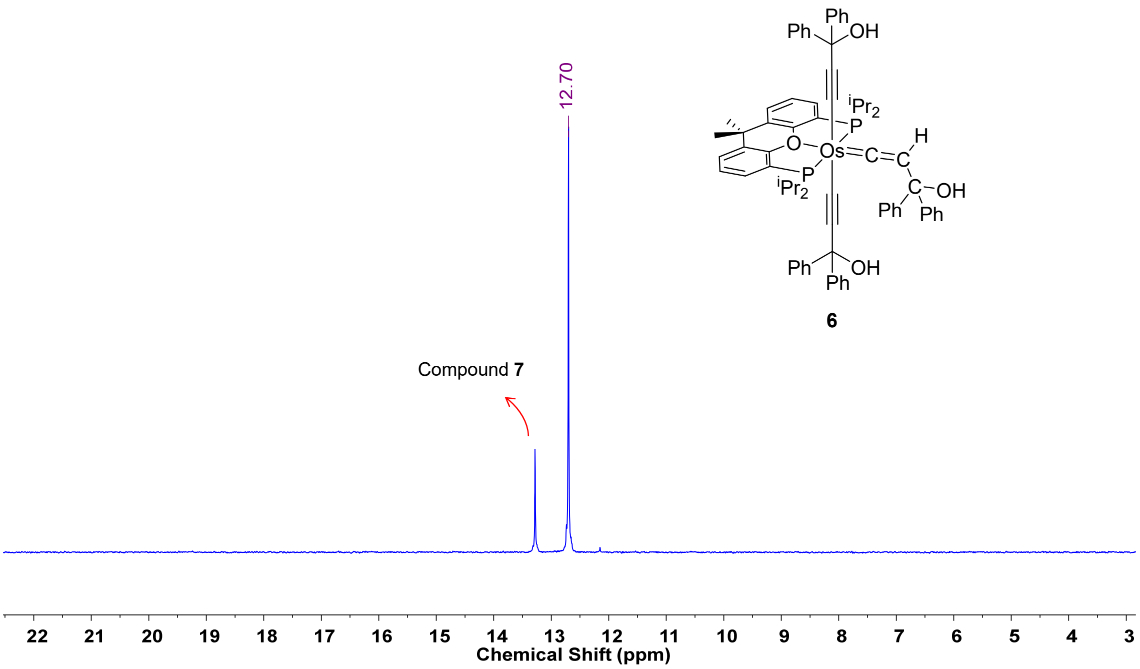

Figure S32. $^{31}P\{^{1}H\}$ NMR spectrum (161.98 MHz, C_6D_6, 298 K) of **6**.

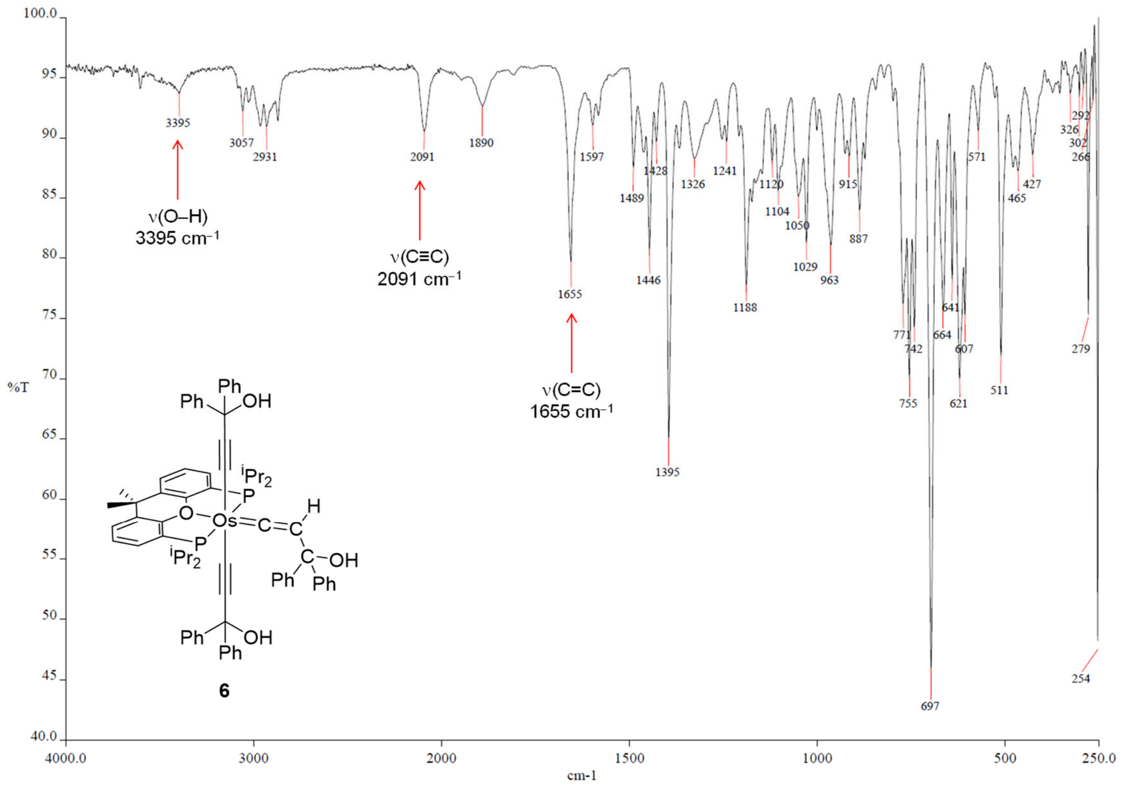

Figure S33. ATR-IR spectrum of **6**.

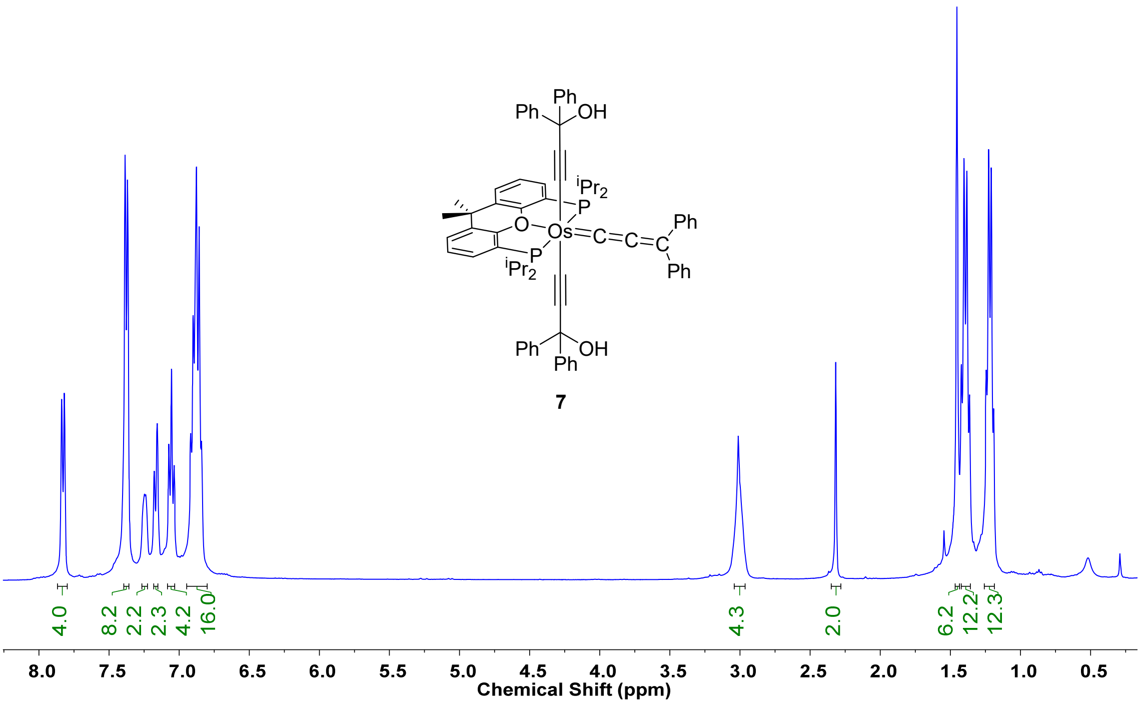

Figure S34. ^{1}H NMR spectrum (400.13 MHz, C_6D_6, 298 K) of **7**.

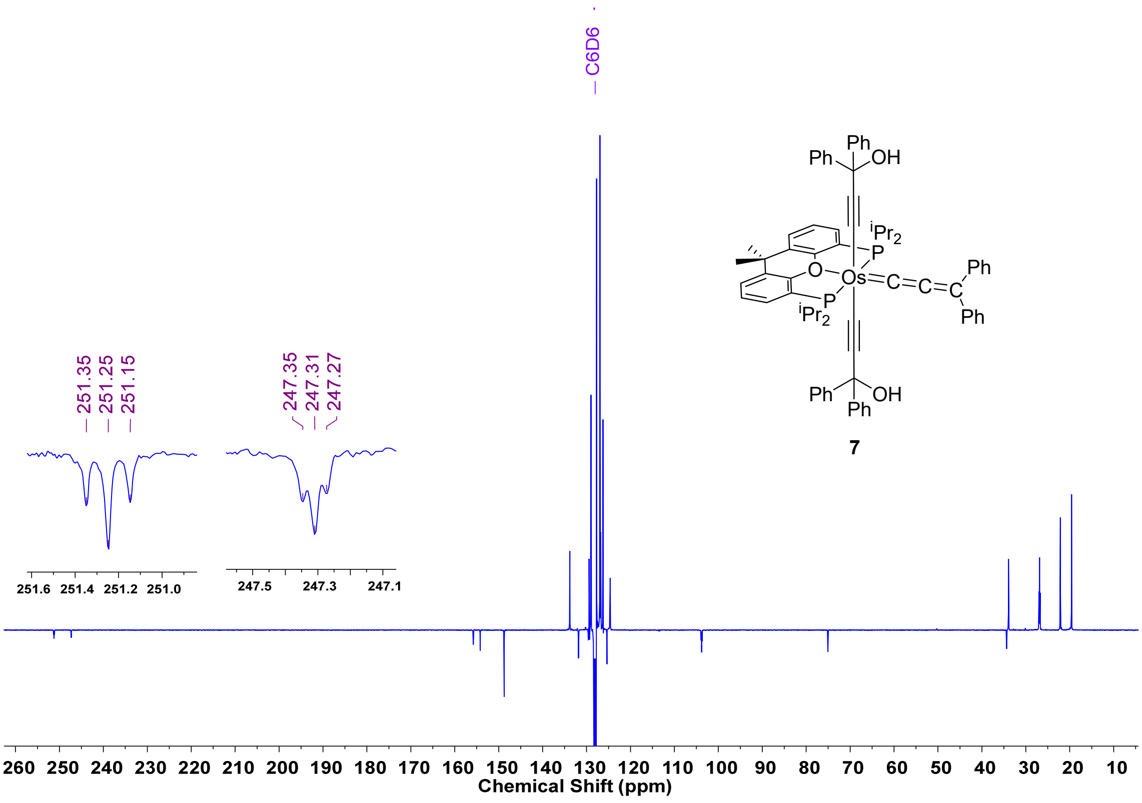

Figure S35. $^{13}C\{^{1}H\}$-APT NMR spectrum (100.63 MHz, C_6D_6, 298 K) of **7**.

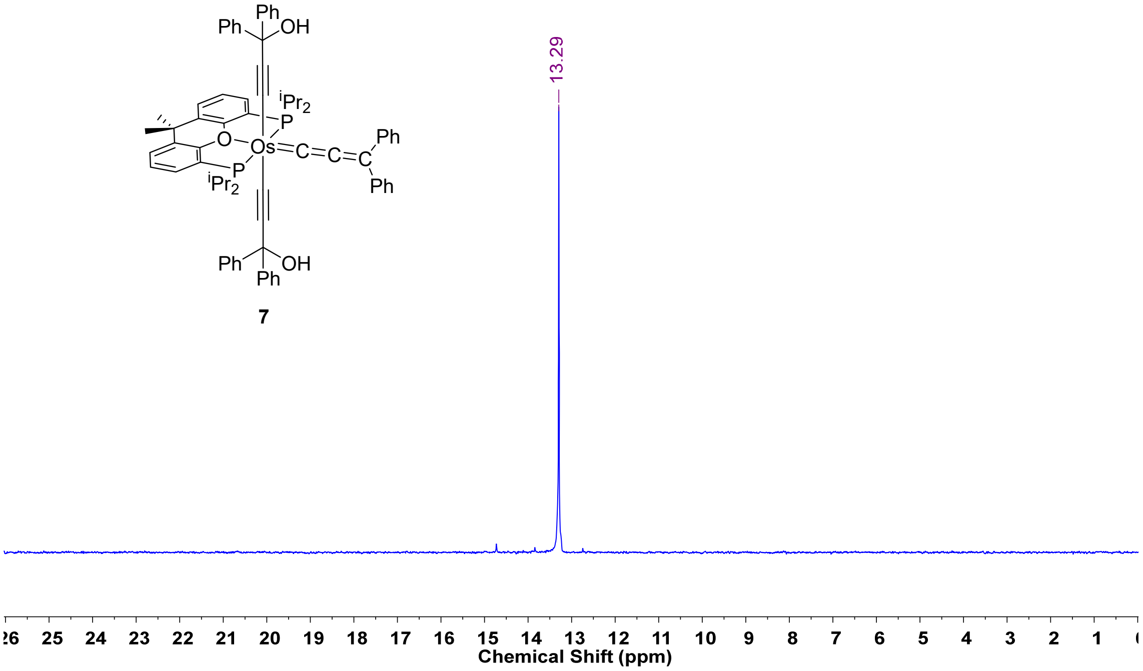

Figure S36. $^{31}P\{^{1}H\}$ NMR spectrum (161.98 MHz, C_6D_6, 298 K) of **7**.

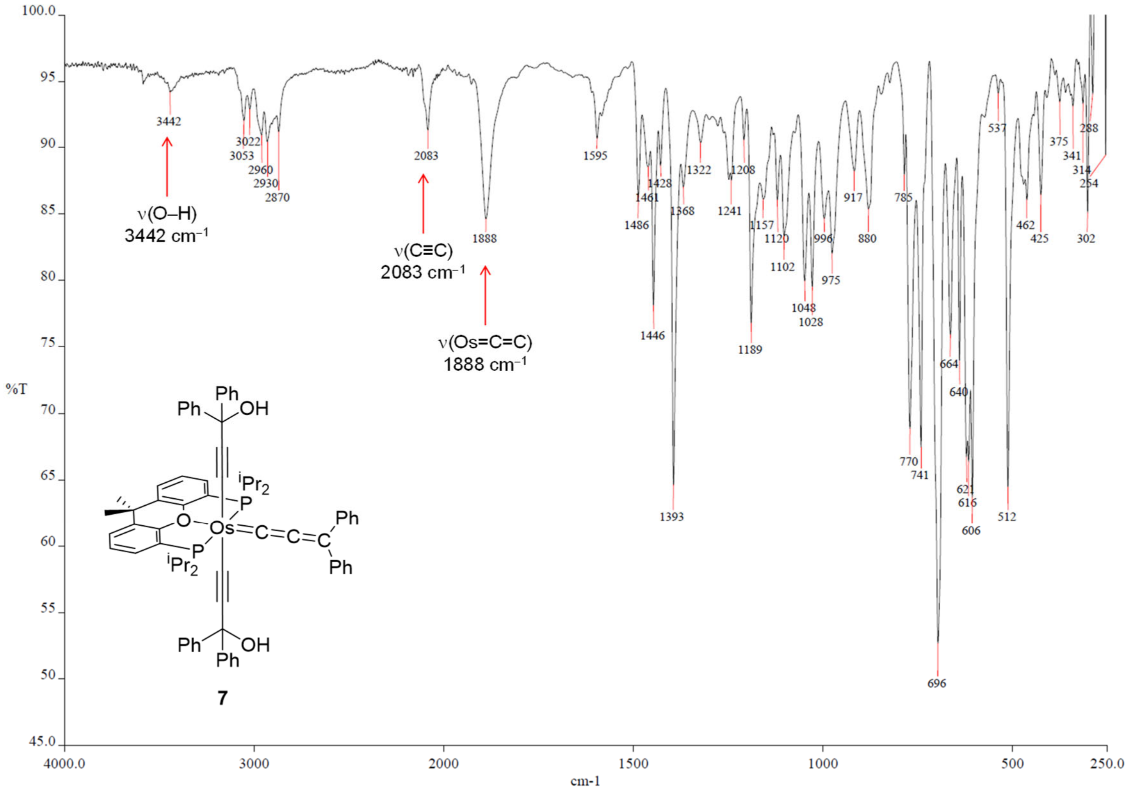

Figure S37. ATR-IR spectrum of **7**.

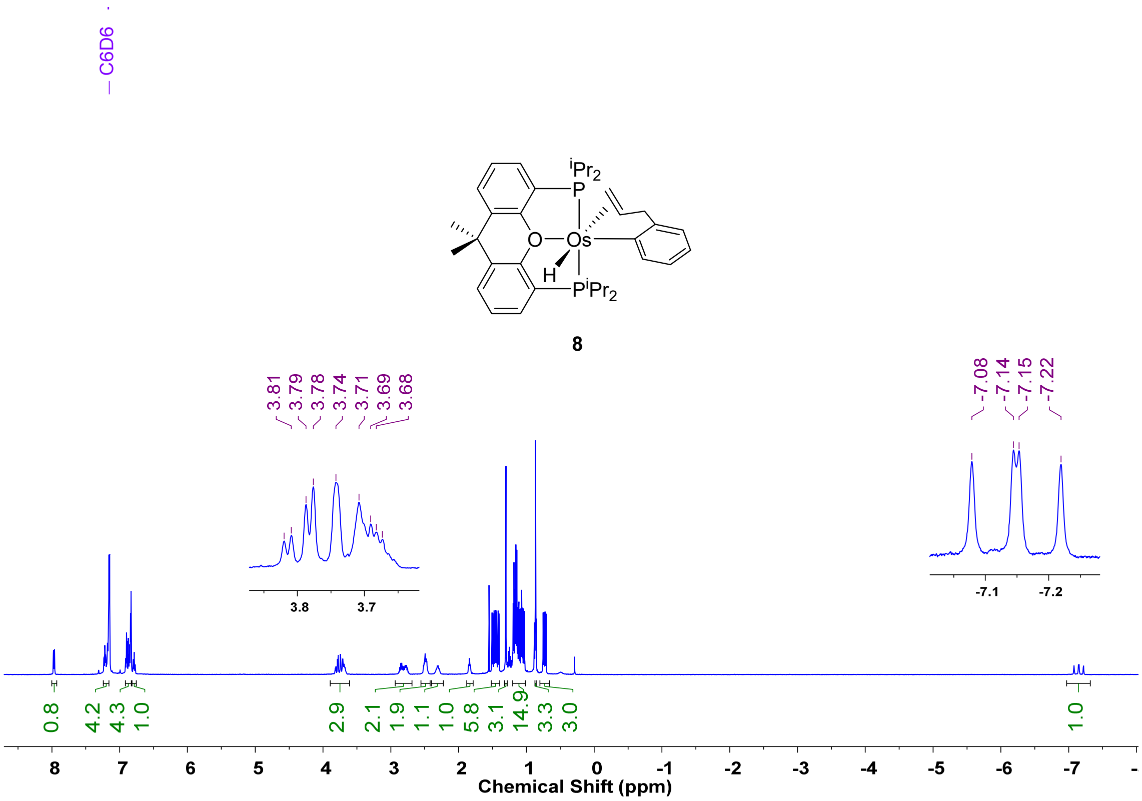

Figure S38. ^{1}H NMR spectrum (500.12 MHz, C_6D_6, 298 K) of **8**.

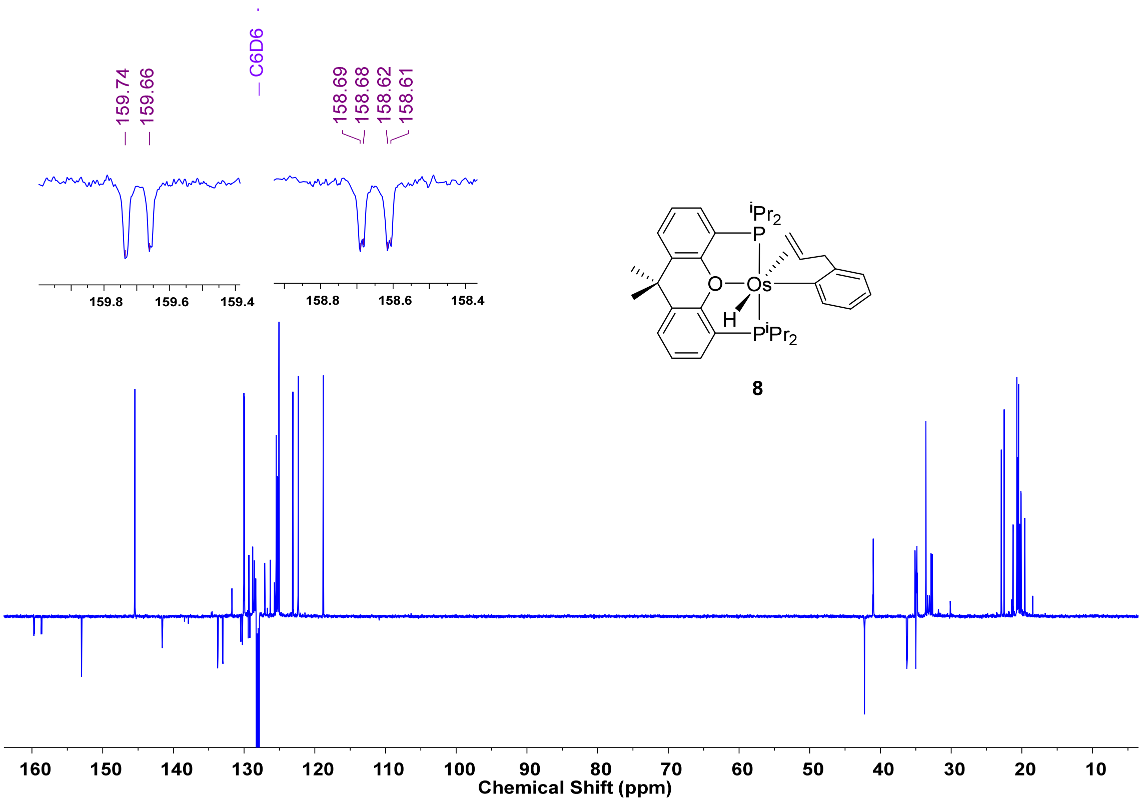

Figure S39. $^{13}C\{^{1}H\}$-APT NMR spectrum (125.77 MHz, C_6D_6, 298 K) of **8**.

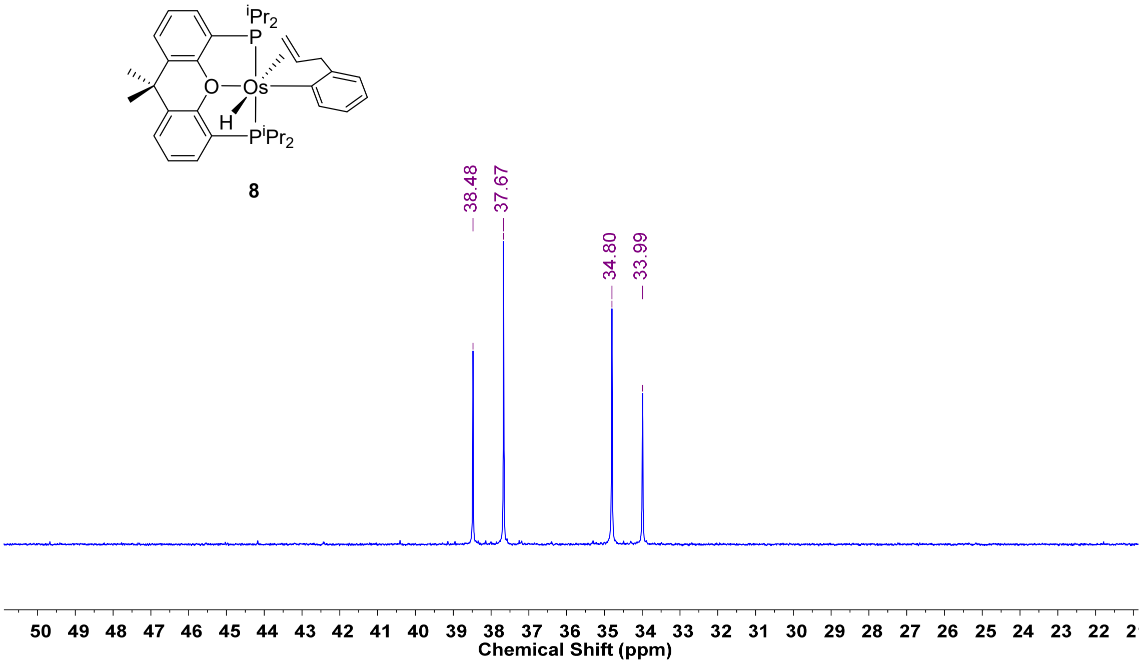

Figure S40. $^{31}P\{^{1}H\}$ NMR spectrum (202.46 MHz, C_6D_6, 298 K) of **8**.

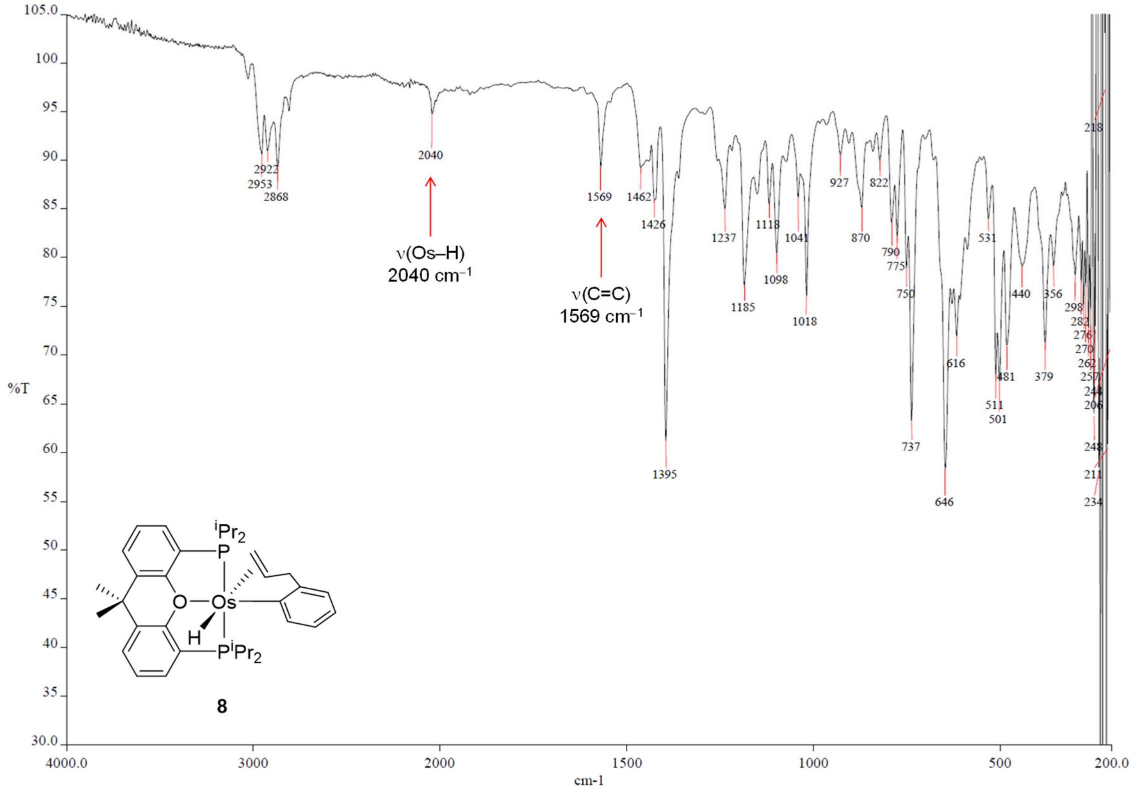

Figure S41. ATR-IR spectrum of **8**.

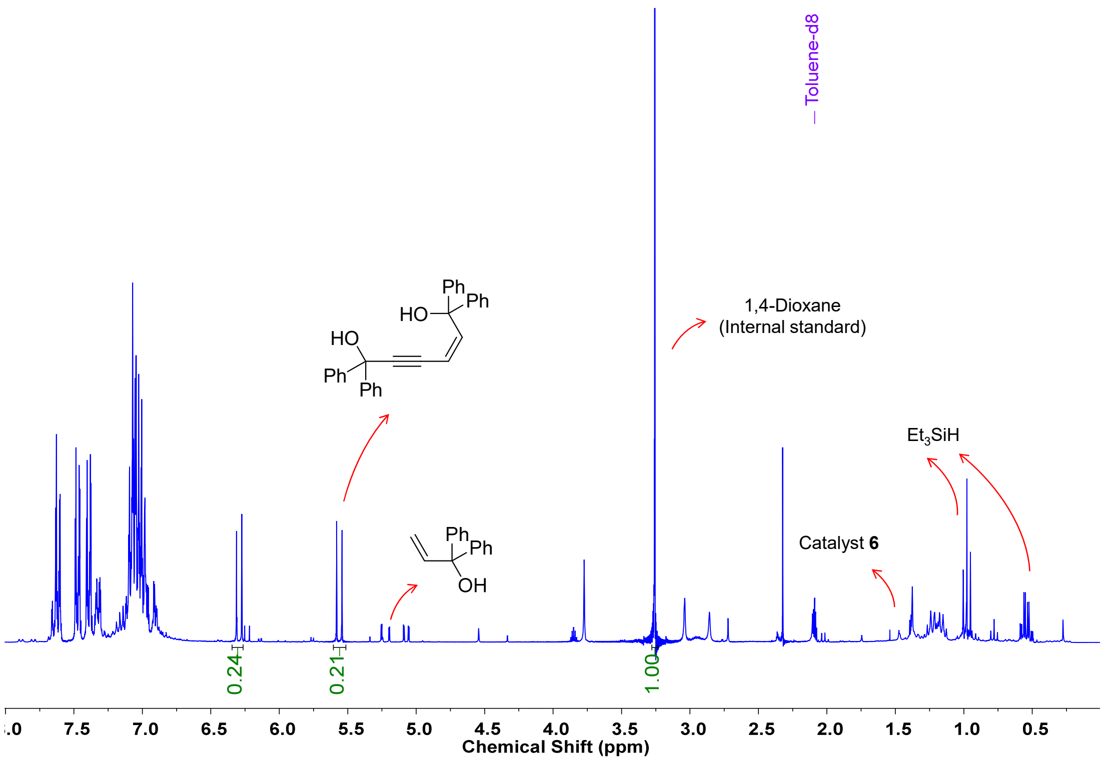

Figure S42. ^{1}H NMR spectrum (300.13 MHz, toluene-d_8, 298 K) of the dimerization of 1,1-diphenyl-2-propyn-1-ol at 110 ºC for 2 h.

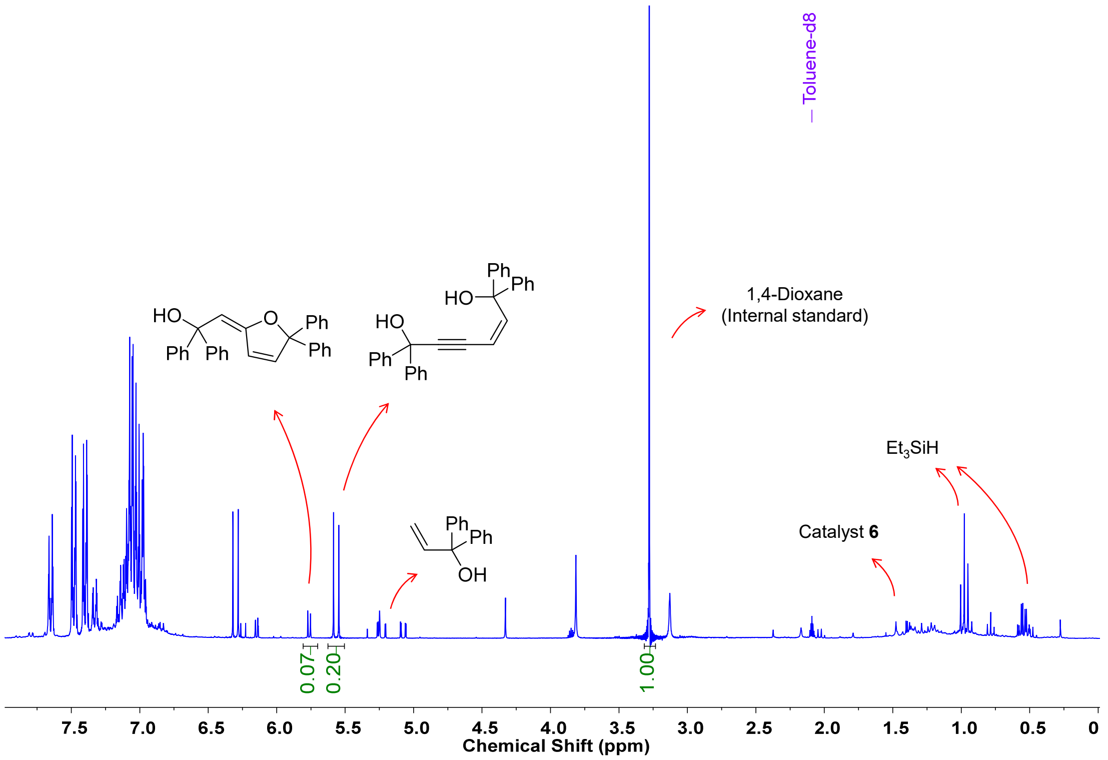

Figure S43. ^{1}H NMR spectrum (300.13 MHz, toluene-d_8, 298 K) of the dimerization of 1,1-diphenyl-2-propyn-1-ol at 110 ºC for 7 h.

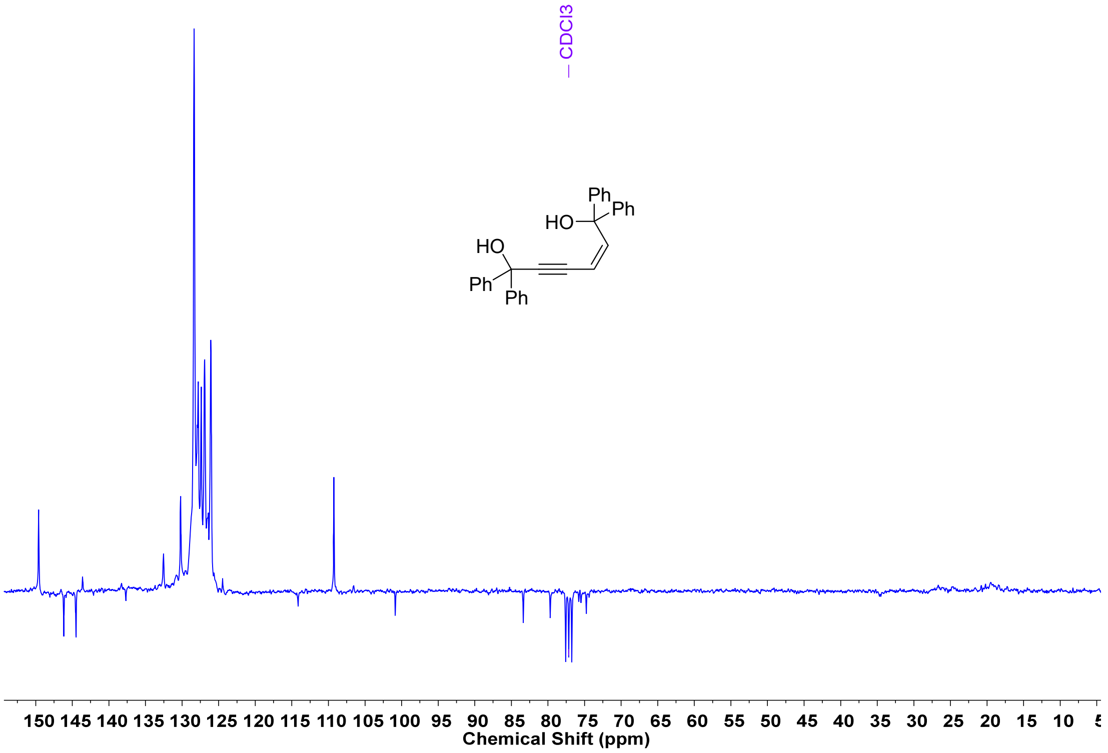

Figure S44. $^{13}C\{^{1}H\}$-APT NMR spectrum (75.47 MHz, $CDCl_3$, 298 K) of (*Z*)-1,1,6,6-tetraphenylhex-2-en-4-yne-1,6-diol.

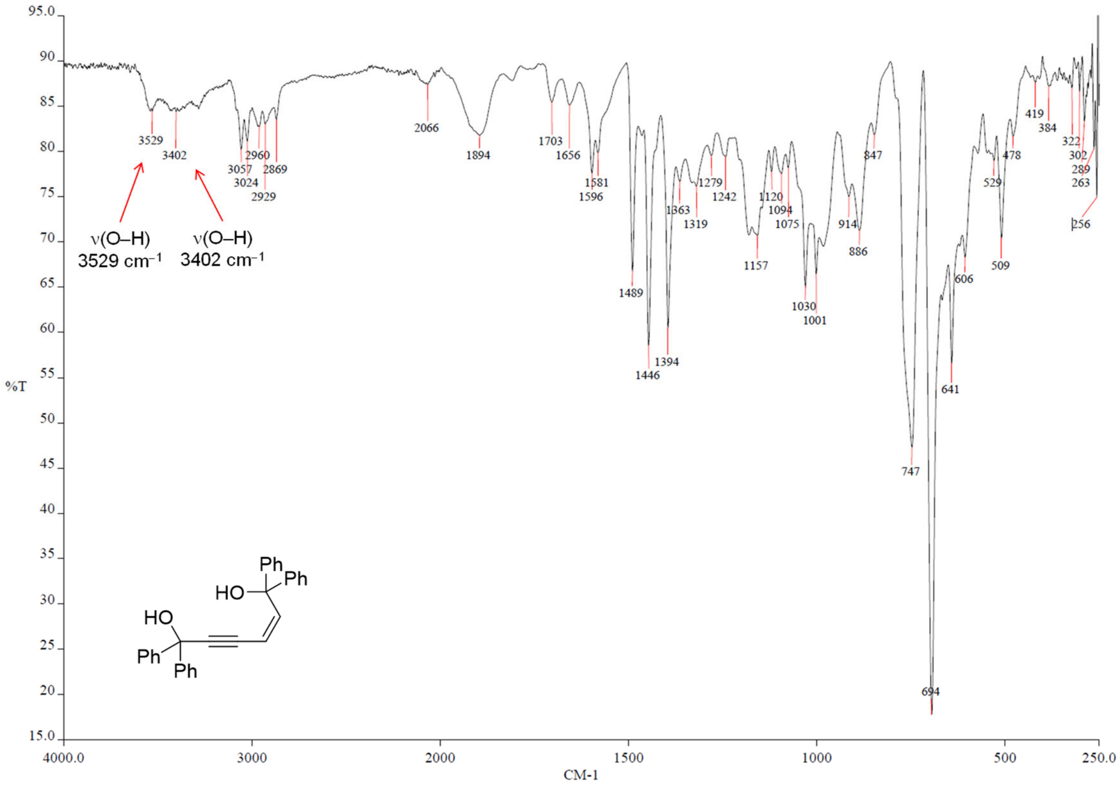

Figure S45. ATR-IR spectrum of (*Z*)-1,1,6,6-tetraphenylhex-2-en-4-yne-1,6-diol.

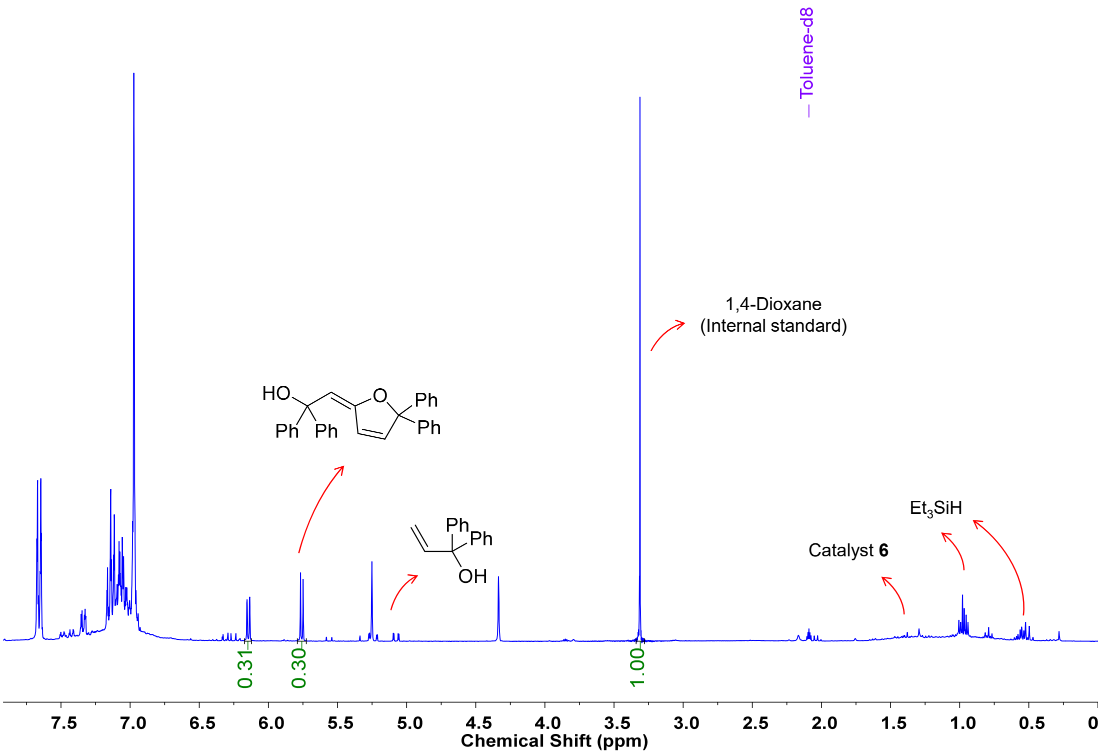

Figure S46. ^{1}H NMR spectrum (300.13 MHz, toluene-d_8, 298 K) of the dimerization of 1,1-diphenyl-2-propyn-1-ol at 140 ºC for 24 h.

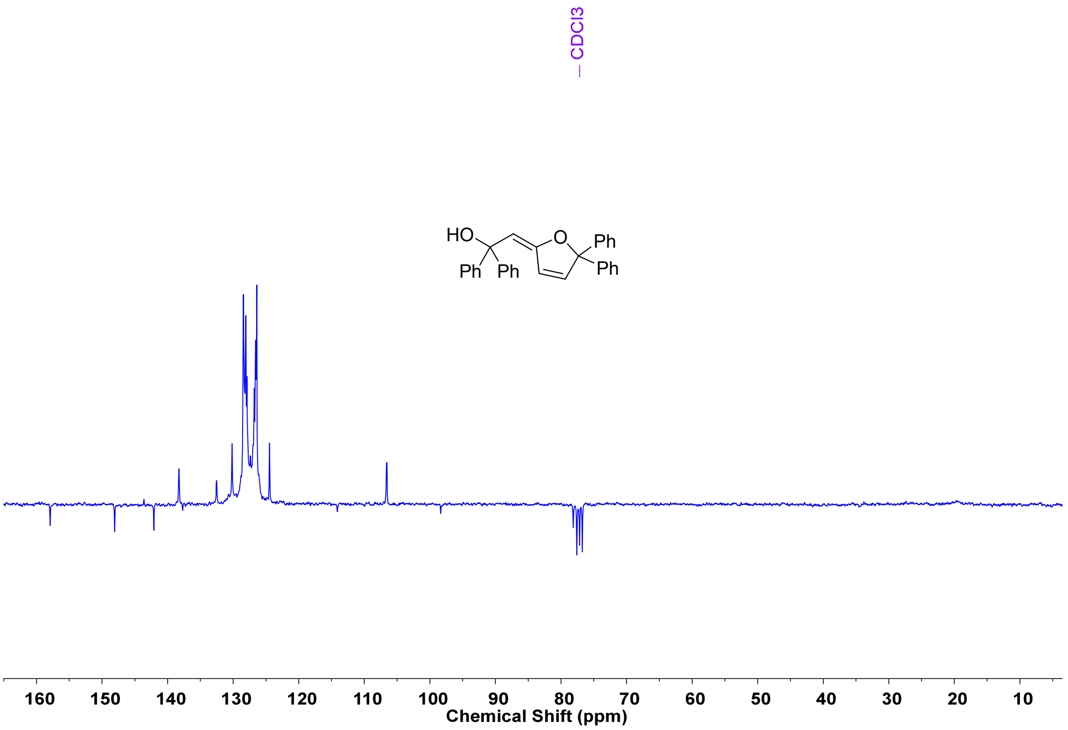

Figure S47. $^{13}C\{^{1}H\}$-APT NMR spectrum (75.47 MHz, $CDCl_3$, 298 K) of (*E*)-2-(5,5-diphenylfuran-2(*5H*)-ylidene)-1,1-diphenylethan-1-ol.

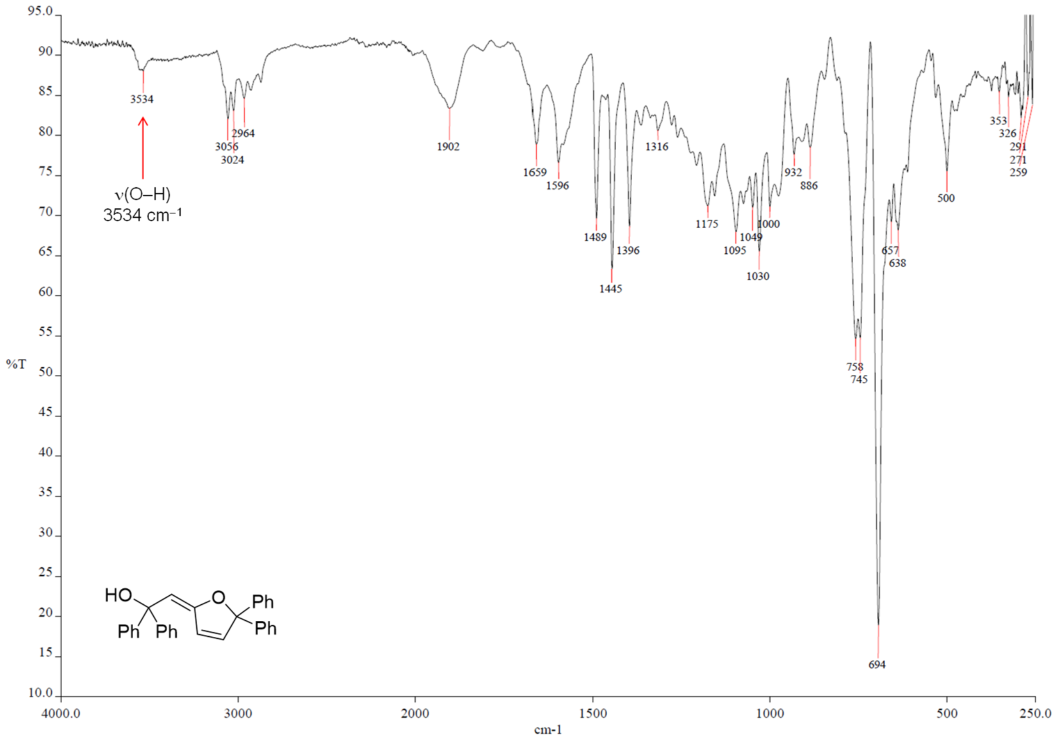

Figure S48. ATR-IR spectrum of (*E*)-2-(5,5-diphenylfuran-2(*5H*)-ylidene)-1,1-diphenylethan-1-ol.

– Deuteration Experiments

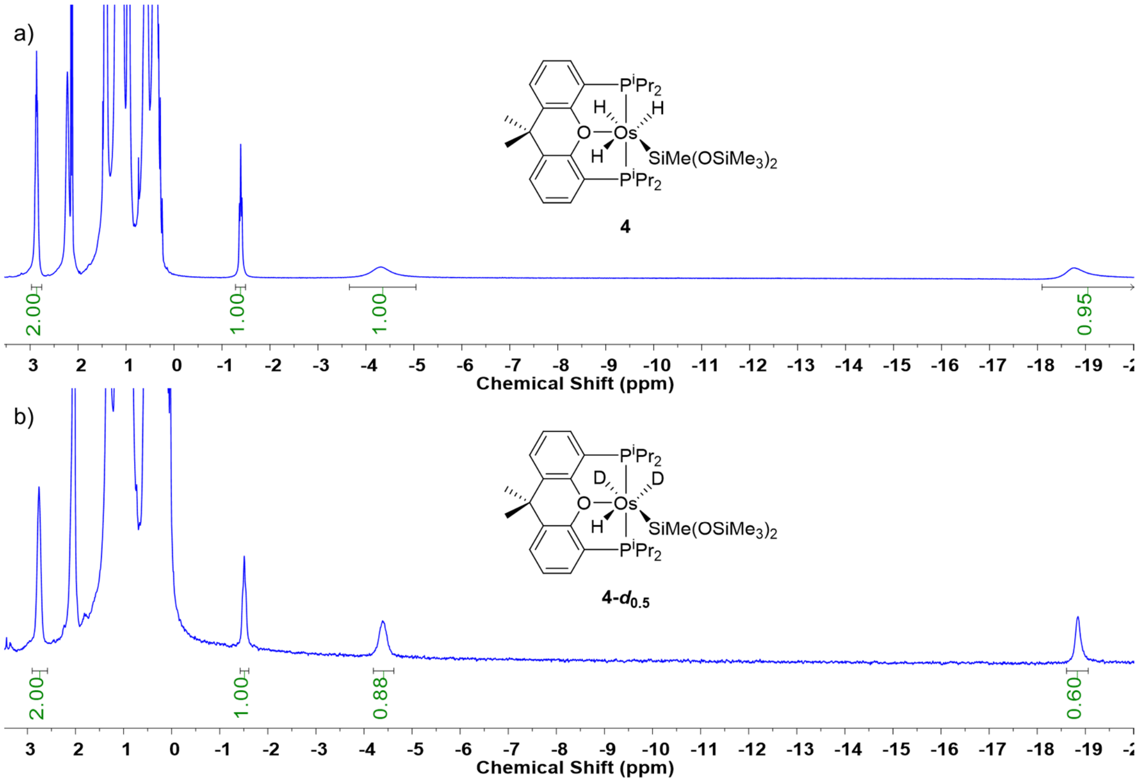

Figure S49. High field region of ^{1}H NMR spectra (400.13, toluene-d_8, 203 K) of complexes **4** (a) and **4-$d_{0.5}$** (b).

– Kinetic Experiments

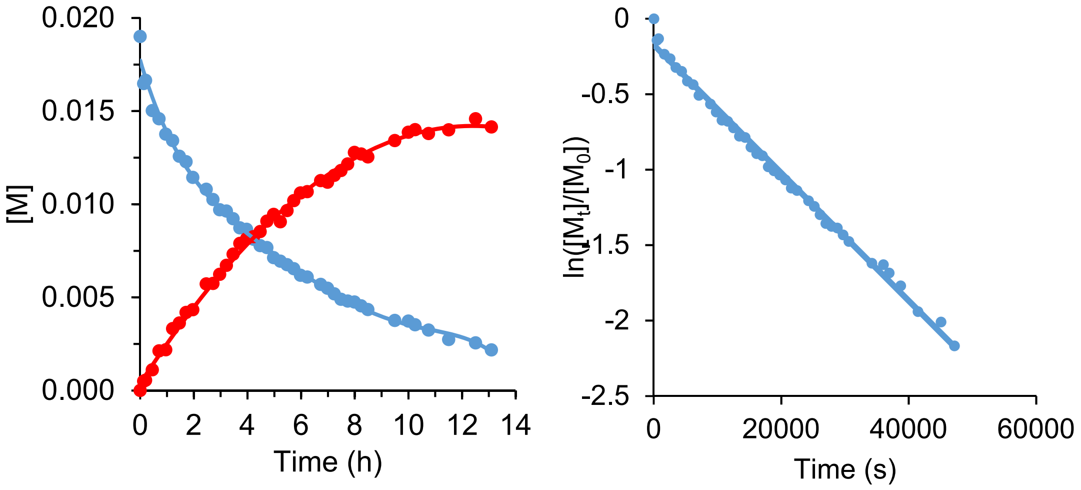

Figure S50. Reaction of **1** (0.019 M) with Et_3SiH (0.38 M) in toluene-d_8 at 353 K.

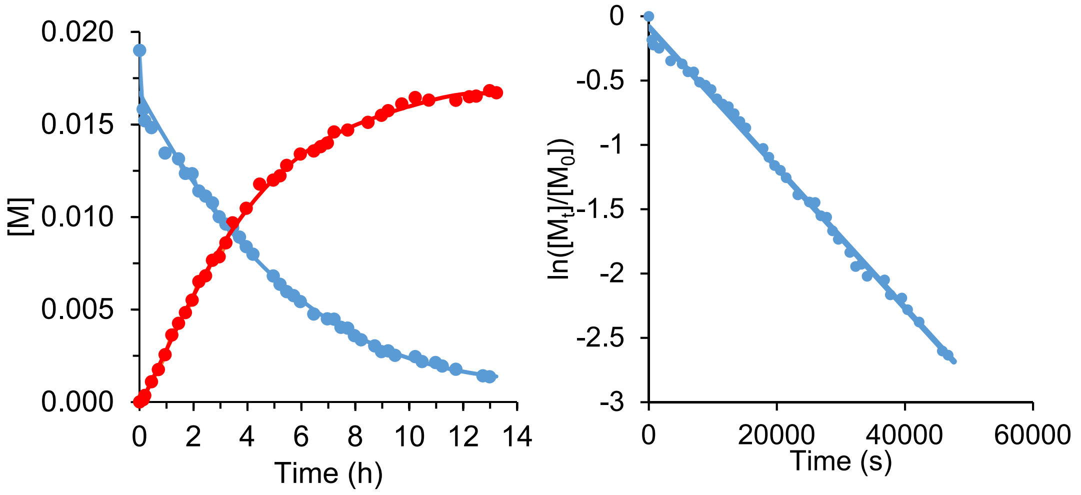

Figure S51. Reaction of **1** (0.019 M) with Et_3SiH (0.48 M) in toluene-d_8 at 353 K.

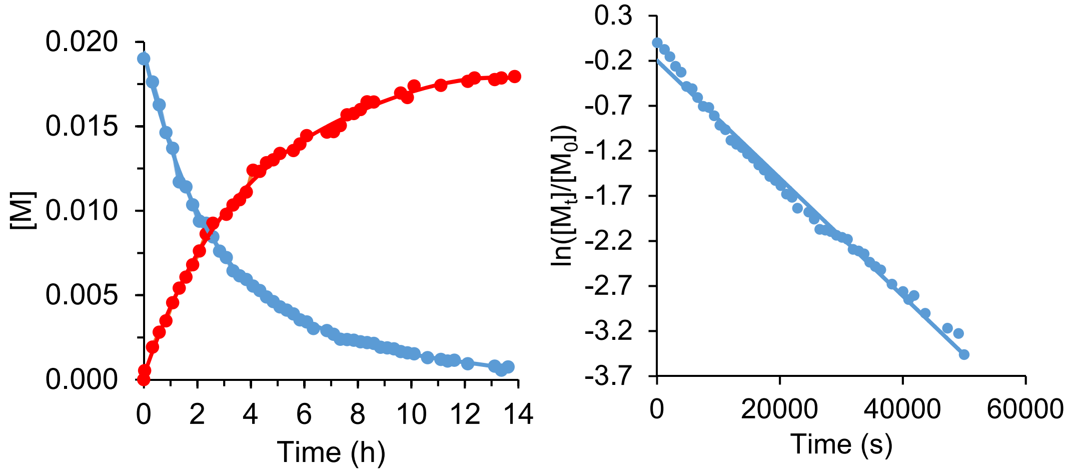

Figure S52. Reaction of **1** (0.019 M) with Et_3SiH (0.57 M) in toluene-d_8 at 353 K.

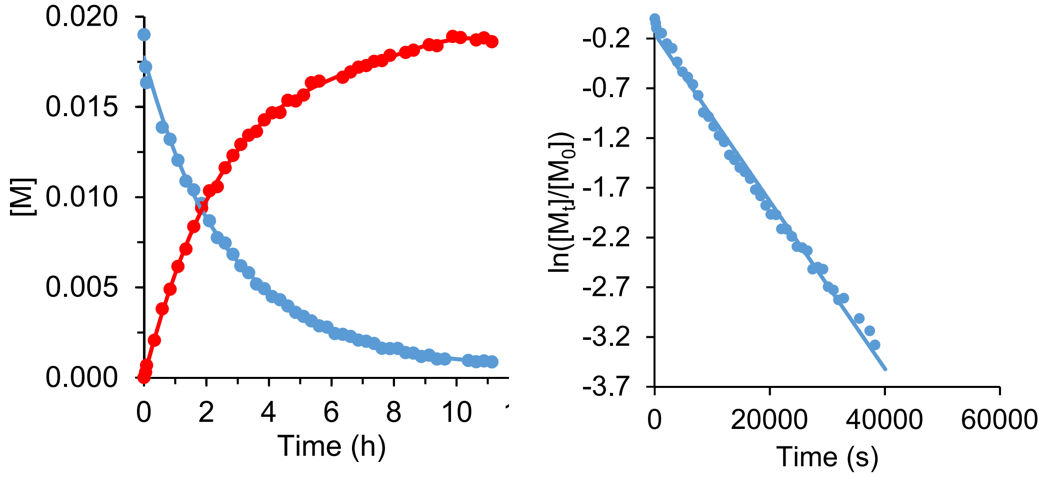

Figure S53. Reaction of **1** (0.019 M) with Et_3SiH (0.76 M) in toluene-d_8 at 353 K.

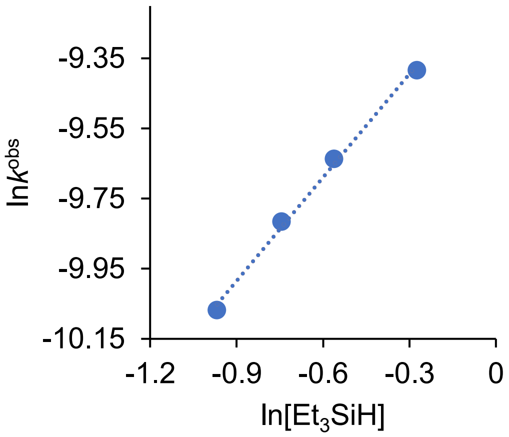

Figure S54. Plot of lnk^{obs} versus ln[Et_3SiH] for the reaction of **1** (0.019 M) with Et_3SiH in toluene-d_8 at 353 K.

– **References**

(1) Alós, J.; Bolaño, T.; Esteruelas, M. A.; Oliván, M.; Oñate, E.; Valencia, M. POP-Pincer Osmium-Polyhydrides: Head-to-Head (*Z*)-Dimerization of Terminal Alkynes. *Inorg. Chem.* **2013**, *52*, 6199–6213.

(2) Blessing, R. H. *Acta Crystallogr.* **1995**, *A51*, 33. SADABS: Area-detector absorption correction; Bruker- AXS, Madison, WI, 1996.

(3) SHELXL-2016/6. Sheldrick, G. M. *Acta Cryst.* **2008**, *A64*, 112–122.

(4) (a) Lee, C.; Yang, W.; Parr, R. G. Development of the Colle-Salvetti correlationenergy formula into a functional of the electron density. *Phys. Rev. B* **1988**, *37*, 785–789. (b) Becke, A. D. Density-functional exchange-energy approximation with correct asymptotic behavior. *J. Chem. Phys.* **1993**, *98*, 5648–5652. (c) Stephens, P. J.; Devlin, F. J.; Chabalowski, C. F.; Frisch, M. J. Ab Initio Calculation of Vibrational Absorption and Circular Dichroism Spectra Using Density Functional Force Fields. *J. Phys. Chem.* **1994**, *98*, 11623–11627.

(5) Grimme, S.; Antony, J.; Ehrlich, S.; Krieg, H. A consistent and accurate ab initio parametrization of density functional dispersion correction (DFT-D) for the 94 elements H-Pu. *J. Chem. Phys.* **2010**, *132*, 154104–154123.

(6) Gaussian 09, Revision D.01, Frisch, M. J.; Trucks, G. W.; Schlegel H. B.; Scuseria, G. E.; Robb, M. A.; Cheeseman, J. R.; Scalmani, G.; Barone, V.; Mennucci, B.; Petersson, G. A.; Nakatsuji, H.; Caricato, M.; Li, X.; Hratchian, H. P.; Izmaylov, A. F.; Bloino, J.; Zheng, G.; Sonnenberg, J. L.; Hada, M.; Ehara, M.; Toyota, K.; Fukuda, R.; Hasegawa, J.; Ishida, M.; Nakajima, T.;

Honda, Y.; Kitao, O.; Nakai, H.; Vreven, T.; Montgomery, J. A.; Peralta, Jr., J. E.; Ogliaro, F.; Bearpark, M.; Heyd, J. J.; Brothers, E.; Kudin, K. N.; Staroverov, V. N.; Keith, T.; Kobayashi, R.; Normand, J.; Raghavachari, K.; Rendell, A.; Burant, J. C.; Iyengar, S. S.; Tomasi, J.; Cossi, M.; Rega, N.; S43 Millam, J. M.; Klene, M.; Knox, J. E.; Cross, J. B.; Bakken, V.; Adamo, C.; Jaramillo, J.; Gomperts, R.; Stratmann, R. E.; Yazyev, O.; Austin, A. J.; Cammi, R.; Pomelli, C.; Ochterski, J. W.; Martin, R. L.; Morokuma, K.; Zakrzewski, V. G.; Voth, G. A.; Salvador, P.; Dannenberg, J. J.; Dapprich, S.; Daniels, A. D.; Farkas, O.; Foresman, J. B.; Ortiz, J. V.; Cioslowski, J.; Fox, D. J. Gaussian, Inc., Wallingford CT, 2013.

(7) Andrea, D.; Häußermann, U. M.; Dolg, M.; Stoll, H.; Preuss, H. Energyadjusted ab initio pseudopotentials for the second and third row transition elements. *Theor. Chim. Acta* **1990**, *77*, 123–141.

(8) Ehlers, A. W.; Bohme, M.; Dapprich, S.; Gobbi, A.; Hollwarth, A.; Jonas, V.; Kohler, K. F.; Stegmann, R.; Veldkamp, A.; Frenking, G. A set of f-polarization functions for pseudo-potential basis sets of the transition metals SC-Cu, Y-Ag and La-Au. *Chem. Phys. Lett.* **1993**, *208*, 111–114.

(9) (a) Hehre, W. J.; Ditchfield, R.; Pople, J. A. Self-Consistent Molecular Orbital Methods. XII. Further Extensions of Gaussian-Type Basis Sets for Use in Molecular Orbital Studies of Organic Molecules. *J. Chem. Phys.* **1972**, *56*, 2257–2261. (b) Francl, M. M.; Pietro, W. J.; Hehre, W. J.; Binkley, J. S.; Gordon, M. S.; DeFrees, D. J.; Pople, J. A. Self-consistent molecular orbital methods. XXIII. A polarization-type basis set for second-row elements. *J. Chem. Phys.* **1982**, *77*, 3654–3665.

(10) AIMAll (Version 15.09.27), Todd A. Keith, TK Gristmill Software, Overland Park KS, USA, 2016, (aim.tkgristmill.com).
